# Preoperative assessment system for hand-assisted laparoscopic donor nephrectomy by discriminant analysis

**DOI:** 10.1371/journal.pone.0227546

**Published:** 2020-04-28

**Authors:** Kazuhiro Iwadoh, Ichiro Nakajima, Ichiro Koyama, Kosaku Nitta, Shohei Fuchinoue

**Affiliations:** 1 Departments of Surgery III, Tokyo Women’s Medical University, Tokyo, Japan; 2 Departments of Nephrology, Tokyo Women’s Medical University, Tokyo, Japan; University of Colorado, Anschutz Medical Campus, UNITED STATES

## Abstract

We developed a preoperative assessment system to predict surgical workload in hand-assisted laparoscopic donor nephrectomy (HALDNx) using the normal-based linear discriminant rule (NLDR). A total of 128 cases of left HALDNx performed by a single operator were used as training data. Surgical workload was measured by operative time. The optimized model had 9 explanatory variables: age, total protein, total cholesterol, number of renal arteries (numberRA), 4 variables of perinephric fat (PNF), and thickness of subcutaneous fat. This model was validated using cross-validation and the .632 estimator to estimate discrimination rates with future test data. PNF and numberRA were the predominant factors affecting workload followed by the computed tomography value of PNF, body weight, and male sex. The estimated accuracy of the prediction system was 94.6%. The complication rate was 9.38% and did not correlate with surgical workload. We also made our program available online for constructing assessment functions from other cohort data. In conclusion, the surgical workload of HALDNx could be predicted with PNF and numberRA as the dominant risk factors.

## Introduction

Laparoscopic nephrectomy was first introduced by Clayman et al. [1] in 1991. The first successes in standard laparoscopic live donor nephrectomy by Ratner and Kavoussi [2] in 1995 and in hand-assisted laparoscopic donor nephrectomy (HALDNx) by Wolf [3] in 1998 have proven the minimal invasiveness of these procedures compared with open donor nephrectomy, including lower morbidity, shorter hospitalization, and rapid convalescence [4–8]. Initially, laparoscopic live donor nephrectomy was developed to remove disincentives for live kidney donation in the hope of increasing the number of live kidney donors [2, 5, 6]. The hand-assisted laparoscopic method was then developed to achieve safety and speed of surgical procedures similar to those obtainable with the open method [3]. Thereafter, many reports have demonstrated that laparoscopic approaches have achieved graft and patient survival rates comparable to those of conventional open approaches [9–12].

However, there seem to be lingering issues surrounding the laparoscopic approaches that need to be addressed, principal among which are the presence of a learning curve in the early stage of its introduction and the high level of surgical safety required at every moment in donor nephrectomy, even long after its safe introduction. Laparoscopic minimally invasive techniques must be approached very cautiously amid the learning curve [7]. Gaston et al. have reported that the hand-assisted approach has a relatively shorter learning curve than the standard approach, as reflected in a rapid decrease in operative time in the setting of a residency training program in urology [13]. However, the safe implementation of HALDNx is only the beginning of persistent efforts to ensure its safety.

HALDNx was first introduced in our Department of Surgery III at Tokyo Women's Medical University in February 2001. Since then, over 1300 cases of HALDNx have been completed without any conversion to open surgery or any need for blood transfusion [14–16]. Our series has demonstrated that HALDNx can be performed quite safely with minimal morbidity, while also empirically providing the experienced operator a rough notion of a particular donor type in whom HALDNx could be time consuming, specifically, a middle-aged, overweight man with excessive perinephric fat (PNF) that could be a challenging case for novice and experienced laparoscopists alike.

We, therefore, hypothesized that the physical structure of donors in addition to the amount of PNF or the number of renal arteries (numberRA) might help to discriminate the operative workload of HALDNx as measured by operative time. We used donors’ demographic and laboratory data in addition to imaging information from abdominal computed tomography (CT) scans to collect independent variables for predictive discriminant analysis (PDA) of surgical workload. Such a preoperative evaluation system might enable the selection of an appropriate laparoscopic surgeon for a donor based on the predicted surgical workload, and the development of a strategy for a novice laparoscopist to pass through the learning curve safely by selecting donors with lower expected surgical workload.

In this report, we describe a preoperative system for evaluation of surgical workload in HALDNx to demonstrate the possibility of calibrating operative difficulty using a discrimination index formulated for each donor and predicting whether HALDNx is expected to be time-consuming.

## Results

The distributions of operative time and 16 clinical variables of 128 donors are shown in Table 1. Median operative time was 150 min (range: 90–360 min). Mean body mass index (BMI) was 23.4 ± 3.3 kg/m^2^ (range: 16.6–35.5 kg/ m^2^).

**Table 1:** Distributions of clinical variables of donors.

Variables	Mean ± SD	Range	N
Operative time (min)	159.1 ± 47.4	90–360	128
Male / Female (1/0)	M: 58 / F: 70	—	128
Age (years)	55.96 ± 10.65	21–76	128
Body weight (kg)	60.87 ± 11.81	34–99	128
Body height (cm)	160.82 ± 8.73	142–180	128
Total protein (g/dL)	7.09 ± 0.41	6.2–8.6	128
Albumin (g/dL)	4.27 ± 0.25	3.6–4.9	128
Triglyceride (mg/dL)	144.10 ± 97.59	35–538	128
Total cholesterol (mg/dL)	206.45 ± 32.68	122–291	128
numberRA	1.43 ± 0.71	1–4	128
maxthickMPF (mm)	8.36 ± 5.00	1.80–22.61	128
medthickMPF (mm)	6.92 ± 4.71	0.60–23.98	128
medthickLPF (mm)	13.25 ± 6.89	3.00–36.78	128
areaPNF (cm^2^)	14.39 ± 10.98	1.22–55.19	128
ctvPNF	-83.98 ± 12.17	-103.37–51.62	128
thickSCF (mm)	17.50 ± 7.34	2.69–42.82	128
areaSCF (cm^2^)	92.01 ± 39.61	16.82–254.96	128

numberRA, number of renal arteries of the graft; maxthickMPF, maximum thickness of medial perinephric fat; medthickMPF, median thickness of medial perinephric fat; medthickLPF, median thickness of lateral perinephric fat; areaPNF, area of perinephric fat; ctvPNF, CT value of perinephric fat density; thickSCF, thickness of subcutaneous fat at abdominal midline; areaSCF, area of subcutaneous fat.

The anatomical meanings of these parameters are shown in S1A Fig in S1 File.

Results of linear regression analysis of operative time against clinical variables are summarized in Table 2. Univariate linear regression revealed that male sex, body weight (Bw), height (Ht), triglyceride (TG) level, numberRA of the graft, maximum and median thicknesses of medial perinephric fat (maxthickMPF, medthickMPF), median thickness of lateral perinephric fat (medthickLPF), area of perinephric fat (areaPNF), CT value of perinephric fat density (ctvPNF), and area of subcutaneous fat (areaSCF) were all significantly correlated with operative time. In short, univariate analyses revealed that the donor parameters of physical frame, perinephric structures (e.g., adipose tissue and renal artery), and sex (male) were all correlated with operative workload. In multivariate linear regression, age, albumin (Alb) level, numberRA, and areaPNF were significantly correlated with operative time. Therefore, only numberRA and areaPNF were significant in both univariate and multivariate analyses; their *p*-values in multivariate analysis were less than 0.001, indicating that they were the predominant determinants of operative workload, according to the linear regression method. Total protein (TP) level, total cholesterol (TC) level, and thickness of subcutaneous abdominal fat at the midline (thickSCF) were not significantly correlated with operative time.

**Table 2:** Univariate and multivariate linear regression of operative time against 16 clinical variables.

Variable	Univariate			Multivariate		
	coefficient	*p*-value	N	coefficient	*p*-value	N
Male*	37.11	< 10−5	128	-14.07	0.208	128
Age**	0.487	0.219	128	-0.808	0.016	128
Body weight*	2.397	< 10−12	128	0.481	0.478	128
Body height*	1.670	0.00042	128	-0.226	0.734	128
Total protein	8.108	0.435	128	7.897	0.329	128
Albumin**	-7.343	0.665	128	-34.82	0.016	128
Triglyceride*	0.133	0.00018	128	0.025	0.463	128
Total cholesterol	-0.102	0.429	128	-0.055	0.557	128
numberRA* **	20.82	0.00037	128	17.66	0.000071	128
maxthickMPF*	6.477	0.	128	1.556	0.334	128
medthickMPF*	7.013	0.	128	0.059	0.974	128
medthickLPF*	4.869	< 10−15	128	-0.058	0.948	128
areaPNF* **	3.179	0.	128	2.842	0.00034	128
ctvPNF*	-1.794	< 10−7	128	0.223	0.532	128
thickSCF	0.588	0.307	128	0.088	0.907	128
areaSCF*	0.318	0.0024	128	-0.033	0.858	128

Variables with * were significant risk factors in univariate linear regression and those with ** were significant in multivariate regression.

numberRA, number of renal arteries of the graft; maxthickMPF, maximum thickness of medial perinephric fat; medthickMPF, median thickness of medial perinephric fat; medthickLPF, median thickness of lateral perinephric fat; areaPNF, area of perinephric fat; ctvPNF, CT value of perinephric fat density; thickSCF, thickness of subcutaneous abdominal fat at the midline; areaSCF, area of subcutaneous fat.

The anatomical meanings of these parameters are shown in S1A Fig in S1 File.

We applied the normal-based linear discriminant rule (NLDR) to the training dataset with one target variable (operative time) and a certain number of explanatory variables, to create a discriminant rule that was expected to distinguish between cases with operative times of 210 min or longer and those that were shorter. We performed machine learning to select the best combination of explanatory variables in the last step.

Prediction rates according to the NLDR applied to the training data with different subsets of explanatory variables are shown in Table 3. The results of Set A (= sex, age, Bw, Ht) revealed that NLDR attained a total hit rate as high as 86.7% with only 4 parameters of demographic data with high specificity. Set A comprised the first subsets of parameters we chose in developing discriminant rules in HALNx. Set B indicated that adding 4 metabolite variables improved the total hit rate only slightly. Set B (= Set A plus TP, Alb, TG, and TC) was the second subset of parameters we chose in order to improve the discriminant rates by adding information about 4 metabolites in donors. The addition of these variables improved the total hit rate only slightly and a weakness of Set B was that it could not discriminate difficult cases with an ordinary physical frame. Therefore, we added more perinephric information (numberRA, maxthickMPF, medthickMPF, medthickLPF, areaPNF, and ctvPNF) obtained from donors’ abdominal CT images to Set B to create Set C, which resulted in marked improvement in all discrimination rates. The hit rate of difficult cases (HRDC) was improved from 80.0% with Set B to 100% with Set C, and a 93.0% hit rate of easy cases (HREC) and a 93.8% total hit rate were achieved. In Set D (Set C plus thickSCF and areaSCF), we examined the effect of subcutaneous abdominal fat) on hit rates and found no change compared with Set C.

**Table 3:** Results of PDA by NLDR on training data.

Variable	Set A	Set B	Set C	Set D	Set E
Male	●	●	●	●	
Age	●	●	●	●	●
Body weight	●	●	●	●	
Body height	●	●	●	●	
Total protein		●	●	●	●
Albumin		●	●	●	
Triglyceride		●	●	●	
Total cholesterol		●	●	●	●
numberRA			●	●	●
maxthickMPF			●	●	●
medthickMPF			●	●	●
medthickLPF			●	●	
areaPNF			●	●	●
ctvPNF			●	●	●
thickSCF				●	
areaSCF				●	●
N	128	128	128	128	128
Hit rate of difficult cases: DRDC	83.3%	80.0%	100%	100%	100%
Hit rate of easy cases: DREC	86.9%	89.0%	93.0%	93.0%	95.5%
Sensitivity	23.8%	38.1%	61.9%	61.9%	76.2%
Specificity	99.1%	98.1%	100%	100%	100%
Total hit rate	86.7%	88.3%	93.8%	93.8%	96.1%

PDA, predictive discriminant analysis; NLDR, normal-based linear discriminant rule; numberRA, number of renal arteries of the graft; maxthickMPF, maximum thickness of medial perinephric fat; medthickMPF, median thickness of medial perinephric fat; medthickLPF, median thickness of lateral perinephric fat; areaPNF, area of perinephric fat; ctvPNF, CT value of perinephric fat density; thickSCF, thickness of subcutaneous abdominal fat at the midline; areaSCF, area of subcutaneous fat.

The anatomical meanings of these parameters are shown in S1A Fig in S1 File.

We used machine learning to identify subsets of explanatory variables with the highest hit rates. The NLDR was applied to all 65,535 possible subsets derived from the 16 clinical variables; 395 subsets (0.603%) returned the same 2 × 2 table with a total hit rate of 96.1%, HREC of 95.5%, and HRDC of 100% (S1 Text). Set E is just one of these (Table 3). The reason for selecting Set E from 395 sets was that it was the only one showing the highest hit rate both by the cross-validation and the .632 estimator. These two validations were undertaken to estimate the true hit rates of each subset with NLDR if it were applied to future test data of donors.

The outcomes of both cross-validation and the .632 estimator with NLDR to Sets A, B, C, and D are shown in Tables 4 and 5. Set C had perinephric information and gave the highest total hit rate of 94.5%, with HRDC of 88.9% and HREC of 95.5%. With the .632 estimator, however, both Sets C and D showed the highest total hit rate of 93.2%, while the discrimination rates of Sets A and B were lower or nearly equivalent.

**Table 4:** Results of PDA by cross-validation with NLDR.

Variable	Set A	Set B	Set C	Set D	Set E
Male	●	●	●	●	
Age	●	●	●	●	●
Body weight	●	●	●	●	
Body height	●	●	●	●	
Total protein		●	●	●	●
Albumin		●	●	●	
Triglyceride		●	●	●	
Total cholesterol		●	●	●	●
numberRA			●	●	●
maxthickMPF			●	●	●
medthickMPF			●	●	●
medthickLPF			●	●	
areaPNF			●	●	●
ctvPNF			●	●	●
thickSCF				●	
areaSCF				●	●
N	128	128	128	128	128
Hit rate of difficult cases: DRDC	62.5%	60.0%	88.9%	83.3%	100%
Hit rate of easy cases: DREC	90.2%	91.7%	95.5%	94.6%	95.5%
Sensitivity	47.6%	57.1%	76.2%	71.4%	76.2%
Specificity	94.4%	92.5%	98.1%	97.2%	100%
Estimated total hit rate	86.7%	86.7%	94.5%	93.0%	96.1%

PDA, predictive discriminant analysis; NLDR, normal-based linear discriminant rule;

numberRA, number of renal arteries of the graft; maxthickMPF, maximum thickness of medial perinephric fat; medthickMPF, median thickness of medial perinephric fat; medthickLPF. median thickness of lateral perinephric fat; areaPNF, area of perinephric fat; ctvPNF, CT value of perinephric fat density; thickSCF, thickness of subcutaneous abdominal fat at the midline; areaSCF, area of subcutaneous fat.

The anatomical meanings of these parameters are shown in S1A Fig in S1 File.

**Table 5:** Results of PDA by the .632 estimator with NLDR.

Variable	Set A	Set B	Set C	Set D	Set E
Male	●	●	●	●	
Age	●	●	●	●	●
Body weight	●	●	●	●	
Body height	●	●	●	●	
Total protein		●	●	●	●
Albumin		●	●	●	
Triglyceride		●	●	●	
Total cholesterol		●	●	●	●
numberRA			●	●	●
maxthickMPF			●	●	●
medthickMPF			●	●	●
medthickLPF			●	●	
areaPNF			●	●	●
ctvPNF			●	●	●
thickSCF				●	
areaSCF				●	●
N	128	128	128	128	128
Hit rate of difficult cases: DRDC	60.0%	54.1%	91.3%	89.2%	95.5%
Hit rate of easy cases: DREC	90.0%	90.1%	93.7%	94.0%	94.5%
Sensitivity	45.9%	45.8%	66.1%	67.3%	69.9%
Specificity	90.4%	91.1%	98.6%	98.3%	99.4%
Estimated total hit rate	83.0%	83.6%	93.2%	93.2%	94.6%

PDA, predictive discriminant analysis; NLDR, normal-based linear discriminant rule; numberRA, number of renal arteries of the graft; maxthickMPF, maximum thickness of medial perinephric fat; medthickMPF, median thickness of medial perinephric fat; medthickLPF, median thickness of lateral perinephric fat; areaPNF, area of perinephric fat; ctvPNF, CT value of perinephric fat density; thickSCF, thickness of subcutaneous fat at abdominal midline; areaSCF, area of subcutaneous fat.

To select the most appropriate subsets for obtaining the highest possible hit rates, cross-validation was applied to the previously selected 395 subsets. Set E had the highest total hit rate of 96.1% and 27 other subsets had total hit rates between 94.5% and 95.3% (S2 Text). Finally, the .632 estimator was applied to Set E plus these 27 subsets and Set E again returned the highest total hit rate of 94.6% with HRDC of 95.5% and HREC of 94.5% (S3 Text) with our cohort used as the parent population.

**Fig 1:**
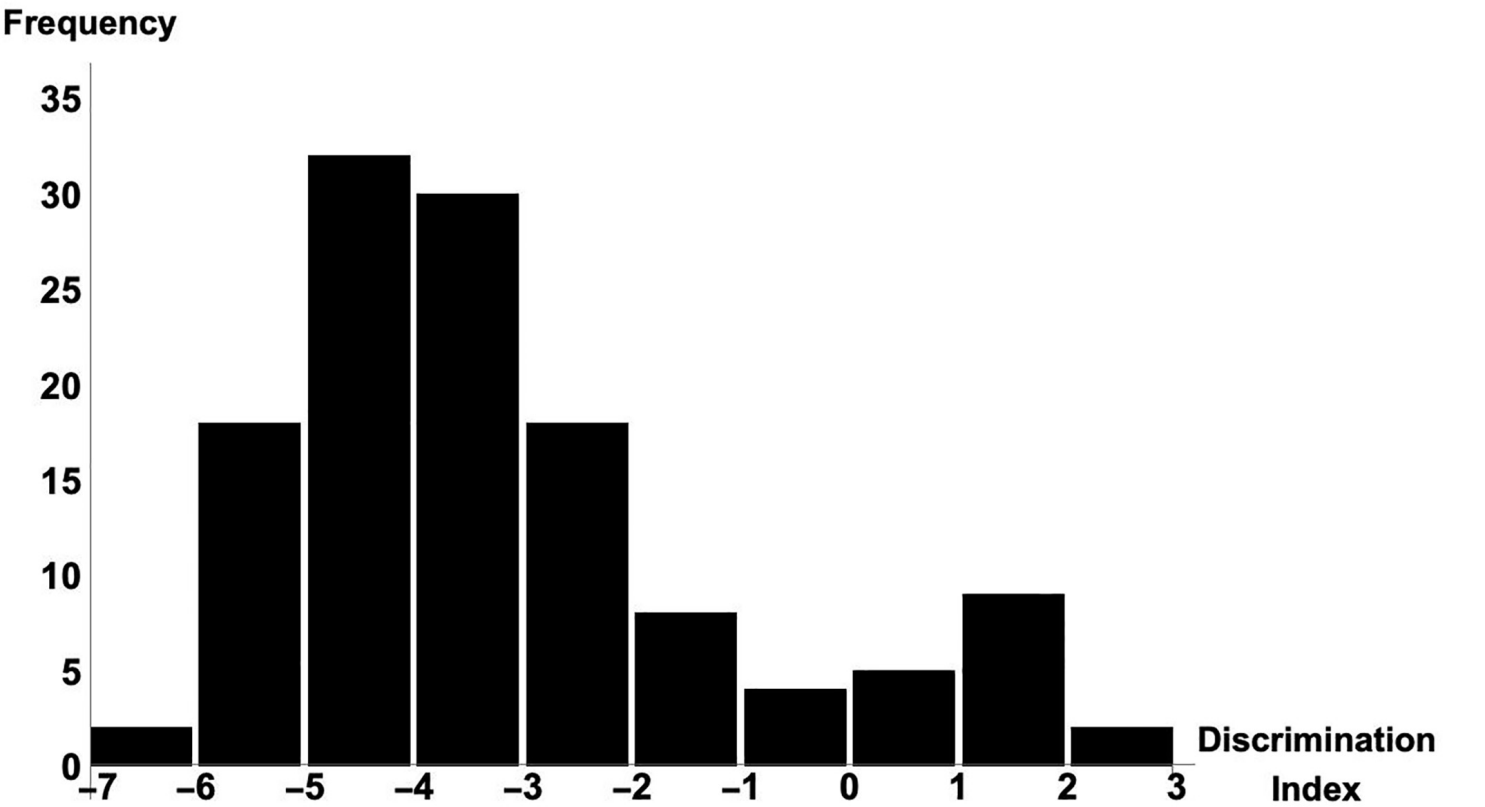
Histogram of discrimination index by NLDR with Set E. Distribution of discrimination indexes of 128 donors calculated by NLDR using the training data of Set E ranged from −6.77 to 2.14. Horizontal axis is the index value and vertical axis is its frequency.

Fig 1 shows the distribution of the discrimination index calculated using NLDR with Set E over 128 donors; the higher the index, the more difficult the HALDNx was likely to be. A positive index indicates a possibly difficult case with a heavy surgical workload. S1B Fig in S1 File is a representative cross-sectional abdominal image of the donor with the highest discrimination index of 2.14; operative time was 270 min. S1C Fig in S1 File is that of the donor with the lowest index of −6.77; operative time was 120 min. The difference in the amount of PNF between these cases is obvious.

S1D Fig in S1 File is a representative abdominal image of a 47-year-old female donor whose BMI, Bw, and Ht were 29.64 kg/m^2^, 81 kg, and 165 cm, respectively. Although she was nearly obese, S1D Fig in S1 File shows peripheral pear-shaped adiposity, in which PNF was not so abundant while subcutaneous fat was very thick. Her discrimination index was −2.30 and operative time was short, at only 120 min. A donor with central apple-shaped adiposity, on the other hand, could possibly present a heavy workload. S1E Fig in S1 File shows a representative abdominal image of a 61-year-old man who had large amounts of perinephric but minimal subcutaneous fat. His operative time was up to 360 min and the discrimination index was 1.93. S1E Fig in S1 File, therefore, clearly shows that a kidney that appears like "a bean floating in the lake of perinephric fat" could be a warning sign of heavy operative workload in HALDNx. These cases taught us that neither BMI nor Bw was a definitive indicator of workload.

Finally, we performed more clinically-detailed analyses to elucidate the most important factors influencing operative time and surgical complications. Pearson's correlation coefficient of each factor with the operative time revealed that areaPNF was the most influential factor on workload, followed by perinephric characteristics like medthickMPF, maxthickMPF, and medthickLPF (S1F Fig in S1 File), while ctvPNF was negatively correlated and its absolute value was the sixth largest next to Bw. In contrast, subcutaneous fat and metabolites were only slightly correlated. We also compared discrimination indexes, predictive factors, and surgical complications between the heaviest and lightest workload cases (S1B Table in S2 File), which was about 10 cases in each subset. The most significantly different factors between the two subsets were discrimination index, maththickMPF, areaPNF, and ctvPNF (all *p* = 0.000169). At the same time, no significant differences were found in surgical complication grade based on the modified Clavien classification, thickSCF, AT, Alb, or TC [17].

Surgical complications in 128 cases of HALDNx are summarized in S1C Table in S2 File. They are classified according to the modified Clavien system [17]. There were 12 cases (9.38%) with complications of grade 1 or higher. Six patients had Grade 1 complications: 1 case of constipation and 5 cases of postoperative pain. Four patients had Grade 2a complications, all of which were surgical site infections. Two patients developed Grade 2b complications: abdominal hernia and pneumothorax, respectively. To examine the relations between complications and predictive factors, we used Pearson's correlation coefficients of complications with predictive factors (S1G Fig in S1 File). Although the values of coefficients were relatively small, areaSCF and thickSCF were most prominent. Five patients had postoperative pain; their operative times were 330, 240, 210, 150, and 150 min. We also examined the Spearman's correlation coefficient between the discrimination indexes of workload and the Clavien complication classification system (S1H Fig in S1 File) [17]. There was no significant correlation between them (p = 0.785).

## Discussion

To ensure the safety of HALDNx, we developed a preoperative assessment system of surgical difficulty of live donors in terms of operative workload measured by operative time. The system represents the workload as a discrimination index defined using a statistical NLDR method based on donor's predictive variables.

This system revealed that the amount of PNF was the predominant factor influencing the operative workload and operative time. Other factors, in order of importance were numberRA, ctvPNF, Bw, and male sex. A typical case of heavy workload was an overweight, middle-aged man with apple-shaped adiposity. On the other hand, most of these factors did not affect the surgical complication rate. The discrimination index was also not significantly correlated with complication gradings.

Laparoscopic live donor nephrectomy is a technique that opened a new means of live kidney donation. Many transplant surgeons have shared a belief in the efficacy and safety of the laparoscopic donor nephrectomy [6, 9, 10]. However, operative safety in donor nephrectomy must be maintained at the highest level in clinical practice as long as it is performed. As Flowers and colleagues noted, donor nephrectomy is a rare major operation in the sense that an otherwise healthy patient is placed at potential surgical risk for altruistic reasons [7, 12, 18]. In addition, laparoscopic donor nephrectomy is a surgically demanding procedure [19]. There is evidently a learning curve that exists in developing the laparoscopic technique [6, 8, 18, 20]. Furthermore, it could be challenging in very obese donors, those with complicated renal hilar vasculature, and those with hard sticky PNF [1,7, 18, 21].

Therefore, every available means should be utilized to minimize major complications and risk of mortality [19]. Under these circumstances, laparoscopists experienced in live donor procedures should be empirically aware of certain groups of patients requiring heavy surgical workload who could be distinguished based on their physical structure or abdominal CT imaging findings [22]. Since 2004, we have attempted to predict the surgical workload in HALDNx through clinical parameters of donors [15, 16]. Donor nephrectomy is very unique in the sense that it is essentially performed on a person without any disease in the abdomen at the time of operation. If the patient had abdominal disease, such as malignant tumor or severe inflammation, there would be several other unexpected risk factors related to underlying pathological conditions specific to each patient, making it difficult or impossible to predict the operative workload beforehand.

Ratner et al. first addressed the importance of preoperative evaluation of operative difficulty in laparoscopic live donor nephrectomy in 2000 [23]. He developed grading scores for operative difficulty in laparoscopic donor nephrectomy and searched for predictive factors in clinical parameters that correlated with the scores. Only operative time significantly correlated with the total operative difficulty score and in turn significantly correlated with donor weight and abdominal girth. In the present study, multivariate linear regression revealed that numberRA and areaPNF were markedly correlated with operative time (Table 2). These findings imply that both general physique and perinephric anatomy could be indicators of surgical workload.

After testing several methods, we found that the NLDR was the most effective way to conduct PDA for our issue [24, 25]. S1A Table in S2 File shows a comparison of discrimination rates using different discriminators. The results of NLDR with Sets A and B suggest that 87%–89% of easy cases could be discriminated by only the general physical features of patients and variables on metabolites (Table 3). For example, a donor with low BMI seldom (but not rarely) presents as a difficult case, while one with high BMI could turn out to be either difficult or easy depending on other intra-abdominal risk factors [25, 26]. Therefore, a more individualized analysis with local anatomical information seemed to be required to estimate the operative workload for a particular patient with high accuracy comparable to the judgement of an experienced surgeon.

We then incorporated the anatomical variables of local perinephric structure into the analysis as Set C, since we speculated that the direct measurement of PNF was indispensable for accurate evaluation. The results of Sets C and D showed a marked improvement in HRDC, suggesting that with the local anatomical information supplied, it could be possible to discriminate between cases of obese build with little PNF and those with an abundance of fat [26]. Anderson et al. first reported the effect of perirenal fat on operative time and complexity of procedures during HALDNx [22]. They showed a significant correlation between anterior and posterior perirenal fat and operative time, which supports our result.

We then conducted a large-scale analysis using machine learning with all 65,535 possible subsets out of 16 clinical variables to discover the combination of predictive factors with the highest hit rate. Finally, there were 395 subsets, including Set E, with the same highest total hit rate when NLDR was applied to our cohort (S1 Text). This implied that there was a certain amount of combinations of parameters with similar high hit rates. This is likely due to the fact that several variables were intercorrelated and some could be replaced with others.

The application of cross-validation over those 395 subsets identified Set E as the one with the highest total hit rate of 96.1% (S2 Text). Its risk factors mainly comprised those of PNF, such as thickness, area, and mean CT density value. Unexpectedly, both Ht and Bw were not included, indicating that PNF or regional anatomy was the most predominant risk factor for surgical workload in HALDNx. It would be also probable that information concerning Bw was compensated for by other factors such as PNF. Cross-validation analysis also revealed 3 other subsets with a total hit rate of 95.3% and 24 with a total hit rate of 94.5%, which were slightly lower than the total hit rate with Set E (S2 Text).

Then we applied the .632 estimator to the previous Sets A, B, C, and D and these 28 subsets including Set E to further estimate their hit rates for future data (S3 Text). Set E again showed the best statistical performance (Table 5). Set E, therefore, was determined by a purely statistical process to realize a model with the highest discrimination rates based on the present cohort. Even if dozens of models had similar hit rates, one must be selected for future clinical application and it should contain a set of essential predictive factors. Combined with the statistical analyses and other methods in this study, numberRA, areaPNF, ctvPNF, maxthickMPF, and medthickMPF are very important factors. Factors other than these five would have also been included to help Set E show the highest hit rate in the validation process when the present cohort is used as the training dataset.

We therefore concluded that the operative difficulty of HALDNx could be predicted by NLDR for future cases with high accuracy, which in our cohorts was an estimated total hit rate of 94.6% with 95.5% of HRDC and 94.5% of HREC using parameters in Set E.

With the statistical selection of Set E as the best classifier of operative workload, the operative difficulty of any case can be represented as a discrimination index using NLDR with Set E, as shown in Fig 1. We then further investigated the most influential predictive factors by using the index and the operative time. We also investigated those factors that influenced surgical complications and the relationship between the index and the complication rate.

We compared cases with operative times of 240 min or more with those of 100 min or less, as in S1B Table in S2 File. The most significantly between-case differences were observed for maxthickPNF, areaPNF, ctvPNF, and discrimination index (all *p* = 0.000169). This implied that the amount of PNF was the main determinant of the workload. The case with the longest operative time (360 min) had the greatest areaPNF (55.19 mm^3^) of all cases. The 2 cases with the shortest time (90 min) were women of short stature (about 150 cm) whose maxthickMPF and medhtickMPF were small while their areaSCF was greater than average, indicating that the amount of subcutaneous fat did not greatly affect the operative time.

We next calculated Pearson's correlation coefficients of operative time using 16 predictive factors. The correlation coefficient with areaPNF was as high as 0.736, which was the highest among the other 15 variables, as shown in S1F Fig S1 File. Therefore, its coefficient of determination (R^2^) is 0.543, which means that more than half of the workload could be explained by PNF. The amount or type of PNF influenced the operative time and difficulty of HALNx. A typical challenging case was a middle-aged overweight man in his 50s or 60s. Such a patient often had a large amount of PNF or an apple-shaped adiposity, which was sometimes rather hard and sticky and therefore difficult to dissect. A combination of thick PNF with the presence of more than three renal arteries would be one of the most difficult cases in HALDNx.

The biggest hazard in HALNx is sudden massive bleeding. Large amounts of PNF are not only time-consuming to deal with but also pose a risk for bleeding because thick adipose tissue makes it difficult to search for a branch of the renal artery located on its posterior aspect. An artery outside the view of the endoscopic camera is always a risk factor. The parameter numberRA is another predominant risk factor. One or two renal arteries are commonly encountered but are rarely cause for concern: however, when there are four or more, the risk is much greater. If the patient has four renal arteries with thick PNF, open surgery might be a good option. Such a case almost always shows the highest discrimination index in the preoperative assessment compared with the cases in the training dataset.

The correlation coefficients of surgical complications with predictive factors were relatively low, as shown in S1G Fig in S1 File. The highest were thickSCF and areaSCF, which is likely due to the fact that 4 of the 12 complications were surgical site infections and another was abdominal incisional hernia. Still another complication with high frequency was pain in the shoulder, flank, or lower limbs, which is likely due to the lateral decubitus position used in HALDNx. There were 5 cases with such pain, and their operative times were 330, 240, 210, 150, and 150 min. The remaining complications were constipation, pneumothorax, and hernia.

The correlations between the discrimination indexes and the complication gradings are shown in S1H Fig in S1 File. Higher index scores did not necessarily mean higher complication grades, as shown in S1H Fig in S1 File, and there was no significant Spearman's correlation between them. This is likely because the mean BMI of our cohort was normal (23.4 kg/m^2^) and the hand-assisted technique indeed helped manage unexpected bleeding during surgery.

The correlation coefficient of numberRA with complications were very small. Ten patients had 3 or 4 renal arteries and none of them developed any complications. Thirty-two patients had 2 renal arteries yet 5 of them (15.6%) developed complications. Eighty-six cases had 1 renal artery, of whom 7 (8.1%) developed complications.

The correlation coefficient of areaPNF with complications was relatively high (0.138) compared with other factors. The distribution of areaPNF was as follows. In an ascending order, the 25th percentile was 5.4 mm^2^, the 50th percentile was 12.0 mm^2^, and the 75th percentile was 21.35 mm^2^. We divided the cohort into 4 subsets with these area as boundaries and counted cases with complications in each subset. The rates of complications were 1 (0.78%) from 0 to the 25th percentile, 3 (2.34%) from the 25th to the 50th percentile, 2 (1.56%) from the 50th to the 75th percentile, and 5 (3.91%) from the 75th to the 100th percentile. Fisher’s exact test did not reveal a significance of complication frequency between groups (p = 0.419).

Therefore, numberRA and areaPNF do not seem to influence the complication rates in our cohort. However, frequency and severity of complications might depend on the nature of the cohort, so we think that the effect of these two factors on complication rates should be validated in the other cohorts.

There are several benefits of this preoperative evaluation. Most notable is its efficacy in reflecting the quantity of PNF in PDA that allows us to distinguish donors with large BMI but little PNF from among the high BMI group (S1D and S1E Figs in S1 File). During development of PDA, this system objectively delineated significant surgical risk factors that posed an operational burden on the surgeon in HALDNx, such as PNF and numberRA. This insight into the technical difficulties allowed stress on operators and possible pitfalls to be anticipated beforehand, making it possible to deploy an appropriate laparoscopist and plan plausible operative strategies. By adopting this method, surgical training could still be accomplished amid the learning curve while avoiding cases that require experience and allowing a gradual increase in operative difficulty. We have therefore continued conducting routine preoperative systematic evaluation for all live kidney donors over the last decade; during that time we have performed more than 1300 HALDNx procedures without conversion to open surgery or blood transfusion.

During the development phase of the preoperative assessment system, the most important process in improving its accuracy was validating the reliability of the assessment system by applying it to future cases in daily clinical practice. An experienced surgeon made the final judgement of the validity of the predictor based on clinical experience. A pivotal question for deploying the predictor in daily practice is whether the predicted level of difficulty matches the surgeon's actual impression of the operation. To achieve this level of accuracy, we repeatedly tested the judgements from the predictor over several years. Our effort was mainly devoted to identifying significant predictive factors useful in improving the prediction. Our goal of identifying such predictive factors led us to discover critical factors in surgical procedures.

Another critical point in creating a good predictor was to select an appropriate dataset for training. The operative time is governed by patient factors and surgeon factors. Exceptional cases should be avoided in the dataset to exclude statistical noises when a dataset is relatively small and limited. For example, a patient with a history of major abdominal surgery should be avoided. The team of surgeons and paramedical staff should be fixed to the extent possible. Additionally, since we found the presence of a second learning curve in addition to the initial one, we selected patients for the training dataset who were on this second learning curve, because a surgeon during this period would likely be a typical operator who would benefit the most from the preoperative assessment system [16].

There are several limitations in this study. Firstly, our study was conducted in a Japanese population whose BMI is relatively lower than that of both white and African populations [19, 21]. The actual predictor we developed using our cohort could be applicable to Japanese or patients with similar physical attributes only. Therefore, the program written in Wolfram Language^®^, Mathematica^®^ that was used to build an NLDR predictor is hereby made available as a supplementary program for our preoperative assessment system to be applied to other cohorts. In our series, only 4 donors (3.1%) had BMI greater than 30. It is therefore desirable that the same kind of study be conducted on different arms, and we expect that our evaluation method would be valid in other races. If a new cohort comprises different races, a factor of race would be included in the final model if race is a significant predictive factor, which is quite probable.

Secondly, we performed the analysis under the assumption that a donor is a healthy individual without any predisposing abdominal diseases. There were, however, some donors who had had some abdominal disorders, such as a history of upper abdominal surgery or a benign tumor near the kidney. For those cases, it should be kept in mind that additional time might be needed in dissecting those diseased areas.

Thirdly, renal veins were not considered in this study. If the patient was a woman who had experienced multiple deliveries, she might present with highly developed gonadal veins, which could complicate surgical procedures. We analyzed enhanced CT images, but information on the arterial venous phases was insufficient.

Fourthly, since we planned to assess the surgical risks of donors, other risk factors concerning surgeons, co-medical staff, and surgical room were kept constant as much as possible to exclude biases in the background. Therefore, the assessment of risk factors of surgeons or other factors than donors were beyond the scope of the present analysis.

Lastly, hard sticky PNF is sometimes found in male donors in their 50s and 60s, as previously mentioned. Wadström reported that men with compact and hard adipose tissue around the kidney tended to prolong operative time and make the operation more difficult [19]. His report is consistent with our experience; we found it difficult to predict the hardness of PNF by using only abdominal CT images or other laboratory data. We are thus aware that further investigation is needed to develop a method to accurately discern the quality of PNF preoperatively, and that there is also room for improvement in accuracy of discrimination with volumetric measurement of PNF.

In conclusion, this is the first report of an objective evaluation system of live kidney donors to predict the surgical workload in HALDNx and to distinguish between heavy-workload and moderate-workload cases. We have succeeded in calibrating the surgical workload as the discrimination index and achieved an estimated total hit rate of 93.8% in the dichotomous discrimination of operative difficulty. The most significant determinant of workload was the quantity of PNF along with numberRA. Donors with metabolic syndrome who have central apple-shaped adiposity should be considered high-risk cases for HALDNx. Based on the excellent results achieved in this series, we believe that further careful study of this method is warranted not only for HALDNx, but also for other similar abdominal surgeries.

## Methods

### Patients

From February 2001 to April 2005, 187 consecutive HALDNx procedures were carried out at the Department of Surgery III of Tokyo Women’s Medical University in Japan. No exclusion criteria for HALDNx were considered. Because none of the cases were converted to open surgery, these cases made up the primary cohort. Of them, 128 cases of left HALDNx performed by a single operator relatively early in his training phase were included in this study, excluding the first 16 cases to account for learning curve, 6 right HALDNx, 15 cases performed by another operator, and 22 cases with no or inadequate data of abdominal CT scan.

This study was approved by the Institutional Review Board of the relevant medical institution (Tokyo Women’s Medical University: approval number 4834). The patients received explanations based on the Helsinki Declaration before providing their written informed consent, and all methods were performed in accordance with relevant guidelines and regulations.

### Clinical variables

We collected the operative time of these HALDNx cases coupled with the following 16 clinical variables from each donor. Donor sex, age, Bw (kg), and Ht (cm) were gathered from the inpatient medical record. NumberRA of the procured kidney and operative time (min), defined as time elapsed from skin incision to skin closure excluding waiting time until the recipient is prepared to be revascularized, were collected from the operative records. Laboratory data of donors’ serum were obtained through a routine early-morning medical check immediately after hospitalization. These data included TP, Alb, TG, and TC. The following 7 variables were measured on the enhanced cross-sectional abdominal CT image at the level of the maximum cross-sectional area of the left kidney: maximum and median distances medially between the kidney and the psoas or quadratus lumborum muscle, median distance laterally between the kidney and the abdominal wall or viscera, area and mean CT value of PNF enclosed by the psoas muscle, the quadratus lumborum muscle, the abdominal wall and viscera, thickness of abdominal wall subcutaneous fat at the midline, and area of subcutaneous fat in the entire abdominal wall (S1A Fig in S1 File).

We performed univariate and multivariate linear regression analysis with operative time as a dependent variable and the 16 clinical variables as independent variables to discern the linear correlation between each variable and operative time.

### Discriminant analysis

Operative time was taken as a numerical measure of surgical workload. Since we planned to assess the surgical difficulty that are evaluated by the surgeon's qualitative impression, we divided the distribution of operative time into 2 groups with the 84.1th percentile (= 210 min) as the boundary between difficult cases and easy ones, because under the standard normal distribution, the mean + 1 SD is $\frac{1}{\sqrt{2e\pi }}$ and the area under the standard normal distribution curve from—∞ to $\frac{1}{\sqrt{2e\pi }}$ is about 0.841. We defined cases with operative time below this boundary as the moderate-workload group G_M_ (n = 107), with the other cases being the heavy-workload group G_H_ (n = 21). For simplicity, we designate G_M_ as easy cases and G_H_ as difficult cases.

We chose one of the best standard parametric dichotomous discriminant rules, NLDR, as a preoperative evaluation rule in PDA, using it to allocate donors to either G_M_ or G_H_ according to clinical variables [27]. From the donor’s variables in our cohort, NLDR returned a coefficient ranging from roughly -7 to 3, as shown in Fig 1. We thus designated it as a discrimination index for the prediction of surgical workload. If it was positive, the case belonged to G_H_ and if negative to G_M_.

The dichotomous PDA yields a 2 × 2 table, shown as Table 6. From this table, 5 statistical rates are obtained, presented in Table 7. We called the positive predictive value the HREC and the negative predictive value the HRDC [28, 29].

**Table 6:** Discriminant analysis.

	Difficult case	Easy case	Total
Predicted difficult	A	B	A + B
Predicted easy	C	D	C + D
Total	A + C	B + D	A + B + C + D

# The operative time threshold between difficult and easy cases was 210 min.

**Table 7:** Definitions in predictive discriminant analysis.

Technical term	Formula
Positive predictive value (PPV)	A/(A + B)
Negative predictive value (NPV)	D/(C + D)
Sensitivity	A/(A + C)
Specificity	D/(B + D)
Total hit rate (accuracy)	(A + D)/(A + B + C + D)

# All values of formulae are expressed in percentage in this manuscript.

# Positive predictive value is to be rephrased as hit rate of easy cases (HREC) and negative predictive value as hit rate of difficult cases (HRDC).

There are 2 kinds of hit rates in PDA: one is the "apparent hit rate" and the other is the "true hit rate". The apparent rate can be obtained by applying the discriminant rule to the original (or training) data from which the PDA rule was constructed. The true rate can be determined by applying the rule to newly obtained (or future) data from another cohort. Efron proposed several statistical methods to estimate the true rate through computer simulation using the training data, from among which we chose the conventional cross-validation and the .632 estimator as the best performer [29].

We tried 4 different subsets of variables, namely, Sets A, B, C, and D, as arguments for the NLDR to improve the accuracy of the PDA. We then calculated both apparent and true hit rates for each subset. At first, we used only four parameters of (sex, age, Bw, and Ht) to represent the body frame of donors. Then we added information about metabolites (TP, Alb, TG, and TC) to Set A to realize Set B. We further incorporated perinephric anatomical data obtained from CT images into Set B to realize Set C. Finally, we t added data on fat tissue in the abdominal wall to Set C to realize Set D (Tables 3, 4 and 5).

Theoretically, there are 65,535 (= 2^16^–1) possible non-empty subsets of 16 clinical variables. The NLDR was applied to all of those subsets to discover the subsets with the highest total hit rate. After selecting subsets with a high total hit rate using cross-validation, we applied the .632 estimator to them to find the one with the highest estimated total hit rate. This subset was defined as Set E.

### Adequacy assessment of threshold between easy cases and difficult cases in PDA

To distinguish easy and difficult cases in HALDNx, we chose operative time as an objective measure of surgical workload and set a threshold between 2 cases at the 84.1th percentile of operative times (ascending order). To assess the adequacy of this boundary, we performed post hoc analysis to see changes in hit rates with using other thresholds. S1I Fig in S1 File shows changes of total hit rate for threshold values from the 2nd to 98th percentile using Set E. S1J Fig in S1 File shows HREC and HRDC under the same conditions and S1K Fig in S1 File shows changes in sensitivities and specificities for difficult cases. As are shown by these figures, the 84.1 percentile is within a range of threshold that allows relatively high statistical indices in discriminant analysis.

### Relations of discrimination indexes with operative time and surgical complications

We performed additional analyses to delineate risk factors associated with operative workload in terms of operative time by determining the Pearson's correlation coefficient of the operative time with each clinical variable. We also compared the predictive factors and surgical complications between 9 patients whose operative time was 240 min or more and 11 patients whose time was 100 min or less to determine the factors that decided these two different outcomes. The discrimination index was also compared to determine whether it reflected such a difference.

We also reviewed surgical complications in the 128 donors and categorized them according to the modified Clavien classification system for laparoscopic donor nephrectomy [17]. The corresponding relations between the discrimination indexes and modified Clavien classification system were plotted case by case and the correlations between them were compared using Spearman's rank-order correlation. The Pearson's correlation coefficients of surgical complications with each predictive variable were also calculated to determine which factors were correlated with surgical complications.

### NLDR

The NLDR is as follows [30]. Supposing that there are 2 groups ***G***₀ and ***G***₁ with ***n***₀ and ***n***₁ as the number of members, respectively, and every member has a feature vector ***x*** {Bw, Ht, sex(1/0), age, …}. Let **μ**₀ and **μ**₁ denote the mean vectors of each group and **Σ** denote the covariance matrix of all the feature vectors from both groups. The discrimination coefficient of a donor with a feature vector ***x*** is given by

$$\Omega \left(x\right)=\text{log}(\pi ₁/\pi_{0})–1/2\{\Delta (x;\mu ₁,\Sigma )–\Delta (x;\mu_{0},\Sigma )\},$$ (1)

where

$$\begin{array}{cc} \Delta (x;\mu_{i},\Sigma )=(x-\mu_{i})´\Sigma^{-1}(x-\mu_{i}) & \left(i=0,1\right) \end{array}$$ (2)

is the Mahalanobis distance between ***x*** and **μ**_*i*_ with respect to **Σ** and the prior probability **π**_*i*_ for each group is defined by

$$\begin{array}{cc} \pi_{i}=n_{i}/(n_{0}+n₁) & \left(i=0,1\right). \end{array}$$ (3)

Then, the optimized rule formed by **μ**_*i*_, **Σ**, and **π**_*i*_ assigns ***x*** to ***G***₁ if ***Ω***(***x***) is positive and to ***G***₀ if negative. We called the value ***Ω***(***x***) the discrimination index. This index is a continuous variable and the assessment system provides not only qualitative assessment, difficult or not, with a sign of the index, but also quantitative evaluation of the degree of difficulty with the absolute value of the index. At the end of the RunSample.pdf file on the GitHub repository (as shown in the data and program availability section), there are sample outputs made by the NLDR model, where the discrimination index is shown as a red line in a rectangle graphics.

### Statistical analysis

Statistical analysis was performed using Wolfram Language^®^, Mathematica^®^ version 11.3 (Wolfram Research Inc., Champaign, IL) and SPSS version 11.0.1 (SPSS Inc., Chicago, IL). Numerical values are expressed as mean ± SD (range). All rates were expressed in percentages. Continuous values were compared using the Mann-Whitney U test and discrete values were compared by the Fisher's exact test. Risk factors were assessed by linear regression. Correlations were compared using either Pearson's correlation coefficient or Spearman's rank correlation coefficient. A *p*-value of less than 0.05 was considered statistically significant.

### Data and program availability and how to use them

The dataset analyzed and the program developed along with supplementary texts, figures and tables are available on GutHub as supplementary files (https://github.com/kiwindow/assessworkload).

The program, which is written in Mathematica^®^ (Wolfram Research Inc., Champaign, IL), is uploaded as PDAprogram.nb.zip. The dataset is saved as haldata.xlsx and haldata.pdf. Usage instructions are provided in ReadMeFirst.nb.zip and a demo is available in RunSample.nb.zip and RunSample.pdf.

## Supporting information

S1 File (PDF)

S2 File (DOCX)

S1 Text (PDF)

S2 Text (PDF)

S3 Text (PDF)

## References

[1] Clayman RV, Kavoussi LR, Soper NJ, Dierks SM, Meretyk S, Darcy MD, et al Laparoscopic nephrectomy: initial case report. J Urol. 1991;146: 278–282. 10.1016/s0022-5347(17)37770-4 1830346

[2] Ratner LE, Ciseck LJ, Moore RG, Cigarroa FG, Kaufman HS, Kavoussi LR. Laparoscopic live donor nephrectomy. Transplantation. 1995;60: 1047–1049. 7491680

[3] Wolf JS Jr., Tchetgen MB, Merion RM. Hand-assisted laparoscopic live donor nephrectomy. Urology. 1998;52: 885–887. 10.1016/s0090-4295(98)00389-6 9801121

[4] Wolf JS Jr., Moon TD, Nakada SY. Hand assisted laparoscopic nephrectomy: comparison to standard laparoscopic nephrectomy. J Urol. 1998;160: 22–27. 9628597

[5] Ratner LE, Hiller J, Sroka M, Weber R, Sikorsky I, Montgomery RA, et al Laparoscopic live donor nephrectomy removes disincentives to live donation. Transplant Proc. 1997;29: 3402–3403. 10.1016/s0041-1345(97)00955-x 9414765

[6] Ratner LE, Montgomery RA, Kavoussi LR. Laparoscopic live donor nephrectomy: the four year Johns Hopkins University experience. Nephrol Dial Transplant. 1999;14: 2090–2093. 10.1093/ndt/14.9.2090 10489214

[7] Philosophe B, Kuo PC, Schweitzer EJ, Farnery AC, Lim JW, Johnson LB, et al Laparoscopic versus open donor nephrectomy: comparing ureteral complications in the recipients and improving the laparoscopic technique. Transplantation. 1999;68: 497–502. 10.1097/00007890-199908270-00009 10480406

[8] Schweitzer EJ, Wilson J, Jacobs S, Machan CH, Philosophe B, Farnery A, et al Increased rates of donation with laparoscopic donor nephrectomy. Ann Surg. 2000;232: 392–400. 10.1097/00000658-200009000-00011 10973389 PMC1421152

[9] Ratner LE, Kavoussi LR, Sroka M, Hiller J, Weber R, Schulam PG, et al Laparoscopic assisted live donor nephrectomy–a comparison with the open approach. Transplantation. 1997;63: 229–233. 10.1097/00007890-199701270-00009 9020322

[10] Wolf JS Jr., Marcovich R, Merion RM, Konnak JW. Prospective, case matched comparison of hand assisted laparoscopic and open surgical live donor nephrectomy. J Urol. 2000;163: 1650–1653. 10799153

[11] Wolf JS Jr., Merion RM, Leichtman AB, Campbell DA, Magee JC, Punch JD, et al Randomized controlled trial of hand-assisted laparoscopic versus open surgical live donor nephrectomy. Transplantation. 2001;72: 284–290. 10.1097/00007890-200107270-00021 11477354

[12] Flowers JL, Jacobs S, Cho E, Morton A, Rosenberger WF, Evans D, et al Comparison of open and laparoscopic live donor nephrectomy. Ann Surg. 1997;22: 483–489. 10.1097/00000658-199710000-00009 PMC1191065 9351716

[13] Gaston KE, Moore DT, Pruthi RS. Hand-assisted laparoscopic nephrectomy: prospective evaluation of the learning curve. J Urol. 2004;171: 63–67. 10.1097/01.ju.0000099400.50350.9b 14665845

[14] Nakajima I, Tojimbara T, Sato S, Kawase T, Fuchinoue S, Teraoka S. Hand-assisted laparoscopic live donor nephrectomy: a single center experience in Japan. Transplant Proc. 2003;35: 43–44. 10.1016/s0041-1345(02)03932-5 12591299

[15] Nakajima I, Tojimbara T, Sato S, Kai K, Kawase T, Nakamura M, et al Hand-assisted laparoscopic live donor nephrectomy: report of 100 cases. Transplant Proc. 2004;36: 1898–1900. 10.1016/j.transproceed.2004.07.045 15518690

[16] Nakajima I, Iwadoh K, Koyama I, Tojimbara T, Teraoka S, Fuchinoue S. Nine-yr experience of 700 hand-assisted laparoscopic donor nephrectomies in Japan. Clin Transplant. 2012;26: 797–807. 10.1111/j.1399-0012.2012.01617.x 22449123

[17] Kocak B, Koffron A, Baker T, Salvalaggio P, Kaufman D, Fryer J, et al Proposed classification of complications after live donor nephrectomy. Urology. 2006;67(5): 927–031 10.1016/j.urology.2005.11.023 16698353

[18] Leventhal JR, Kocak B, Salvalaggio PR, Kofron AJ, Baker TB, Kaufman DB, et al Laparoscopic donor nephrectomy 1997 to 2003: Lessons learned with 500 cases at a single institution. Surgery. 2004;136: 881–890. 10.1016/j.surg.2004.06.025 15467675

[19] Wadström J. Hand-assisted retroperitoneoscopic live donor nephrectomy: experience from the first 75 consecutive cases. Transplantation. 2005;80: 1060–1066. 10.1097/01.tp.0000176477.81591.6f 16278586

[20] Su LM, Ratner LE, Montgomery RA, Jarrett TW, Trock BJ, Sinkov V, et al Laparoscopic live donor nephrectomy. Ann Surg. 2004;240: 358–363. 10.1097/01.sla.0000133351.98195.1c 15273562 PMC1356414

[21] Neipp M, Jackobs S, Becker T, zu Vilsendorf AM, Winny M, Lueck R, et al Living donor nephrectomy: flank incision versus anterior vertical mini-incision. Transplantation. 2004;78: 1356–1361. 10.1097/01.tp.0000140975.96729.a7 15548975

[22] Anderson K, Lindler T, Lamberton G, Baron P, Ojogho O, Baldwin D. Laparoscopic donor nephrectomy: effect of perirenal fat upon donor operative time. J Endourol. 2008;22: 2269–2274. 10.1089/end.2008.9725 18831674

[23] Ratner LE, Smith P, Montgomery RA, Mandal AK, Fabrizio M, Kavoussi LR. Laparoscopic live donor nephrectomy: pre-operative assessment of technical difficulty. Clin Transplant. 2000;14: 427–432. 10.1034/j.1399-0012.2000.14041202.x 10946783

[24] Nakajima I, Tojimbara T, Iwadoh K, Nanmoku K, Kai K, Koyama I, et al. The safety of live donors in renal transplantation. Jpn J Transplant. 2004;39 supplemetary: 158.

[25] Iwadoh K, Nakajima I, Tojimbara T, Kai K, Nanmoku K, Koyama I, et al Preoperative assessment system of live donor nephrectomy in kidney transplantation. Jpn J Transplant. 2005;40: 392.

[26] Iwadoh K, Nakajima I, Tojimbara T, Kato Y, Kai K, Nanmoku K, et al Predictive factors of operative workload in hand-assisted laparoscopic donor nephrectomy. Jpn J Transplant. 2006;41: 296.

[27] McLachlan GJ. Discriminant analysis and statistical pattern recognition. Hoboken (NJ): Wiley-Interscience; 1992.

[28] Huberty CJ. Applied discriminant analysis. Hoboken (NJ): Wiley-Interscience; 1994.

[29] Efron B. Estimating the error rate of a prediction rule: improvement on cross-validation. J Am Stat Assoc. 1983;78: 316–331.

[30] Armitage P, Berry G, Matthews JNS. Statistical methods in medical research. 4th ed Hoboken (NJ): Blackwell Publishing; 2002.

