# Supplementary material for: Preoperative assessment system for hand-assisted laparoscopic donor nephrectomy by discriminant analysis
Source: PLoS One. 2020 Apr 28;15(4):e0227546. doi: 10.1371/journal.pone.0227546 (PMC7188199; doi:10.1371/journal.pone.0227546)
Supplement: S1 File — (PDF) [file pone.0227546.s001.pdf]

S1 Fig. (A) Notation of parameters in an abdominal CT image.

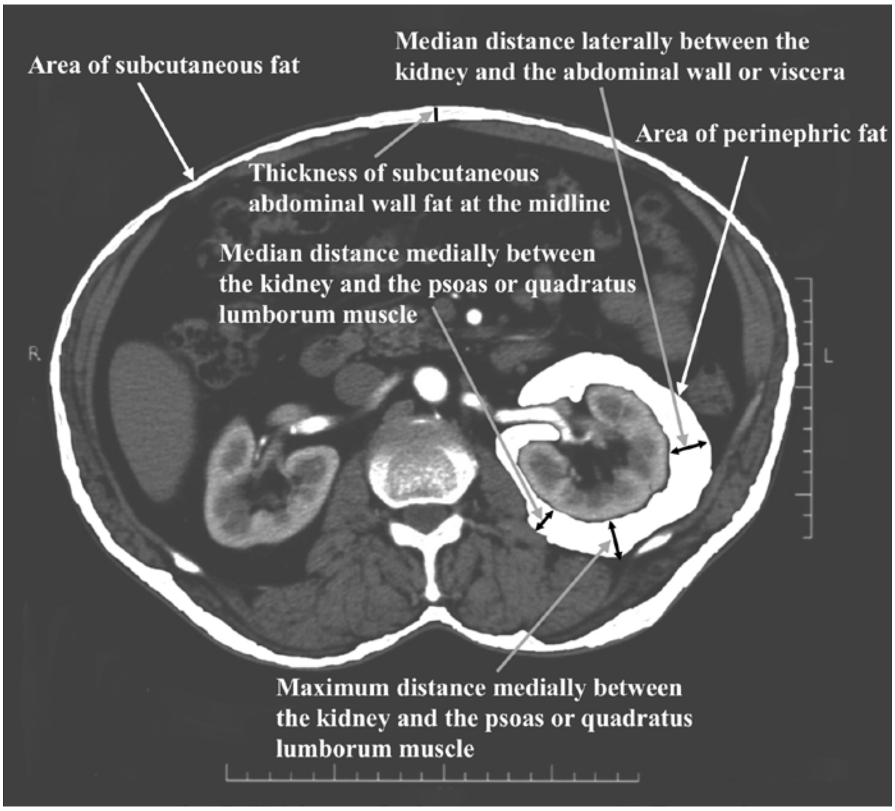

Median distance (fat thickness) medially between the kidney and the psoas or quadratus lumborum muscle is called median thickness of medial perinephric fat (= medthickMPF).

Maximum distance (fat thickness) medially between the kidney and the psoas or quadratus lumborum muscle is called maximum thickness of lateral perinephric fat (= maxthickMPF).

Median distance (fat thickness) laterally between the kidney and the abdominal wall or viscera is called median thickness of lateral perinephric fat (= medthickLPF).

Area of perinephric fat = areaPNF

Area of subcutaneous fat = areaSCF

S1 Fig. (B) Abdominal cross-sectional CT scan image of a 57-year-old male donor with discrimination index of 2.14 and operative time of 270 min.

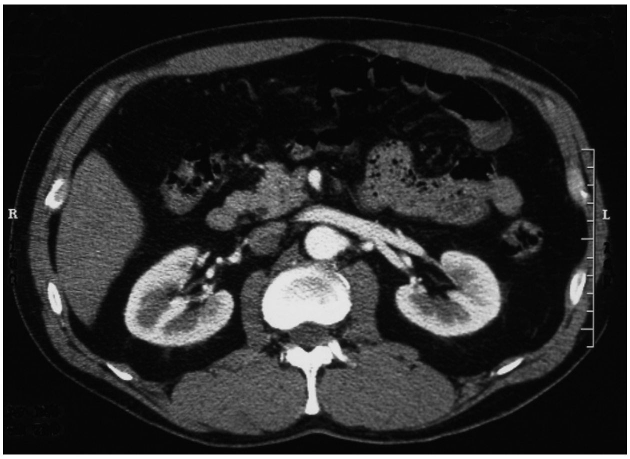

S1 Fig. (C) Abdominal cross-sectional CT scan image of a 61-year-old female donor with discrimination index of -6.77 and operative time of 120 min.

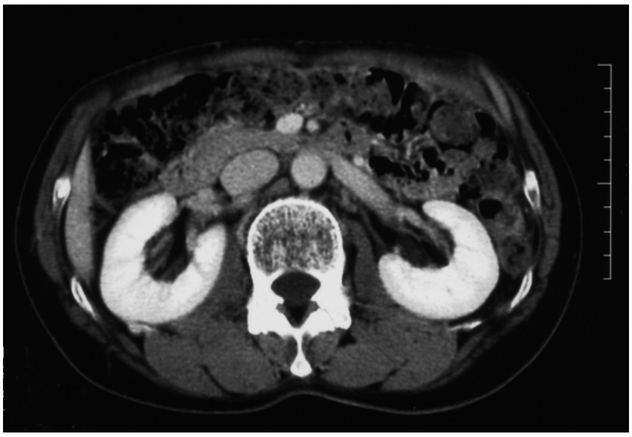

S1 Fig. (D) Abdominal cross-sectional CT scan image of a 47-year-old female donor who had pear-shaped adiposity with discrimination index of -2.30 and operative time of 120 min.

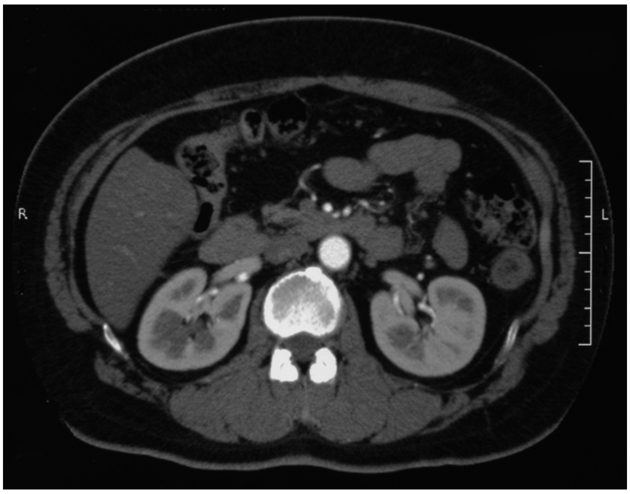

S1 Fig. (E) Abdominal cross-sectional CT scan image of a 61-year-old male donor who had apple-shaped adiposity with discrimination index of 1.93 and operative time of 360 min.

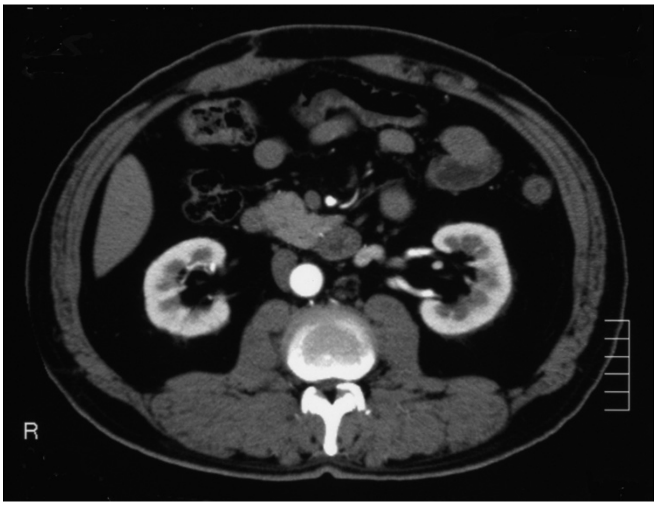

S1 Fig. (F) Pearson's correlation coefficients of operative time with each predictive factor

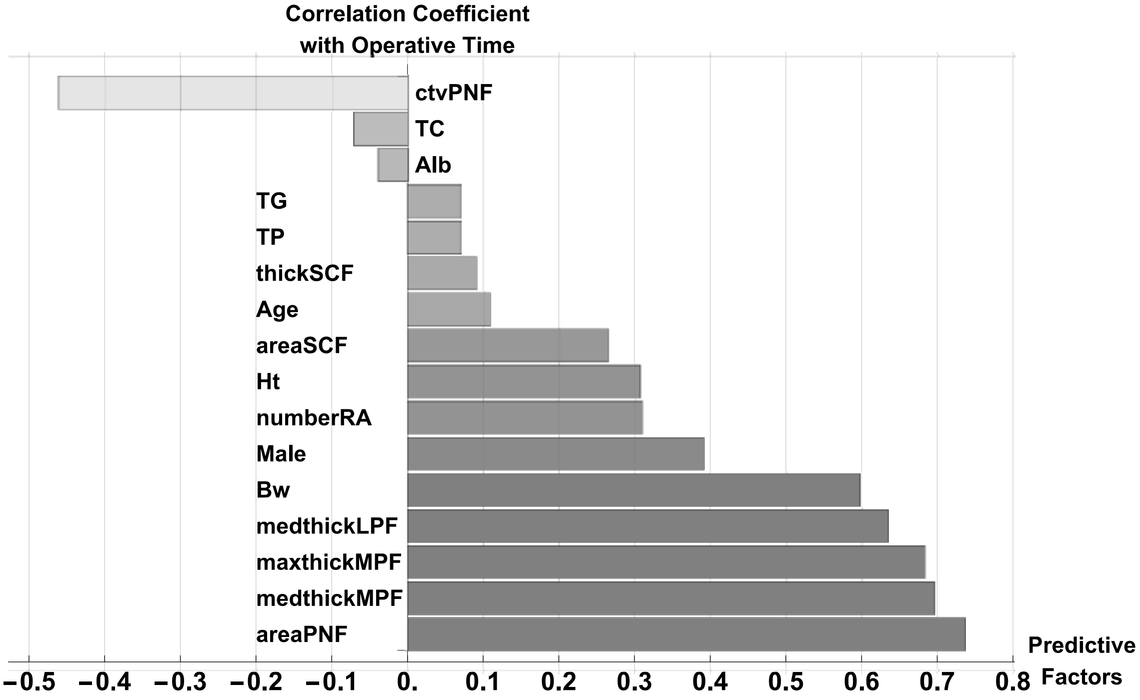

The horizontal axis indicates a value of correlation coefficient. The right-hand side means positive correlation, and the other negative. The vertical axis indicates each predictive variable.

numberRA = number of renal arteries of the graft, maxthickMPF = maximum thickness of medial perinephric fat, medthickMPF = median thickness of medial perinephric fat, medthickLPF = median thickness of lateral perinephric fat, areaPNF = area of perinephric fat, ctvPNF = CT value of perinephric fat density, thickSCF = thickness of subcutaneous abdominal fat at the midline, areaSCF = area of subcutaneous fat.

S1 Fig. (G) Pearson's correlation coefficients of complication event with predictive factors

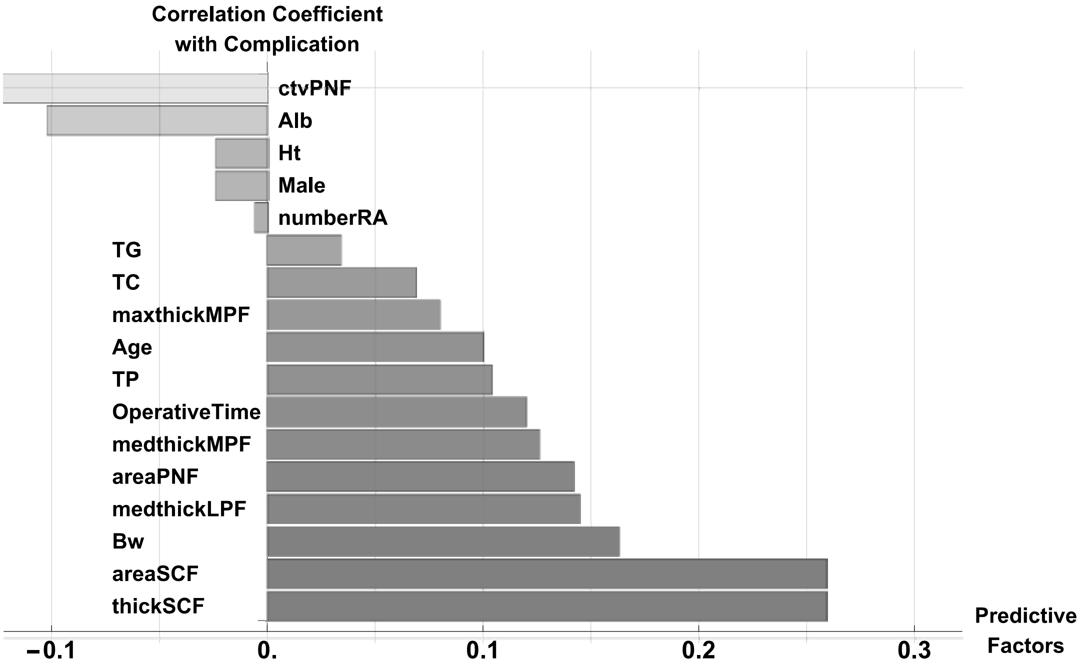

The horizontal axis indicates a value of correlation coefficient. The right-hand side means positive correlation, and the other negative. The vertical axis indicates each predictive variable.

numberRA = number of renal arteries of the graft, maxthickMPF = maximum thickness of medial perinephric fat, medthickMPF = median thickness of medial perinephric fat, medthickLPF = median thickness of lateral perinephric fat, areaPNF = area of perinephric fat, ctvPNF = CT value of perinephric fat density, thickSCF = thickness of subcutaneous abdominal fat at the midline, areaSCF = area of subcutaneous fat.

S1 Fig. (H) Corresponding relation between discrimination index and modified Clavien Grading

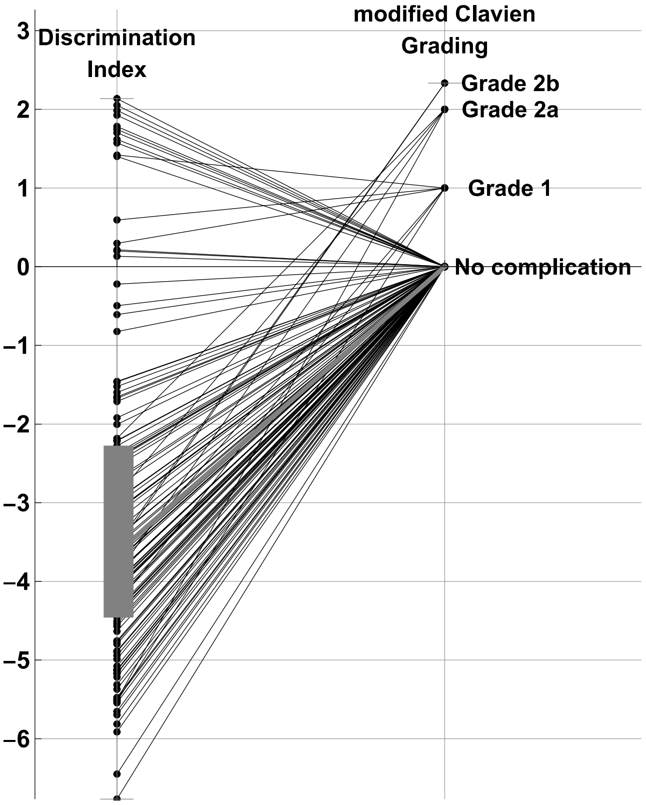

The dots on the left vertical line indicate discrimination index of each donor. The bottom side of the gray rectangle along the line represents 25 percentile point of the indexes in the ascending order. Its top side represents 75 percentile. The dots on the right vertical line indicate grading of modified Clavien classification system of surgical complications: grade0 as 0, grad1 as 1, grade 2a as 2 and grade2b as 2.33333. There were no case with grade 2c or more. The Spearman's rank correlation coefficient between the discrimination index and the Clavien grading was not statistically significant ($p = 0.785$).

S1 Fig. (I) Changes in total hit rate in PDA according to threshold percentile of operative time between easy and difficult cases.

Total hit rate (%)

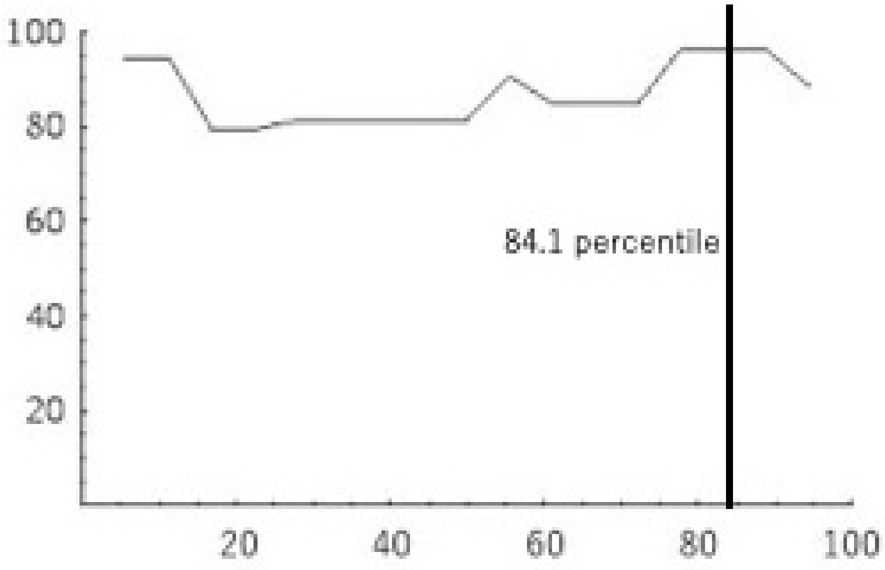

Percentile

Horizontal axis indicates the threshold percentile of operative time between easy and difficult cases. Vertical axis indicates the total hit rate of the preoperative assessment system for HALDNx. Mean hit rate for all thresholds was 86.79 ± 6.54%; median hit rate was 84.91%. PDA: predictive discriminant analysis.

S1 Fig. (J) Changes in HREC and HRDC in PDA according to threshold percentile of operative time between easy and difficult cases.

Hit rate (%)

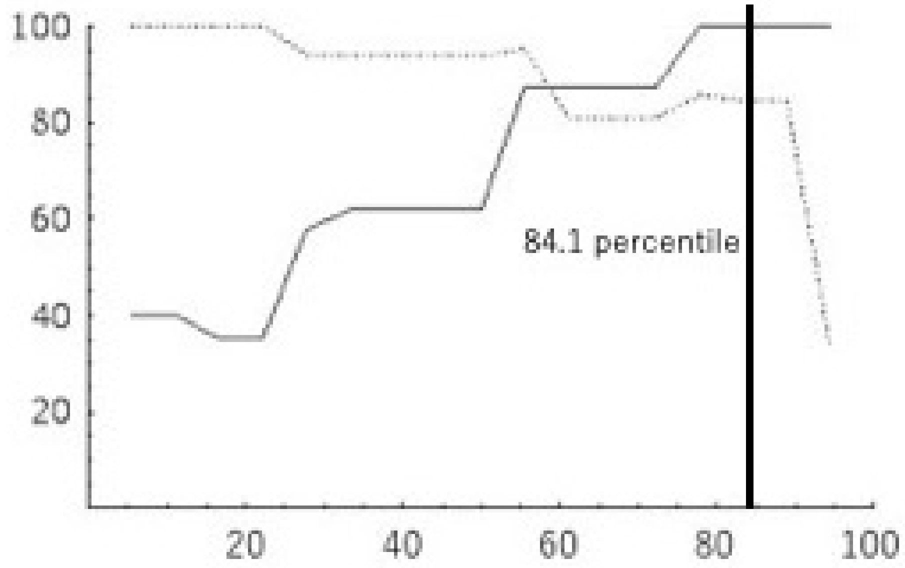

Percentile

Horizontal axis indicates the threshold percentile of operative time between easy and difficult cases. Vertical axis indicates the hit rate of preoperative assessment system for HALDNx. The black line represents the hit rate of easy cases, and the dashed line represents the hit rate for difficult cases. Mean hit rate of difficult cases for all thresholds was 70.95 ± 24.27%; median hit rate was 61.90%. Mean hit rate of easy cases was 87.97 ± 15.74%; median hit rate was 93.75%. PDA: predictive discriminant analysis.

S1 Fig. (K) Changes in sensitivity and specificity of difficult cases in PDA according to threshold percentile of operative time between easy and difficult cases.

Sensitivity/Specificity

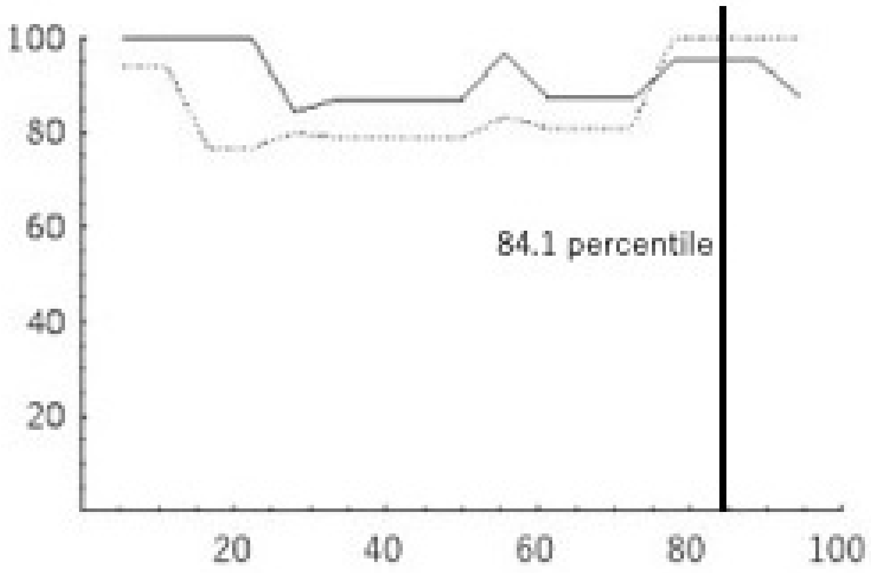

Percentile

Horizontal axis indicates the threshold percentile of operative time between easy and difficult cases. Vertical axis indicates the hit rate of preoperative assessment system for HALDNx. The black line represents sensitivity and the dashed line represents specificity for easy cases. Mean sensitivity for easy cases for all thresholds was 92.00 ± 5.88%; median sensitivity was 88.00%. Mean specificity for easy cases was 86.08 ± 9.39%; median specificity was 80.95%. PDA: predictive discriminant analysis.
