# Supplementary material for: Preoperative assessment system for hand-assisted laparoscopic donor nephrectomy by discriminant analysis
Source: PLoS One. 2020 Apr 28;15(4):e0227546. doi: 10.1371/journal.pone.0227546 (PMC7188199; doi:10.1371/journal.pone.0227546)
Supplement: S2 File — (DOCX) [file pone.0227546.s002.docx]

S1 Table. (A) Apparent and estimated true hit rates in the estimation of surgical workload in HALDNx as calculated by different statistical methods

|  | NQDR | NLDR | 3-NNR | 5-NNR | FF | RBF | Logistic | Linear | Average |
| --- | --- | --- | --- | --- | --- | --- | --- | --- | --- |
| Apparent Hit Rate | 92.2% | 97.4% | 92.1% | 88.9% | 99.4% | 95.3% | 92.4% | 86.6% | 93.0±4.2% |
| Cross-Validation | 86.1% | 94.8% | 87.6% | 88.8% | 89.6% | 91.9% | 88.9% | 86.6% | 89.3±2.9% |
| Simple Bootstrap | 83.8% | 94.4% | 84.6% | 79.6% | 96.0% | 92.5% | 90.4% | 84.8% | 88.3±5.9% |
| Jack Knife | 86.3% | 94.9% | 87.7% | 88.8% | 87.6% | 91.5% | 89.4% | 84.0% | 88.8±3.3% |
| Randomized Bootstrap | 89.9% | 96.9% | 88.2% | 86.3% | 95.7% | 91.5% | 89.8% | 85.1% | 90.4±4.2% |
| .632 Estimator | 88.6% | 95.6% | 89.4% | 88.1% | 92.6% | 88.2% | 90.9% | 88.2% | 90.2±2.7% |
| Average Hit Rate | 86.9±2.4% | 95.3±1.0% | 87.5±1.8% | 86.3±3.9% | 92.3±3.8% | 91.1±1.8% | 89.9±0.8% | 85.7±1.7% |  |

Results in this table were calculated using a different cohort from the one used in this study. Apparent hit rate is the discrimination rate of easy cases in HALDNx used for the training data, which used different discriminators constructed from the training data itself.

Other hit rates were estimated for future data by several estimation methods. Details of this kind of comparison between discriminators and estimators are found in Reference 30 (Efron B, J Am Stat Assoc. 1983;78: 316-331). Average hit rates are shown as the mean ± standard deviation.

HALDNx, hand-assisted laparoscopic donor nephrectomy; NQDR, normal-based quadratic discriminant rule; NLDR, normal-based linear discriminant rule; 3-NNR, 3-nearest neighborhood rule; 5-NNR, 5-nearest neighborhood rule; FF, feedforward neural network; RBF, radial basis function neural network; Logistic, logistic regression; Linear, linear regression; Cross Validation, cross-validation method; Simple Bootstrap, simple randomized bootstrap method; Jack Knife, Jack Knife estimate of bias; .632 Estimator, .632(ε - err).

S1 Table. (B) Comparison of predictive factors, discrimination indexes, and complications between 9 cases with the longest operative times and 11 cases with the shortest operative times

Cases with an operative time of 240 min or more comprise the long operative time group and those with an operative time of 100 min or less comprise the short operative time group.

numberRA, number of renal arteries of the graft; maxthickMPF, maximum thickness of medial perinephric fat; medthickMPF, median thickness of medial perinephric fat; medthickLPF, median thickness of lateral perinephric fat; areaPNF, area of perinephric fat; ctvPNF, CT value of perinephric fat density; thickSCF, thickness of subcutaneous abdominal fat at the midline; areaSCF, area of subcutaneous fat.

S1 Table. (C) Surgical complications in 128 HALDNx according to modified Clavien classification system

This table shows all complications developed in 128 HALDNx with predictive factors, discrimination index, and modified Clavien classification.

numberRA, number of renal arteries of the graft; maxthickMPF, maximum thickness of medial perinephric fat; medthickMPF, median thickness of medial perinephric fat; medthickLPF, median thickness of lateral perinephric fat; areaPNF, area of perinephric fat; ctvPNF, CT value of perinephric fat density; thickSCF, thickness of subcutaneous abdominal fat at the midline; areaSCF, area of subcutaneous fat.
