# Supplementary material for: Preoperative assessment system for hand-assisted laparoscopic donor nephrectomy by discriminant analysis
Source: PLoS One. 2020 Apr 28;15(4):e0227546. doi: 10.1371/journal.pone.0227546 (PMC7188199; doi:10.1371/journal.pone.0227546)
Supplement: S1 Text — (PDF) [file pone.0227546.s003.pdf]

395 kinds of variable combinations with 2 x 2 table of {{107, 5},{ 0, 16}} and 95.5% of total hit rate

■output form = { { variable1, variable2, variable3, ... }, { { N of true easy, N of false easy, N of false difficult, N of true difficult},
{{hit rate of easy cases, hit rate of difficult cases}, {sensitivity of easy cases, specificity of easy cases}, total hit rate}

```
In[]:= best395 =
  {{{"Dage", "artery", "Kiddismax", "AFAaround", "Subcutmax", "TP01", "TC01"},
    {{107, 5, 0, 16}, {{95.53571428571429`, 100.`}, {100.`, 76.19047619047619`},
      0.9609375`}}}, {{"artery", "Kiddismax", "Kiddis内",
     "RFAaround", "AFAaround", "TP01", "TC01"}, {{107, 5, 0, 16},
     {{95.53571428571429`, 100.`}, {100.`, 76.19047619047619`}, 0.9609375`}}},
   {{"Dbw", "Dage", "artery", "Kiddismax", "RCTaround",
     "AFAaround", "TP01", "TC01"}, {{107, 5, 0, 16},
     {{95.53571428571429`, 100.`}, {100.`, 76.19047619047619`}, 0.9609375`}}},
   {{"Dbw", "Dage", "artery", "Kiddismax", "AFAaround", "TP01", "TG01", "TC01"},
    {{107, 5, 0, 16}, {{95.53571428571429`, 100.`},
      {100.`, 76.19047619047619`}, 0.9609375`}}},
   {{"Dbw", "artery", "Kiddismax", "Kiddis外", "RCTaround",
     "AFAaround", "TP01", "TC01"}, {{107, 5, 0, 16},
     {{95.53571428571429`, 100.`}, {100.`, 76.19047619047619`}, 0.9609375`}}},
   {{"Dage", "artery", "Kiddismax", "Kiddis内", "RFAaround",
     "RCTaround", "AFAaround", "TC01"}, {{107, 5, 0, 16},
     {{95.53571428571429`, 100.`}, {100.`, 76.19047619047619`}, 0.9609375`}}},
   {{"Dage", "artery", "Kiddismax", "Kiddis内", "RFAaround",
     "RCTaround", "Subcutmax", "TP01"}, {{107, 5, 0, 16},
     {{95.53571428571429`, 100.`}, {100.`, 76.19047619047619`}, 0.9609375`}}},
   {{"Dage", "artery", "Kiddismax", "Kiddis外", "AFAaround",
     "Subcutmax", "TP01", "TC01"}, {{107, 5, 0, 16},
     {{95.53571428571429`, 100.`}, {100.`, 76.19047619047619`}, 0.9609375`}}},
   {{"Dage", "artery", "Kiddismax", "RCTaround", "AFAaround",
     "Subcutmax", "TP01", "TC01"}, {{107, 5, 0, 16},
     {{95.53571428571429`, 100.`}, {100.`, 76.19047619047619`}, 0.9609375`}}},
   {{"Dage", "artery", "Kiddismax", "RCTaround", "AFAaround",
     "Subcutmax", "Alb01", "TC01"}, {{107, 5, 0, 16},
     {{95.53571428571429`, 100.`}, {100.`, 76.19047619047619`}, 0.9609375`}}},
   {{"Dage", "artery", "Kiddismax", "RCTaround", "AFAaround",
     "TP01", "Alb01", "TC01"}, {{107, 5, 0, 16},
     {{95.53571428571429`, 100.`}, {100.`, 76.19047619047619`}, 0.9609375`}}},
   {{"Dage", "artery", "Kiddismax", "RCTaround", "AFAaround",
     "Alb01", "TG01", "TC01"}, {{107, 5, 0, 16},
     {{95.53571428571429`, 100.`}, {100.`, 76.19047619047619`}, 0.9609375`}}},
   {{"Dage", "artery", "Kiddismax", "AFAaround", "Subcutmax",
```

```
  "TP01", "Alb01", "TC01"}, {{107, 5, 0, 16},
  {{95.53571428571429`, 100.`}, {100.`, 76.19047619047619`}, 0.9609375`}}},
{{"Dage", "artery", "Kiddismax", "AFAaround", "Subcutmax",
  "TP01", "TG01", "TC01"}, {{107, 5, 0, 16},
  {{95.53571428571429`, 100.`}, {100.`, 76.19047619047619`}, 0.9609375`}}},
{{"artery", "Kiddismax", "Kiddis内", "Kiddis外", "RCTaround",
  "AFAaround", "Subcutmax", "TC01"}, {{107, 5, 0, 16},
  {{95.53571428571429`, 100.`}, {100.`, 76.19047619047619`}, 0.9609375`}}},
{{"artery", "Kiddismax", "Kiddis内", "Kiddis外", "RCTaround",
  "AFAaround", "TG01", "TC01"}, {{107, 5, 0, 16},
  {{95.53571428571429`, 100.`}, {100.`, 76.19047619047619`}, 0.9609375`}}},
{{"artery", "Kiddismax", "Kiddis内", "RFAaround", "RCTaround",
  "AFAaround", "TP01", "TC01"}, {{107, 5, 0, 16},
  {{95.53571428571429`, 100.`}, {100.`, 76.19047619047619`}, 0.9609375`}}},
{{"artery", "Kiddismax", "Kiddis内", "RFAaround", "AFAaround",
  "TP01", "Alb01", "TC01"}, {{107, 5, 0, 16},
  {{95.53571428571429`, 100.`}, {100.`, 76.19047619047619`}, 0.9609375`}}},
{{"artery", "Kiddismax", "Kiddis内", "RCTaround", "AFAaround",
  "Subcutmax", "TG01", "TC01"}, {{107, 5, 0, 16},
  {{95.53571428571429`, 100.`}, {100.`, 76.19047619047619`}, 0.9609375`}}},
{{"artery", "Kiddismax", "Kiddis外", "RCTaround", "AFAaround",
  "Subcutmax", "TP01", "TC01"}, {{107, 5, 0, 16},
  {{95.53571428571429`, 100.`}, {100.`, 76.19047619047619`}, 0.9609375`}}},
{{"artery", "Kiddismax", "Kiddis外", "RCTaround", "AFAaround",
  "TP01", "Alb01", "TC01"}, {{107, 5, 0, 16},
  {{95.53571428571429`, 100.`}, {100.`, 76.19047619047619`}, 0.9609375`}}},
{{"Dht", "Dbw", "Dage", "artery", "Kiddismax", "Kiddis外",
  "AFAaround", "TP01", "TC01"}, {{107, 5, 0, 16},
  {{95.53571428571429`, 100.`}, {100.`, 76.19047619047619`}, 0.9609375`}}},
{{"Dht", "Dbw", "Dage", "artery", "Kiddismax", "RCTaround",
  "AFAaround", "TP01", "TC01"}, {{107, 5, 0, 16},
  {{95.53571428571429`, 100.`}, {100.`, 76.19047619047619`}, 0.9609375`}}},
{{"Dht", "Dbw", "Dage", "artery", "Kiddismax", "AFAaround",
  "TP01", "TG01", "TC01"}, {{107, 5, 0, 16},
  {{95.53571428571429`, 100.`}, {100.`, 76.19047619047619`}, 0.9609375`}}},
{{"Dht", "Dbw", "artery", "Kiddismax", "Kiddis外", "RCTaround",
  "AFAaround", "TP01", "TC01"}, {{107, 5, 0, 16},
  {{95.53571428571429`, 100.`}, {100.`, 76.19047619047619`}, 0.9609375`}}},
{{"Dht", "Dage", "artery", "Kiddismax", "Kiddis内", "RCTaround",
  "AFAaround", "TP01", "TC01"}, {{107, 5, 0, 16},
  {{95.53571428571429`, 100.`}, {100.`, 76.19047619047619`}, 0.9609375`}}},
{{"Dht", "Dage", "artery", "Kiddismax", "Kiddis外", "RCTaround",
  "AFAaround", "TP01", "TC01"}, {{107, 5, 0, 16},
  {{95.53571428571429`, 100.`}, {100.`, 76.19047619047619`}, 0.9609375`}}},
{{"Dht", "Dage", "artery", "Kiddismax", "RCTaround", "AFAaround",
  "Subcutmax", "TP01", "TC01"}, {{107, 5, 0, 16},
```

```
  {{95.53571428571429`, 100.`}, {100.`, 76.19047619047619`}, 0.9609375`}}},
{{"Dht", "Dage", "artery", "Kiddismax", "RCTaround", "AFAaround",
  "TP01", "Alb01", "TC01"}, {{107, 5, 0, 16},
  {{95.53571428571429`, 100.`}, {100.`, 76.19047619047619`}, 0.9609375`}}},
{{"Dht", "Dage", "artery", "Kiddismax", "RCTaround", "AFAaround",
  "Alb01", "TG01", "TC01"}, {{107, 5, 0, 16},
  {{95.53571428571429`, 100.`}, {100.`, 76.19047619047619`}, 0.9609375`}}},
{{"Dht", "artery", "Kiddismax", "Kiddis外", "RCTaround",
  "AFAaround", "Subcutmax", "TP01", "TC01"}, {{107, 5, 0, 16},
  {{95.53571428571429`, 100.`}, {100.`, 76.19047619047619`}, 0.9609375`}}},
{{"Dht", "artery", "Kiddismax", "Kiddis外", "RCTaround",
  "AFAaround", "TP01", "Alb01", "TC01"}, {{107, 5, 0, 16},
  {{95.53571428571429`, 100.`}, {100.`, 76.19047619047619`}, 0.9609375`}}},
{{"Dht", "Kiddismax", "Kiddis内", "RCTaround", "AFAaround",
  "Subcutmax", "TP01", "Alb01", "TC01"}, {{107, 5, 0, 16},
  {{95.53571428571429`, 100.`}, {100.`, 76.19047619047619`}, 0.9609375`}}},
{{"Dbw", "Dage", "artery", "Kiddismax", "Kiddis内", "AFAaround",
  "TP01", "TG01", "TC01"}, {{107, 5, 0, 16},
  {{95.53571428571429`, 100.`}, {100.`, 76.19047619047619`}, 0.9609375`}}},
{{"Dbw", "Dage", "artery", "Kiddismax", "Kiddis外", "RCTaround",
  "AFAaround", "TP01", "TC01"}, {{107, 5, 0, 16},
  {{95.53571428571429`, 100.`}, {100.`, 76.19047619047619`}, 0.9609375`}}},
{{"Dbw", "Dage", "artery", "Kiddismax", "Kiddis外", "AFAaround",
  "TP01", "TG01", "TC01"}, {{107, 5, 0, 16},
  {{95.53571428571429`, 100.`}, {100.`, 76.19047619047619`}, 0.9609375`}}},
{{"Dbw", "Dage", "artery", "Kiddismax", "RCTaround", "AFAaround",
  "TP01", "Alb01", "TC01"}, {{107, 5, 0, 16},
  {{95.53571428571429`, 100.`}, {100.`, 76.19047619047619`}, 0.9609375`}}},
{{"Dbw", "Dage", "artery", "Kiddismax", "RCTaround", "AFAaround",
  "TP01", "TG01", "TC01"}, {{107, 5, 0, 16},
  {{95.53571428571429`, 100.`}, {100.`, 76.19047619047619`}, 0.9609375`}}},
{{"Dbw", "Dage", "artery", "Kiddismax", "RCTaround", "AFAaround",
  "Alb01", "TG01", "TC01"}, {{107, 5, 0, 16},
  {{95.53571428571429`, 100.`}, {100.`, 76.19047619047619`}, 0.9609375`}}},
{{"Dbw", "Dage", "Kiddismax", "Kiddis内", "Kiddis外", "AFAaround",
  "Subcutmax", "TP01", "TC01"}, {{107, 5, 0, 16},
  {{95.53571428571429`, 100.`}, {100.`, 76.19047619047619`}, 0.9609375`}}},
{{"Dbw", "artery", "Kiddismax", "Kiddis外", "RCTaround",
  "AFAaround", "Subcutmax", "TP01", "TC01"}, {{107, 5, 0, 16},
  {{95.53571428571429`, 100.`}, {100.`, 76.19047619047619`}, 0.9609375`}}},
{{"Dbw", "artery", "Kiddismax", "Kiddis外", "RCTaround",
  "AFAaround", "TP01", "Alb01", "TC01"}, {{107, 5, 0, 16},
  {{95.53571428571429`, 100.`}, {100.`, 76.19047619047619`}, 0.9609375`}}},
{{"Dbw", "artery", "Kiddismax", "Kiddis外", "RCTaround",
  "AFAaround", "TP01", "TG01", "TC01"}, {{107, 5, 0, 16},
  {{95.53571428571429`, 100.`}, {100.`, 76.19047619047619`}, 0.9609375`}}},
```

```
{{"Dsex", "Dage", "artery", "Kiddismax", "RCTaround", "AFAaround",
  "TP01", "Alb01", "TC01"}, {{107, 5, 0, 16},
  {{95.53571428571429`, 100.`}, {100.`, 76.19047619047619`}, 0.9609375`}}},
{{"Dage", "artery", "Kiddismax", "Kiddis内", "Kiddis外", "RFAaround",
  "RCTaround", "AFAaround", "TC01"}, {{107, 5, 0, 16},
  {{95.53571428571429`, 100.`}, {100.`, 76.19047619047619`}, 0.9609375`}}},
{{"Dage", "artery", "Kiddismax", "Kiddis内", "Kiddis外", "RFAaround",
  "RCTaround", "Subcutmax", "TP01"}, {{107, 5, 0, 16},
  {{95.53571428571429`, 100.`}, {100.`, 76.19047619047619`}, 0.9609375`}}},
{{"Dage", "artery", "Kiddismax", "Kiddis内", "Kiddis外", "RCTaround",
  "AFAaround", "Subcutmax", "TC01"}, {{107, 5, 0, 16},
  {{95.53571428571429`, 100.`}, {100.`, 76.19047619047619`}, 0.9609375`}}},
{{"Dage", "artery", "Kiddismax", "Kiddis内", "RFAaround", "RCTaround",
  "AFAaround", "Subcutmax", "TC01"}, {{107, 5, 0, 16},
  {{95.53571428571429`, 100.`}, {100.`, 76.19047619047619`}, 0.9609375`}}},
{{"Dage", "artery", "Kiddismax", "Kiddis内", "RFAaround",
  "RCTaround", "AFAaround", "TP01", "TC01"}, {{107, 5, 0, 16},
  {{95.53571428571429`, 100.`}, {100.`, 76.19047619047619`}, 0.9609375`}}},
{{"Dage", "artery", "Kiddismax", "Kiddis内", "RFAaround",
  "RCTaround", "AFAaround", "Alb01", "TC01"}, {{107, 5, 0, 16},
  {{95.53571428571429`, 100.`}, {100.`, 76.19047619047619`}, 0.9609375`}}},
{{"Dage", "artery", "Kiddismax", "Kiddis内", "RFAaround",
  "RCTaround", "AFAaround", "TG01", "TC01"}, {{107, 5, 0, 16},
  {{95.53571428571429`, 100.`}, {100.`, 76.19047619047619`}, 0.9609375`}}},
{{"Dage", "artery", "Kiddismax", "Kiddis内", "RCTaround",
  "AFAaround", "Subcutmax", "TG01", "TC01"}, {{107, 5, 0, 16},
  {{95.53571428571429`, 100.`}, {100.`, 76.19047619047619`}, 0.9609375`}}},
{{"Dage", "artery", "Kiddismax", "Kiddis外", "RCTaround",
  "AFAaround", "Subcutmax", "TP01", "TC01"}, {{107, 5, 0, 16},
  {{95.53571428571429`, 100.`}, {100.`, 76.19047619047619`}, 0.9609375`}}},
{{"Dage", "artery", "Kiddismax", "Kiddis外", "RCTaround",
  "AFAaround", "Subcutmax", "Alb01", "TC01"}, {{107, 5, 0, 16},
  {{95.53571428571429`, 100.`}, {100.`, 76.19047619047619`}, 0.9609375`}}},
{{"Dage", "artery", "Kiddismax", "Kiddis外", "RCTaround",
  "AFAaround", "TP01", "Alb01", "TC01"}, {{107, 5, 0, 16},
  {{95.53571428571429`, 100.`}, {100.`, 76.19047619047619`}, 0.9609375`}}},
{{"Dage", "artery", "Kiddismax", "Kiddis外", "RCTaround",
  "AFAaround", "Alb01", "TG01", "TC01"}, {{107, 5, 0, 16},
  {{95.53571428571429`, 100.`}, {100.`, 76.19047619047619`}, 0.9609375`}}},
{{"Dage", "artery", "Kiddismax", "Kiddis外", "AFAaround",
  "Subcutmax", "TP01", "Alb01", "TC01"}, {{107, 5, 0, 16},
  {{95.53571428571429`, 100.`}, {100.`, 76.19047619047619`}, 0.9609375`}}},
{{"Dage", "artery", "Kiddismax", "Kiddis外", "AFAaround",
  "Subcutmax", "TP01", "TG01", "TC01"}, {{107, 5, 0, 16},
  {{95.53571428571429`, 100.`}, {100.`, 76.19047619047619`}, 0.9609375`}}},
```

```
{{"Dage", "artery", "Kiddismax", "RCTaround", "AFAaround",
  "Subcutmax", "TP01", "Alb01", "TC01"}, {{107, 5, 0, 16},
  {{95.53571428571429`, 100.`}, {100.`, 76.19047619047619`}, 0.9609375`}}},
{{"Dage", "artery", "Kiddismax", "RCTaround", "AFAaround",
  "Subcutmax", "TP01", "TG01", "TC01"}, {{107, 5, 0, 16},
  {{95.53571428571429`, 100.`}, {100.`, 76.19047619047619`}, 0.9609375`}}},
{{"Dage", "artery", "Kiddismax", "RCTaround", "AFAaround",
  "Subcutmax", "Alb01", "TG01", "TC01"}, {{107, 5, 0, 16},
  {{95.53571428571429`, 100.`}, {100.`, 76.19047619047619`}, 0.9609375`}}},
{{"Dage", "artery", "Kiddismax", "RCTaround", "AFAaround",
  "TP01", "Alb01", "TG01", "TC01"}, {{107, 5, 0, 16},
  {{95.53571428571429`, 100.`}, {100.`, 76.19047619047619`}, 0.9609375`}}},
{{"Dage", "artery", "Kiddismax", "AFAaround", "Subcutmax",
  "TP01", "Alb01", "TG01", "TC01"}, {{107, 5, 0, 16},
  {{95.53571428571429`, 100.`}, {100.`, 76.19047619047619`}, 0.9609375`}}},
{{"Dage", "Kiddismax", "Kiddis内", "Kiddis外", "RCTaround",
  "AFAaround", "Subcutmax", "TP01", "TC01"}, {{107, 5, 0, 16},
  {{95.53571428571429`, 100.`}, {100.`, 76.19047619047619`}, 0.9609375`}}},
{{"Dage", "Kiddismax", "Kiddis内", "Kiddis外", "RCTaround",
  "AFAaround", "TP01", "Alb01", "TC01"}, {{107, 5, 0, 16},
  {{95.53571428571429`, 100.`}, {100.`, 76.19047619047619`}, 0.9609375`}}},
{{"Dage", "Kiddismax", "Kiddis内", "RCTaround", "AFAaround",
  "Subcutmax", "TP01", "Alb01", "TC01"}, {{107, 5, 0, 16},
  {{95.53571428571429`, 100.`}, {100.`, 76.19047619047619`}, 0.9609375`}}},
{{"artery", "Kiddismax", "Kiddis内", "Kiddis外", "RFAaround",
  "RCTaround", "AFAaround", "TP01", "TC01"}, {{107, 5, 0, 16},
  {{95.53571428571429`, 100.`}, {100.`, 76.19047619047619`}, 0.9609375`}}},
{{"artery", "Kiddismax", "Kiddis内", "Kiddis外", "RCTaround",
  "AFAaround", "Subcutmax", "TP01", "TC01"}, {{107, 5, 0, 16},
  {{95.53571428571429`, 100.`}, {100.`, 76.19047619047619`}, 0.9609375`}}},
{{"artery", "Kiddismax", "Kiddis内", "Kiddis外", "RCTaround",
  "AFAaround", "Subcutmax", "TG01", "TC01"}, {{107, 5, 0, 16},
  {{95.53571428571429`, 100.`}, {100.`, 76.19047619047619`}, 0.9609375`}}},
{{"artery", "Kiddismax", "Kiddis内", "Kiddis外", "RCTaround",
  "AFAaround", "TP01", "TG01", "TC01"}, {{107, 5, 0, 16},
  {{95.53571428571429`, 100.`}, {100.`, 76.19047619047619`}, 0.9609375`}}},
{{"artery", "Kiddismax", "Kiddis内", "RFAaround", "RCTaround",
  "AFAaround", "Subcutmax", "TP01", "TC01"}, {{107, 5, 0, 16},
  {{95.53571428571429`, 100.`}, {100.`, 76.19047619047619`}, 0.9609375`}}},
{{"artery", "Kiddismax", "Kiddis内", "RFAaround", "RCTaround",
  "AFAaround", "TP01", "Alb01", "TC01"}, {{107, 5, 0, 16},
  {{95.53571428571429`, 100.`}, {100.`, 76.19047619047619`}, 0.9609375`}}},
{{"artery", "Kiddismax", "Kiddis内", "RFAaround", "RCTaround",
  "AFAaround", "TP01", "TG01", "TC01"}, {{107, 5, 0, 16},
  {{95.53571428571429`, 100.`}, {100.`, 76.19047619047619`}, 0.9609375`}}},
{{"artery", "Kiddismax", "Kiddis外", "RCTaround", "AFAaround",
```

```
    "Subcutmax", "TP01", "Alb01", "TC01"}, {{107, 5, 0, 16},
    {{95.53571428571429`, 100.`}, {100.`, 76.19047619047619`}, 0.9609375`}}},
  {{"artery", "Kiddismax", "Kiddis外", "RCTaround", "AFAaround",
    "Subcutmax", "TP01", "TG01", "TC01"}, {{107, 5, 0, 16},
    {{95.53571428571429`, 100.`}, {100.`, 76.19047619047619`}, 0.9609375`}}},
  {{"artery", "Kiddismax", "Kiddis外", "RCTaround", "AFAaround",
    "TP01", "Alb01", "TG01", "TC01"}, {{107, 5, 0, 16},
    {{95.53571428571429`, 100.`}, {100.`, 76.19047619047619`}, 0.9609375`}}},
  {{"Kiddismax", "Kiddis内", "Kiddis外", "RCTaround", "AFAaround",
    "Subcutmax", "TP01", "Alb01", "TC01"}, {{107, 5, 0, 16},
    {{95.53571428571429`, 100.`}, {100.`, 76.19047619047619`}, 0.9609375`}}},
  {{"Dht", "Dbw", "Dage", "artery", "Kiddismax", "Kiddis内",
    "AFAaround", "TP01", "TG01", "TC01"}, {{107, 5, 0, 16},
    {{95.53571428571429`, 100.`}, {100.`, 76.19047619047619`}, 0.9609375`}}},
  {{"Dht", "Dbw", "Dage", "artery", "Kiddismax", "Kiddis外",
    "RCTaround", "AFAaround", "TP01", "TC01"}, {{107, 5, 0, 16},
    {{95.53571428571429`, 100.`}, {100.`, 76.19047619047619`}, 0.9609375`}}},
  {{"Dht", "Dbw", "Dage", "artery", "Kiddismax", "Kiddis外",
    "AFAaround", "TP01", "TG01", "TC01"}, {{107, 5, 0, 16},
    {{95.53571428571429`, 100.`}, {100.`, 76.19047619047619`}, 0.9609375`}}},
  {{"Dht", "Dbw", "Dage", "artery", "Kiddismax", "RCTaround",
    "AFAaround", "TP01", "Alb01", "TC01"}, {{107, 5, 0, 16},
    {{95.53571428571429`, 100.`}, {100.`, 76.19047619047619`}, 0.9609375`}}},
  {{"Dht", "Dbw", "Dage", "artery", "Kiddismax", "RCTaround",
    "AFAaround", "TP01", "TG01", "TC01"}, {{107, 5, 0, 16},
    {{95.53571428571429`, 100.`}, {100.`, 76.19047619047619`}, 0.9609375`}}},
  {{"Dht", "Dbw", "Dage", "artery", "Kiddismax", "RCTaround",
    "AFAaround", "Alb01", "TG01", "TC01"}, {{107, 5, 0, 16},
    {{95.53571428571429`, 100.`}, {100.`, 76.19047619047619`}, 0.9609375`}}},
  {{"Dht", "Dbw", "Dage", "Kiddismax", "Kiddis内", "Kiddis外",
    "AFAaround", "Subcutmax", "TP01", "TC01"}, {{107, 5, 0, 16},
    {{95.53571428571429`, 100.`}, {100.`, 76.19047619047619`}, 0.9609375`}}},
  {{"Dht", "Dbw", "Dage", "Kiddismax", "Kiddis内", "AFAaround",
    "Subcutmax", "TP01", "Alb01", "TC01"}, {{107, 5, 0, 16},
    {{95.53571428571429`, 100.`}, {100.`, 76.19047619047619`}, 0.9609375`}}},
  {{"Dht", "Dbw", "Dage", "Kiddismax", "Kiddis外", "AFAaround",
    "Subcutmax", "TP01", "Alb01", "TC01"}, {{107, 5, 0, 16},
    {{95.53571428571429`, 100.`}, {100.`, 76.19047619047619`}, 0.9609375`}}},
  {{"Dht", "Dbw", "Dage", "Kiddismax", "AFAaround", "Subcutmax",
    "TP01", "Alb01", "TG01", "TC01"}, {{107, 5, 0, 16},
    {{95.53571428571429`, 100.`}, {100.`, 76.19047619047619`}, 0.9609375`}}},
  {{"Dht", "Dbw", "artery", "Kiddismax", "Kiddis内", "Kiddis外",
    "RCTaround", "AFAaround", "TP01", "TC01"}, {{107, 5, 0, 16},
    {{95.53571428571429`, 100.`}, {100.`, 76.19047619047619`}, 0.9609375`}}},
  {{"Dht", "Dbw", "artery", "Kiddismax", "Kiddis外", "RCTaround",
    "AFAaround", "Subcutmax", "TP01", "TC01"}, {{107, 5, 0, 16},
```

```
  {{95.53571428571429`, 100.`}, {100.`, 76.19047619047619`}, 0.9609375`}}},
{{"Dht", "Dbw", "artery", "Kiddismax", "Kiddis外", "RCTaround",
  "AFAaround", "TP01", "Alb01", "TC01"}, {{107, 5, 0, 16},
  {{95.53571428571429`, 100.`}, {100.`, 76.19047619047619`}, 0.9609375`}}},
{{"Dht", "Dbw", "artery", "Kiddismax", "Kiddis外", "RCTaround",
  "AFAaround", "TP01", "TG01", "TC01"}, {{107, 5, 0, 16},
  {{95.53571428571429`, 100.`}, {100.`, 76.19047619047619`}, 0.9609375`}}},
{{"Dht", "Dbw", "Kiddismax", "Kiddis内", "Kiddis外", "RCTaround",
  "AFAaround", "Subcutmax", "TP01", "TC01"}, {{107, 5, 0, 16},
  {{95.53571428571429`, 100.`}, {100.`, 76.19047619047619`}, 0.9609375`}}},
{{"Dht", "Dsex", "Dage", "artery", "Kiddismax", "RCTaround",
  "AFAaround", "TP01", "Alb01", "TC01"}, {{107, 5, 0, 16},
  {{95.53571428571429`, 100.`}, {100.`, 76.19047619047619`}, 0.9609375`}}},
{{"Dht", "Dage", "artery", "Kiddismax", "Kiddis内", "RCTaround",
  "AFAaround", "Subcutmax", "TG01", "TC01"}, {{107, 5, 0, 16},
  {{95.53571428571429`, 100.`}, {100.`, 76.19047619047619`}, 0.9609375`}}},
{{"Dht", "Dage", "artery", "Kiddismax", "Kiddis内", "RCTaround",
  "AFAaround", "TP01", "TG01", "TC01"}, {{107, 5, 0, 16},
  {{95.53571428571429`, 100.`}, {100.`, 76.19047619047619`}, 0.9609375`}}},
{{"Dht", "Dage", "artery", "Kiddismax", "Kiddis外", "RCTaround",
  "AFAaround", "Subcutmax", "TP01", "TC01"}, {{107, 5, 0, 16},
  {{95.53571428571429`, 100.`}, {100.`, 76.19047619047619`}, 0.9609375`}}},
{{"Dht", "Dage", "artery", "Kiddismax", "Kiddis外", "RCTaround",
  "AFAaround", "Subcutmax", "Alb01", "TC01"}, {{107, 5, 0, 16},
  {{95.53571428571429`, 100.`}, {100.`, 76.19047619047619`}, 0.9609375`}}},
{{"Dht", "Dage", "artery", "Kiddismax", "Kiddis外", "RCTaround",
  "AFAaround", "TP01", "Alb01", "TC01"}, {{107, 5, 0, 16},
  {{95.53571428571429`, 100.`}, {100.`, 76.19047619047619`}, 0.9609375`}}},
{{"Dht", "Dage", "artery", "Kiddismax", "Kiddis外", "RCTaround",
  "AFAaround", "TP01", "TG01", "TC01"}, {{107, 5, 0, 16},
  {{95.53571428571429`, 100.`}, {100.`, 76.19047619047619`}, 0.9609375`}}},
{{"Dht", "Dage", "artery", "Kiddismax", "RCTaround", "AFAaround",
  "Subcutmax", "TP01", "Alb01", "TC01"}, {{107, 5, 0, 16},
  {{95.53571428571429`, 100.`}, {100.`, 76.19047619047619`}, 0.9609375`}}},
{{"Dht", "Dage", "artery", "Kiddismax", "RCTaround", "AFAaround",
  "Subcutmax", "TP01", "TG01", "TC01"}, {{107, 5, 0, 16},
  {{95.53571428571429`, 100.`}, {100.`, 76.19047619047619`}, 0.9609375`}}},
{{"Dht", "Dage", "artery", "Kiddismax", "RCTaround", "AFAaround",
  "Subcutmax", "Alb01", "TG01", "TC01"}, {{107, 5, 0, 16},
  {{95.53571428571429`, 100.`}, {100.`, 76.19047619047619`}, 0.9609375`}}},
{{"Dht", "Dage", "artery", "Kiddismax", "RCTaround", "AFAaround",
  "TP01", "Alb01", "TG01", "TC01"}, {{107, 5, 0, 16},
  {{95.53571428571429`, 100.`}, {100.`, 76.19047619047619`}, 0.9609375`}}},
{{"Dht", "Dage", "Kiddismax", "Kiddis内", "Kiddis外", "RCTaround",
  "AFAaround", "Subcutmax", "TP01", "TC01"}, {{107, 5, 0, 16},
  {{95.53571428571429`, 100.`}, {100.`, 76.19047619047619`}, 0.9609375`}}},
```

```
{{"Dht", "Dage", "Kiddismax", "Kiddis内", "Kiddis外", "RCTaround",
  "AFAaround", "TP01", "Alb01", "TC01"}, {{107, 5, 0, 16},
  {{95.53571428571429`, 100.`}, {100.`, 76.19047619047619`}, 0.9609375`}}},
{{"Dht", "Dage", "Kiddismax", "Kiddis内", "RCTaround", "AFAaround",
  "Subcutmax", "TP01", "Alb01", "TC01"}, {{107, 5, 0, 16},
  {{95.53571428571429`, 100.`}, {100.`, 76.19047619047619`}, 0.9609375`}}},
{{"Dht", "artery", "Kiddismax", "Kiddis内", "Kiddis外", "RCTaround",
  "AFAaround", "Subcutmax", "TP01", "TC01"}, {{107, 5, 0, 16},
  {{95.53571428571429`, 100.`}, {100.`, 76.19047619047619`}, 0.9609375`}}},
{{"Dht", "artery", "Kiddismax", "Kiddis内", "Kiddis外", "RCTaround",
  "AFAaround", "Subcutmax", "TG01", "TC01"}, {{107, 5, 0, 16},
  {{95.53571428571429`, 100.`}, {100.`, 76.19047619047619`}, 0.9609375`}}},
{{"Dht", "artery", "Kiddismax", "Kiddis内", "Kiddis外", "RCTaround",
  "AFAaround", "TP01", "TG01", "TC01"}, {{107, 5, 0, 16},
  {{95.53571428571429`, 100.`}, {100.`, 76.19047619047619`}, 0.9609375`}}},
{{"Dht", "artery", "Kiddismax", "Kiddis外", "RCTaround", "AFAaround",
  "Subcutmax", "TP01", "Alb01", "TC01"}, {{107, 5, 0, 16},
  {{95.53571428571429`, 100.`}, {100.`, 76.19047619047619`}, 0.9609375`}}},
{{"Dht", "artery", "Kiddismax", "Kiddis外", "RCTaround", "AFAaround",
  "Subcutmax", "TP01", "TG01", "TC01"}, {{107, 5, 0, 16},
  {{95.53571428571429`, 100.`}, {100.`, 76.19047619047619`}, 0.9609375`}}},
{{"Dht", "artery", "Kiddismax", "Kiddis外", "RCTaround", "AFAaround",
  "TP01", "Alb01", "TG01", "TC01"}, {{107, 5, 0, 16},
  {{95.53571428571429`, 100.`}, {100.`, 76.19047619047619`}, 0.9609375`}}},
{{"Dht", "Kiddismax", "Kiddis内", "Kiddis外", "RCTaround", "AFAaround",
  "Subcutmax", "TP01", "Alb01", "TC01"}, {{107, 5, 0, 16},
  {{95.53571428571429`, 100.`}, {100.`, 76.19047619047619`}, 0.9609375`}}},
{{"Dht", "Kiddismax", "Kiddis内", "RCTaround", "AFAaround",
  "Subcutmax", "TP01", "Alb01", "TG01", "TC01"}, {{107, 5, 0, 16},
  {{95.53571428571429`, 100.`}, {100.`, 76.19047619047619`}, 0.9609375`}}},
{{"Dbw", "Dsex", "Dage", "artery", "Kiddismax", "Kiddis外",
  "RCTaround", "AFAaround", "TP01", "TC01"}, {{107, 5, 0, 16},
  {{95.53571428571429`, 100.`}, {100.`, 76.19047619047619`}, 0.9609375`}}},
{{"Dbw", "Dsex", "Dage", "artery", "Kiddismax", "RCTaround",
  "AFAaround", "TP01", "Alb01", "TC01"}, {{107, 5, 0, 16},
  {{95.53571428571429`, 100.`}, {100.`, 76.19047619047619`}, 0.9609375`}}},
{{"Dbw", "Dsex", "Dage", "artery", "Kiddismax", "RCTaround",
  "AFAaround", "Alb01", "TG01", "TC01"}, {{107, 5, 0, 16},
  {{95.53571428571429`, 100.`}, {100.`, 76.19047619047619`}, 0.9609375`}}},
{{"Dbw", "Dage", "artery", "Kiddismax", "Kiddis外", "RCTaround",
  "AFAaround", "Subcutmax", "TP01", "TC01"}, {{107, 5, 0, 16},
  {{95.53571428571429`, 100.`}, {100.`, 76.19047619047619`}, 0.9609375`}}},
{{"Dbw", "Dage", "artery", "Kiddismax", "Kiddis外", "RCTaround",
  "AFAaround", "TP01", "Alb01", "TC01"}, {{107, 5, 0, 16},
  {{95.53571428571429`, 100.`}, {100.`, 76.19047619047619`}, 0.9609375`}}},
```

```
{{"Dbw", "Dage", "artery", "Kiddismax", "Kiddis外", "RCTaround",
  "AFAaround", "TP01", "TG01", "TC01"}, {{107, 5, 0, 16},
  {{95.53571428571429`, 100.`}, {100.`, 76.19047619047619`}, 0.9609375`}}},
{{"Dbw", "Dage", "artery", "Kiddismax", "Kiddis外", "RCTaround",
  "AFAaround", "Alb01", "TG01", "TC01"}, {{107, 5, 0, 16},
  {{95.53571428571429`, 100.`}, {100.`, 76.19047619047619`}, 0.9609375`}}},
{{"Dbw", "Dage", "artery", "Kiddismax", "RCTaround", "AFAaround",
  "Subcutmax", "TP01", "TG01", "TC01"}, {{107, 5, 0, 16},
  {{95.53571428571429`, 100.`}, {100.`, 76.19047619047619`}, 0.9609375`}}},
{{"Dbw", "Dage", "artery", "Kiddismax", "RCTaround", "AFAaround",
  "Subcutmax", "Alb01", "TG01", "TC01"}, {{107, 5, 0, 16},
  {{95.53571428571429`, 100.`}, {100.`, 76.19047619047619`}, 0.9609375`}}},
{{"Dbw", "Dage", "artery", "Kiddismax", "RCTaround", "AFAaround",
  "TP01", "Alb01", "TG01", "TC01"}, {{107, 5, 0, 16},
  {{95.53571428571429`, 100.`}, {100.`, 76.19047619047619`}, 0.9609375`}}},
{{"Dbw", "Dage", "Kiddismax", "Kiddis内", "Kiddis外", "RCTaround",
  "AFAaround", "Subcutmax", "TP01", "TC01"}, {{107, 5, 0, 16},
  {{95.53571428571429`, 100.`}, {100.`, 76.19047619047619`}, 0.9609375`}}},
{{"Dbw", "Dage", "Kiddismax", "Kiddis内", "Kiddis外", "RCTaround",
  "AFAaround", "Subcutmax", "Alb01", "TC01"}, {{107, 5, 0, 16},
  {{95.53571428571429`, 100.`}, {100.`, 76.19047619047619`}, 0.9609375`}}},
{{"Dbw", "Dage", "Kiddismax", "Kiddis内", "Kiddis外", "RCTaround",
  "AFAaround", "TP01", "Alb01", "TC01"}, {{107, 5, 0, 16},
  {{95.53571428571429`, 100.`}, {100.`, 76.19047619047619`}, 0.9609375`}}},
{{"Dbw", "Dage", "Kiddismax", "Kiddis内", "Kiddis外", "AFAaround",
  "Subcutmax", "TP01", "Alb01", "TC01"}, {{107, 5, 0, 16},
  {{95.53571428571429`, 100.`}, {100.`, 76.19047619047619`}, 0.9609375`}}},
{{"Dbw", "Dage", "Kiddismax", "Kiddis内", "Kiddis外", "AFAaround",
  "Subcutmax", "TP01", "TG01", "TC01"}, {{107, 5, 0, 16},
  {{95.53571428571429`, 100.`}, {100.`, 76.19047619047619`}, 0.9609375`}}},
{{"Dbw", "Dage", "Kiddismax", "Kiddis内", "RCTaround", "AFAaround",
  "Subcutmax", "TP01", "Alb01", "TC01"}, {{107, 5, 0, 16},
  {{95.53571428571429`, 100.`}, {100.`, 76.19047619047619`}, 0.9609375`}}},
{{"Dbw", "artery", "Kiddismax", "Kiddis内", "Kiddis外", "RCTaround",
  "AFAaround", "Subcutmax", "TG01", "TC01"}, {{107, 5, 0, 16},
  {{95.53571428571429`, 100.`}, {100.`, 76.19047619047619`}, 0.9609375`}}},
{{"Dbw", "artery", "Kiddismax", "Kiddis内", "Kiddis外", "RCTaround",
  "AFAaround", "TP01", "TG01", "TC01"}, {{107, 5, 0, 16},
  {{95.53571428571429`, 100.`}, {100.`, 76.19047619047619`}, 0.9609375`}}},
{{"Dbw", "artery", "Kiddismax", "Kiddis外", "RCTaround", "AFAaround",
  "Subcutmax", "TP01", "Alb01", "TC01"}, {{107, 5, 0, 16},
  {{95.53571428571429`, 100.`}, {100.`, 76.19047619047619`}, 0.9609375`}}},
{{"Dbw", "artery", "Kiddismax", "Kiddis外", "RCTaround", "AFAaround",
  "Subcutmax", "TP01", "TG01", "TC01"}, {{107, 5, 0, 16},
  {{95.53571428571429`, 100.`}, {100.`, 76.19047619047619`}, 0.9609375`}}},
{{"Dbw", "artery", "Kiddismax", "Kiddis外", "RCTaround", "AFAaround",
```

```
  "TP01", "Alb01", "TG01", "TC01"}, {{107, 5, 0, 16},
  {{95.53571428571429`, 100.`}, {100.`, 76.19047619047619`}, 0.9609375`}}},
{{"Dbw", "Kiddismax", "Kiddis内", "Kiddis外", "RCTaround", "AFAaround",
  "Subcutmax", "TP01", "Alb01", "TC01"}, {{107, 5, 0, 16},
  {{95.53571428571429`, 100.`}, {100.`, 76.19047619047619`}, 0.9609375`}}},
{{"Dsex", "Dage", "artery", "Kiddismax", "Kiddis外", "RCTaround",
  "AFAaround", "Subcutmax", "TP01", "TC01"}, {{107, 5, 0, 16},
  {{95.53571428571429`, 100.`}, {100.`, 76.19047619047619`}, 0.9609375`}}},
{{"Dsex", "Dage", "artery", "Kiddismax", "Kiddis外", "RCTaround",
  "AFAaround", "Subcutmax", "Alb01", "TC01"}, {{107, 5, 0, 16},
  {{95.53571428571429`, 100.`}, {100.`, 76.19047619047619`}, 0.9609375`}}},
{{"Dsex", "Dage", "artery", "Kiddismax", "Kiddis外", "RCTaround",
  "AFAaround", "TP01", "Alb01", "TC01"}, {{107, 5, 0, 16},
  {{95.53571428571429`, 100.`}, {100.`, 76.19047619047619`}, 0.9609375`}}},
{{"Dsex", "Dage", "artery", "Kiddismax", "RCTaround", "AFAaround",
  "TP01", "Alb01", "TG01", "TC01"}, {{107, 5, 0, 16},
  {{95.53571428571429`, 100.`}, {100.`, 76.19047619047619`}, 0.9609375`}}},
{{"Dsex", "Dage", "Kiddismax", "Kiddis内", "Kiddis外", "RCTaround",
  "AFAaround", "TP01", "Alb01", "TC01"}, {{107, 5, 0, 16},
  {{95.53571428571429`, 100.`}, {100.`, 76.19047619047619`}, 0.9609375`}}},
{{"Dsex", "Dage", "Kiddismax", "Kiddis内", "RCTaround", "AFAaround",
  "Subcutmax", "TP01", "Alb01", "TC01"}, {{107, 5, 0, 16},
  {{95.53571428571429`, 100.`}, {100.`, 76.19047619047619`}, 0.9609375`}}},
{{"Dsex", "Kiddismax", "Kiddis内", "Kiddis外", "RCTaround", "AFAaround",
  "Subcutmax", "TP01", "Alb01", "TC01"}, {{107, 5, 0, 16},
  {{95.53571428571429`, 100.`}, {100.`, 76.19047619047619`}, 0.9609375`}}},
{{"Dage", "artery", "Kiddismax", "Kiddis内", "Kiddis外", "RFAaround",
  "RCTaround", "AFAaround", "Subcutmax", "TC01"}, {{107, 5, 0, 16},
  {{95.53571428571429`, 100.`}, {100.`, 76.19047619047619`}, 0.9609375`}}},
{{"Dage", "artery", "Kiddismax", "Kiddis内", "Kiddis外", "RFAaround",
  "RCTaround", "AFAaround", "TP01", "TC01"}, {{107, 5, 0, 16},
  {{95.53571428571429`, 100.`}, {100.`, 76.19047619047619`}, 0.9609375`}}},
{{"Dage", "artery", "Kiddismax", "Kiddis内", "Kiddis外", "RFAaround",
  "RCTaround", "AFAaround", "TG01", "TC01"}, {{107, 5, 0, 16},
  {{95.53571428571429`, 100.`}, {100.`, 76.19047619047619`}, 0.9609375`}}},
{{"Dage", "artery", "Kiddismax", "Kiddis内", "Kiddis外", "RFAaround",
  "RCTaround", "Subcutmax", "TP01", "TC01"}, {{107, 5, 0, 16},
  {{95.53571428571429`, 100.`}, {100.`, 76.19047619047619`}, 0.9609375`}}},
{{"Dage", "artery", "Kiddismax", "Kiddis内", "Kiddis外", "RCTaround",
  "AFAaround", "Subcutmax", "TP01", "TC01"}, {{107, 5, 0, 16},
  {{95.53571428571429`, 100.`}, {100.`, 76.19047619047619`}, 0.9609375`}}},
{{"Dage", "artery", "Kiddismax", "Kiddis内", "Kiddis外", "RCTaround",
  "AFAaround", "Subcutmax", "TG01", "TC01"}, {{107, 5, 0, 16},
  {{95.53571428571429`, 100.`}, {100.`, 76.19047619047619`}, 0.9609375`}}},
{{"Dage", "artery", "Kiddismax", "Kiddis内", "Kiddis外", "RCTaround",
```

```
  "AFAaround", "TP01", "TG01", "TC01"}, {{107, 5, 0, 16},
  {{95.53571428571429`, 100.`}, {100.`, 76.19047619047619`}, 0.9609375`}}},
{{"Dage", "artery", "Kiddismax", "Kiddis内", "RFAaround", "RCTaround",
  "AFAaround", "Subcutmax", "TP01", "TC01"}, {{107, 5, 0, 16},
  {{95.53571428571429`, 100.`}, {100.`, 76.19047619047619`}, 0.9609375`}}},
{{"Dage", "artery", "Kiddismax", "Kiddis内", "RFAaround", "RCTaround",
  "AFAaround", "Subcutmax", "Alb01", "TC01"}, {{107, 5, 0, 16},
  {{95.53571428571429`, 100.`}, {100.`, 76.19047619047619`}, 0.9609375`}}},
{{"Dage", "artery", "Kiddismax", "Kiddis内", "RFAaround", "RCTaround",
  "AFAaround", "Subcutmax", "TG01", "TC01"}, {{107, 5, 0, 16},
  {{95.53571428571429`, 100.`}, {100.`, 76.19047619047619`}, 0.9609375`}}},
{{"Dage", "artery", "Kiddismax", "Kiddis内", "RFAaround", "RCTaround",
  "AFAaround", "TP01", "Alb01", "TC01"}, {{107, 5, 0, 16},
  {{95.53571428571429`, 100.`}, {100.`, 76.19047619047619`}, 0.9609375`}}},
{{"Dage", "artery", "Kiddismax", "Kiddis内", "RFAaround", "RCTaround",
  "AFAaround", "TP01", "TG01", "TC01"}, {{107, 5, 0, 16},
  {{95.53571428571429`, 100.`}, {100.`, 76.19047619047619`}, 0.9609375`}}},
{{"Dage", "artery", "Kiddismax", "Kiddis内", "RFAaround", "RCTaround",
  "AFAaround", "Alb01", "TG01", "TC01"}, {{107, 5, 0, 16},
  {{95.53571428571429`, 100.`}, {100.`, 76.19047619047619`}, 0.9609375`}}},
{{"Dage", "artery", "Kiddismax", "Kiddis外", "RCTaround", "AFAaround",
  "Subcutmax", "TP01", "Alb01", "TC01"}, {{107, 5, 0, 16},
  {{95.53571428571429`, 100.`}, {100.`, 76.19047619047619`}, 0.9609375`}}},
{{"Dage", "artery", "Kiddismax", "Kiddis外", "RCTaround", "AFAaround",
  "Subcutmax", "TP01", "TG01", "TC01"}, {{107, 5, 0, 16},
  {{95.53571428571429`, 100.`}, {100.`, 76.19047619047619`}, 0.9609375`}}},
{{"Dage", "artery", "Kiddismax", "Kiddis外", "RCTaround", "AFAaround",
  "Subcutmax", "Alb01", "TG01", "TC01"}, {{107, 5, 0, 16},
  {{95.53571428571429`, 100.`}, {100.`, 76.19047619047619`}, 0.9609375`}}},
{{"Dage", "artery", "Kiddismax", "Kiddis外", "RCTaround",
  "AFAaround", "TP01", "Alb01", "TG01", "TC01"}, {{107, 5, 0, 16},
  {{95.53571428571429`, 100.`}, {100.`, 76.19047619047619`}, 0.9609375`}}},
{{"Dage", "artery", "Kiddismax", "RCTaround", "AFAaround",
  "Subcutmax", "TP01", "Alb01", "TG01", "TC01"}, {{107, 5, 0, 16},
  {{95.53571428571429`, 100.`}, {100.`, 76.19047619047619`}, 0.9609375`}}},
{{"Dage", "Kiddismax", "Kiddis内", "Kiddis外", "RCTaround", "AFAaround",
  "Subcutmax", "TP01", "Alb01", "TC01"}, {{107, 5, 0, 16},
  {{95.53571428571429`, 100.`}, {100.`, 76.19047619047619`}, 0.9609375`}}},
{{"Dage", "Kiddismax", "Kiddis内", "Kiddis外", "RCTaround",
  "AFAaround", "Subcutmax", "TP01", "TG01", "TC01"}, {{107, 5, 0, 16},
  {{95.53571428571429`, 100.`}, {100.`, 76.19047619047619`}, 0.9609375`}}},
{{"Dage", "Kiddismax", "Kiddis内", "Kiddis外", "RCTaround",
  "AFAaround", "TP01", "Alb01", "TG01", "TC01"}, {{107, 5, 0, 16},
  {{95.53571428571429`, 100.`}, {100.`, 76.19047619047619`}, 0.9609375`}}},
{{"Dage", "Kiddismax", "Kiddis内", "RCTaround", "AFAaround",
```

```
"Subcutmax", "TP01", "Alb01", "TG01", "TC01"}, {{107, 5, 0, 16},
  {{95.53571428571429`, 100.`}, {100.`, 76.19047619047619`}, 0.9609375`}}},
{{"artery", "Kiddismax", "Kiddis内", "Kiddis外", "RFAaround",
  "RCTaround", "AFAaround", "TP01", "Alb01", "TC01"}, {{107, 5, 0, 16},
  {{95.53571428571429`, 100.`}, {100.`, 76.19047619047619`}, 0.9609375`}}},
{{"artery", "Kiddismax", "Kiddis内", "Kiddis外", "RFAaround",
  "RCTaround", "AFAaround", "TP01", "TG01", "TC01"}, {{107, 5, 0, 16},
  {{95.53571428571429`, 100.`}, {100.`, 76.19047619047619`}, 0.9609375`}}},
{{"artery", "Kiddismax", "Kiddis内", "Kiddis外", "RCTaround",
  "AFAaround", "Subcutmax", "TP01", "Alb01", "TC01"}, {{107, 5, 0, 16},
  {{95.53571428571429`, 100.`}, {100.`, 76.19047619047619`}, 0.9609375`}}},
{{"artery", "Kiddismax", "Kiddis内", "Kiddis外", "RCTaround",
  "AFAaround", "Subcutmax", "TP01", "TG01", "TC01"}, {{107, 5, 0, 16},
  {{95.53571428571429`, 100.`}, {100.`, 76.19047619047619`}, 0.9609375`}}},
{{"artery", "Kiddismax", "Kiddis内", "Kiddis外", "RCTaround",
  "AFAaround", "Subcutmax", "Alb01", "TG01", "TC01"}, {{107, 5, 0, 16},
  {{95.53571428571429`, 100.`}, {100.`, 76.19047619047619`}, 0.9609375`}}},
{{"artery", "Kiddismax", "Kiddis内", "Kiddis外", "RCTaround",
  "AFAaround", "TP01", "Alb01", "TG01", "TC01"}, {{107, 5, 0, 16},
  {{95.53571428571429`, 100.`}, {100.`, 76.19047619047619`}, 0.9609375`}}},
{{"artery", "Kiddismax", "Kiddis内", "RFAaround", "RCTaround",
  "AFAaround", "Subcutmax", "TP01", "Alb01", "TC01"}, {{107, 5, 0, 16},
  {{95.53571428571429`, 100.`}, {100.`, 76.19047619047619`}, 0.9609375`}}},
{{"artery", "Kiddismax", "Kiddis内", "RFAaround", "RCTaround",
  "AFAaround", "Subcutmax", "TP01", "TG01", "TC01"}, {{107, 5, 0, 16},
  {{95.53571428571429`, 100.`}, {100.`, 76.19047619047619`}, 0.9609375`}}},
{{"artery", "Kiddismax", "Kiddis内", "RFAaround", "RCTaround",
  "AFAaround", "TP01", "Alb01", "TG01", "TC01"}, {{107, 5, 0, 16},
  {{95.53571428571429`, 100.`}, {100.`, 76.19047619047619`}, 0.9609375`}}},
{{"artery", "Kiddismax", "Kiddis外", "RCTaround", "AFAaround",
  "Subcutmax", "TP01", "Alb01", "TG01", "TC01"}, {{107, 5, 0, 16},
  {{95.53571428571429`, 100.`}, {100.`, 76.19047619047619`}, 0.9609375`}}},
{{"Kiddismax", "Kiddis内", "Kiddis外", "RCTaround", "AFAaround",
  "Subcutmax", "TP01", "Alb01", "TG01", "TC01"}, {{107, 5, 0, 16},
  {{95.53571428571429`, 100.`}, {100.`, 76.19047619047619`}, 0.9609375`}}},
{{"Dht", "Dbw", "Dsex", "Dage", "artery", "Kiddismax", "Kiddis外",
  "RCTaround", "AFAaround", "TP01", "TC01"}, {{107, 5, 0, 16},
  {{95.53571428571429`, 100.`}, {100.`, 76.19047619047619`}, 0.9609375`}}},
{{"Dht", "Dbw", "Dsex", "Dage", "artery", "Kiddismax", "RCTaround",
  "AFAaround", "TP01", "Alb01", "TC01"}, {{107, 5, 0, 16},
  {{95.53571428571429`, 100.`}, {100.`, 76.19047619047619`}, 0.9609375`}}},
{{"Dht", "Dbw", "Dsex", "Dage", "artery", "Kiddismax", "RCTaround",
  "AFAaround", "Alb01", "TG01", "TC01"}, {{107, 5, 0, 16},
  {{95.53571428571429`, 100.`}, {100.`, 76.19047619047619`}, 0.9609375`}}},
{{"Dht", "Dbw", "Dsex", "Dage", "artery", "Kiddismax", "AFAaround",
  "TP01", "Alb01", "TG01", "TC01"}, {{107, 5, 0, 16},
```

```
  {{95.53571428571429`, 100.`}, {100.`, 76.19047619047619`}, 0.9609375`}}},
{{"Dht", "Dbw", "Dage", "artery", "Kiddismax", "Kiddis内", "RCTaround",
  "AFAaround", "TP01", "TG01", "TC01"}, {{107, 5, 0, 16},
  {{95.53571428571429`, 100.`}, {100.`, 76.19047619047619`}, 0.9609375`}}},
{{"Dht", "Dbw", "Dage", "artery", "Kiddismax", "Kiddis外", "RCTaround",
  "AFAaround", "Subcutmax", "TP01", "TC01"}, {{107, 5, 0, 16},
  {{95.53571428571429`, 100.`}, {100.`, 76.19047619047619`}, 0.9609375`}}},
{{"Dht", "Dbw", "Dage", "artery", "Kiddismax", "Kiddis外", "RCTaround",
  "AFAaround", "TP01", "Alb01", "TC01"}, {{107, 5, 0, 16},
  {{95.53571428571429`, 100.`}, {100.`, 76.19047619047619`}, 0.9609375`}}},
{{"Dht", "Dbw", "Dage", "artery", "Kiddismax", "Kiddis外", "RCTaround",
  "AFAaround", "TP01", "TG01", "TC01"}, {{107, 5, 0, 16},
  {{95.53571428571429`, 100.`}, {100.`, 76.19047619047619`}, 0.9609375`}}},
{{"Dht", "Dbw", "Dage", "artery", "Kiddismax", "Kiddis外", "RCTaround",
  "AFAaround", "Alb01", "TG01", "TC01"}, {{107, 5, 0, 16},
  {{95.53571428571429`, 100.`}, {100.`, 76.19047619047619`}, 0.9609375`}}},
{{"Dht", "Dbw", "Dage", "artery", "Kiddismax", "RCTaround",
  "AFAaround", "Subcutmax", "TP01", "TG01", "TC01"}, {{107, 5, 0, 16},
  {{95.53571428571429`, 100.`}, {100.`, 76.19047619047619`}, 0.9609375`}}},
{{"Dht", "Dbw", "Dage", "artery", "Kiddismax", "RCTaround",
  "AFAaround", "Subcutmax", "Alb01", "TG01", "TC01"}, {{107, 5, 0, 16},
  {{95.53571428571429`, 100.`}, {100.`, 76.19047619047619`}, 0.9609375`}}},
{{"Dht", "Dbw", "Dage", "artery", "Kiddismax", "RCTaround",
  "AFAaround", "TP01", "Alb01", "TG01", "TC01"}, {{107, 5, 0, 16},
  {{95.53571428571429`, 100.`}, {100.`, 76.19047619047619`}, 0.9609375`}}},
{{"Dht", "Dbw", "Dage", "Kiddismax", "Kiddis内", "Kiddis外", "RCTaround",
  "AFAaround", "Subcutmax", "TP01", "TC01"}, {{107, 5, 0, 16},
  {{95.53571428571429`, 100.`}, {100.`, 76.19047619047619`}, 0.9609375`}}},
{{"Dht", "Dbw", "Dage", "Kiddismax", "Kiddis内", "Kiddis外", "RCTaround",
  "AFAaround", "Subcutmax", "Alb01", "TC01"}, {{107, 5, 0, 16},
  {{95.53571428571429`, 100.`}, {100.`, 76.19047619047619`}, 0.9609375`}}},
{{"Dht", "Dbw", "Dage", "Kiddismax", "Kiddis内", "Kiddis外",
  "RCTaround", "AFAaround", "TP01", "Alb01", "TC01"}, {{107, 5, 0, 16},
  {{95.53571428571429`, 100.`}, {100.`, 76.19047619047619`}, 0.9609375`}}},
{{"Dht", "Dbw", "Dage", "Kiddismax", "Kiddis内", "Kiddis外",
  "AFAaround", "Subcutmax", "TP01", "Alb01", "TC01"}, {{107, 5, 0, 16},
  {{95.53571428571429`, 100.`}, {100.`, 76.19047619047619`}, 0.9609375`}}},
{{"Dht", "Dbw", "Dage", "Kiddismax", "Kiddis内", "Kiddis外",
  "AFAaround", "Subcutmax", "TP01", "TG01", "TC01"}, {{107, 5, 0, 16},
  {{95.53571428571429`, 100.`}, {100.`, 76.19047619047619`}, 0.9609375`}}},
{{"Dht", "Dbw", "Dage", "Kiddismax", "Kiddis内", "RCTaround",
  "AFAaround", "Subcutmax", "TP01", "Alb01", "TC01"}, {{107, 5, 0, 16},
  {{95.53571428571429`, 100.`}, {100.`, 76.19047619047619`}, 0.9609375`}}},
{{"Dht", "Dbw", "Dage", "Kiddismax", "Kiddis内", "AFAaround",
  "Subcutmax", "TP01", "Alb01", "TG01", "TC01"}, {{107, 5, 0, 16},
  {{95.53571428571429`, 100.`}, {100.`, 76.19047619047619`}, 0.9609375`}}},
```

```
{{"Dht", "Dbw", "Dage", "Kiddismax", "Kiddis外", "AFAaround",
  "Subcutmax", "TP01", "Alb01", "TG01", "TC01"}, {{107, 5, 0, 16},
  {{95.53571428571429`, 100.`}, {100.`, 76.19047619047619`}, 0.9609375`}}},
{{"Dht", "Dbw", "artery", "Kiddismax", "Kiddis内", "Kiddis外",
  "RCTaround", "AFAaround", "Subcutmax", "TG01", "TC01"}, {{107, 5, 0, 16},
  {{95.53571428571429`, 100.`}, {100.`, 76.19047619047619`}, 0.9609375`}}},
{{"Dht", "Dbw", "artery", "Kiddismax", "Kiddis内", "Kiddis外",
  "RCTaround", "AFAaround", "TP01", "TG01", "TC01"}, {{107, 5, 0, 16},
  {{95.53571428571429`, 100.`}, {100.`, 76.19047619047619`}, 0.9609375`}}},
{{"Dht", "Dbw", "artery", "Kiddismax", "Kiddis外", "RCTaround",
  "AFAaround", "Subcutmax", "TP01", "TG01", "TC01"}, {{107, 5, 0, 16},
  {{95.53571428571429`, 100.`}, {100.`, 76.19047619047619`}, 0.9609375`}}},
{{"Dht", "Dbw", "artery", "Kiddismax", "Kiddis外", "RCTaround",
  "AFAaround", "TP01", "Alb01", "TG01", "TC01"}, {{107, 5, 0, 16},
  {{95.53571428571429`, 100.`}, {100.`, 76.19047619047619`}, 0.9609375`}}},
{{"Dht", "Dbw", "Kiddismax", "Kiddis内", "Kiddis外", "RCTaround",
  "AFAaround", "Subcutmax", "TP01", "Alb01", "TC01"}, {{107, 5, 0, 16},
  {{95.53571428571429`, 100.`}, {100.`, 76.19047619047619`}, 0.9609375`}}},
{{"Dht", "Dbw", "Kiddismax", "Kiddis内", "Kiddis外", "RCTaround",
  "AFAaround", "Subcutmax", "TP01", "TG01", "TC01"}, {{107, 5, 0, 16},
  {{95.53571428571429`, 100.`}, {100.`, 76.19047619047619`}, 0.9609375`}}},
{{"Dht", "Dsex", "Dage", "artery", "Kiddismax", "Kiddis外", "RCTaround",
  "AFAaround", "Subcutmax", "TP01", "TC01"}, {{107, 5, 0, 16},
  {{95.53571428571429`, 100.`}, {100.`, 76.19047619047619`}, 0.9609375`}}},
{{"Dht", "Dsex", "Dage", "artery", "Kiddismax", "Kiddis外", "RCTaround",
  "AFAaround", "Subcutmax", "Alb01", "TC01"}, {{107, 5, 0, 16},
  {{95.53571428571429`, 100.`}, {100.`, 76.19047619047619`}, 0.9609375`}}},
{{"Dht", "Dsex", "Dage", "artery", "Kiddismax", "Kiddis外", "RCTaround",
  "AFAaround", "TP01", "Alb01", "TC01"}, {{107, 5, 0, 16},
  {{95.53571428571429`, 100.`}, {100.`, 76.19047619047619`}, 0.9609375`}}},
{{"Dht", "Dsex", "Dage", "artery", "Kiddismax", "Kiddis外", "AFAaround",
  "Subcutmax", "TP01", "Alb01", "TC01"}, {{107, 5, 0, 16},
  {{95.53571428571429`, 100.`}, {100.`, 76.19047619047619`}, 0.9609375`}}},
{{"Dht", "Dsex", "Dage", "artery", "Kiddismax", "RCTaround",
  "AFAaround", "Subcutmax", "TP01", "Alb01", "TC01"}, {{107, 5, 0, 16},
  {{95.53571428571429`, 100.`}, {100.`, 76.19047619047619`}, 0.9609375`}}},
{{"Dht", "Dsex", "Dage", "artery", "Kiddismax", "RCTaround",
  "AFAaround", "TP01", "Alb01", "TG01", "TC01"}, {{107, 5, 0, 16},
  {{95.53571428571429`, 100.`}, {100.`, 76.19047619047619`}, 0.9609375`}}},
{{"Dht", "Dsex", "Dage", "Kiddismax", "Kiddis内", "Kiddis外",
  "AFAaround", "Subcutmax", "TP01", "Alb01", "TC01"}, {{107, 5, 0, 16},
  {{95.53571428571429`, 100.`}, {100.`, 76.19047619047619`}, 0.9609375`}}},
{{"Dht", "Dsex", "Dage", "Kiddismax", "Kiddis内", "RCTaround",
  "AFAaround", "Subcutmax", "TP01", "Alb01", "TC01"}, {{107, 5, 0, 16},
  {{95.53571428571429`, 100.`}, {100.`, 76.19047619047619`}, 0.9609375`}}},
```

```
{{"Dht", "Dsex", "artery", "Kiddismax", "Kiddis内", "Kiddis外",
  "RCTaround", "AFAaround", "TP01", "TG01", "TC01"}, {{107, 5, 0, 16},
  {{95.53571428571429`, 100.`}, {100.`, 76.19047619047619`}, 0.9609375`}}},
{{"Dht", "Dage", "artery", "Kiddismax", "Kiddis内", "Kiddis外",
  "RCTaround", "AFAaround", "Subcutmax", "TG01", "TC01"}, {{107, 5, 0, 16},
  {{95.53571428571429`, 100.`}, {100.`, 76.19047619047619`}, 0.9609375`}}},
{{"Dht", "Dage", "artery", "Kiddismax", "Kiddis内", "Kiddis外",
  "RCTaround", "AFAaround", "TP01", "TG01", "TC01"}, {{107, 5, 0, 16},
  {{95.53571428571429`, 100.`}, {100.`, 76.19047619047619`}, 0.9609375`}}},
{{"Dht", "Dage", "artery", "Kiddismax", "Kiddis内", "RCTaround",
  "AFAaround", "Subcutmax", "TP01", "TG01", "TC01"}, {{107, 5, 0, 16},
  {{95.53571428571429`, 100.`}, {100.`, 76.19047619047619`}, 0.9609375`}}},
{{"Dht", "Dage", "artery", "Kiddismax", "Kiddis内", "RCTaround",
  "AFAaround", "TP01", "Alb01", "TG01", "TC01"}, {{107, 5, 0, 16},
  {{95.53571428571429`, 100.`}, {100.`, 76.19047619047619`}, 0.9609375`}}},
{{"Dht", "Dage", "artery", "Kiddismax", "Kiddis外", "RCTaround",
  "AFAaround", "Subcutmax", "TP01", "Alb01", "TC01"}, {{107, 5, 0, 16},
  {{95.53571428571429`, 100.`}, {100.`, 76.19047619047619`}, 0.9609375`}}},
{{"Dht", "Dage", "artery", "Kiddismax", "Kiddis外", "RCTaround",
  "AFAaround", "Subcutmax", "TP01", "TG01", "TC01"}, {{107, 5, 0, 16},
  {{95.53571428571429`, 100.`}, {100.`, 76.19047619047619`}, 0.9609375`}}},
{{"Dht", "Dage", "artery", "Kiddismax", "Kiddis外", "RCTaround",
  "AFAaround", "Subcutmax", "Alb01", "TG01", "TC01"}, {{107, 5, 0, 16},
  {{95.53571428571429`, 100.`}, {100.`, 76.19047619047619`}, 0.9609375`}}},
{{"Dht", "Dage", "artery", "Kiddismax", "Kiddis外", "RCTaround",
  "AFAaround", "TP01", "Alb01", "TG01", "TC01"}, {{107, 5, 0, 16},
  {{95.53571428571429`, 100.`}, {100.`, 76.19047619047619`}, 0.9609375`}}},
{{"Dht", "Dage", "artery", "Kiddismax", "RCTaround", "AFAaround",
  "Subcutmax", "TP01", "Alb01", "TG01", "TC01"}, {{107, 5, 0, 16},
  {{95.53571428571429`, 100.`}, {100.`, 76.19047619047619`}, 0.9609375`}}},
{{"Dht", "Dage", "Kiddismax", "Kiddis内", "Kiddis外", "RCTaround",
  "AFAaround", "Subcutmax", "TP01", "Alb01", "TC01"}, {{107, 5, 0, 16},
  {{95.53571428571429`, 100.`}, {100.`, 76.19047619047619`}, 0.9609375`}}},
{{"Dht", "Dage", "Kiddismax", "Kiddis内", "Kiddis外", "RCTaround",
  "AFAaround", "Subcutmax", "TP01", "TG01", "TC01"}, {{107, 5, 0, 16},
  {{95.53571428571429`, 100.`}, {100.`, 76.19047619047619`}, 0.9609375`}}},
{{"Dht", "Dage", "Kiddismax", "Kiddis内", "Kiddis外", "RCTaround",
  "AFAaround", "TP01", "Alb01", "TG01", "TC01"}, {{107, 5, 0, 16},
  {{95.53571428571429`, 100.`}, {100.`, 76.19047619047619`}, 0.9609375`}}},
{{"Dht", "Dage", "Kiddismax", "Kiddis内", "RCTaround", "AFAaround",
  "Subcutmax", "TP01", "Alb01", "TG01", "TC01"}, {{107, 5, 0, 16},
  {{95.53571428571429`, 100.`}, {100.`, 76.19047619047619`}, 0.9609375`}}},
{{"Dht", "artery", "Kiddismax", "Kiddis内", "Kiddis外", "RCTaround",
  "AFAaround", "Subcutmax", "TP01", "Alb01", "TC01"}, {{107, 5, 0, 16},
  {{95.53571428571429`, 100.`}, {100.`, 76.19047619047619`}, 0.9609375`}}},
```

```
{{"Dht", "artery", "Kiddismax", "Kiddis内", "Kiddis外", "RCTaround",
  "AFAaround", "Subcutmax", "TP01", "TG01", "TC01"}, {{107, 5, 0, 16},
  {{95.53571428571429`, 100.`}, {100.`, 76.19047619047619`}, 0.9609375`}}},
{{"Dht", "artery", "Kiddismax", "Kiddis内", "Kiddis外", "RCTaround",
  "AFAaround", "Subcutmax", "Alb01", "TG01", "TC01"}, {{107, 5, 0, 16},
  {{95.53571428571429`, 100.`}, {100.`, 76.19047619047619`}, 0.9609375`}}},
{{"Dht", "artery", "Kiddismax", "Kiddis内", "Kiddis外", "RCTaround",
  "AFAaround", "TP01", "Alb01", "TG01", "TC01"}, {{107, 5, 0, 16},
  {{95.53571428571429`, 100.`}, {100.`, 76.19047619047619`}, 0.9609375`}}},
{{"Dht", "artery", "Kiddismax", "Kiddis外", "RCTaround", "AFAaround",
  "Subcutmax", "TP01", "Alb01", "TG01", "TC01"}, {{107, 5, 0, 16},
  {{95.53571428571429`, 100.`}, {100.`, 76.19047619047619`}, 0.9609375`}}},
{{"Dht", "Kiddismax", "Kiddis内", "Kiddis外", "RCTaround", "AFAaround",
  "Subcutmax", "TP01", "Alb01", "TG01", "TC01"}, {{107, 5, 0, 16},
  {{95.53571428571429`, 100.`}, {100.`, 76.19047619047619`}, 0.9609375`}}},
{{"Dbw", "Dsex", "Dage", "artery", "Kiddismax", "Kiddis外", "RCTaround",
  "AFAaround", "Subcutmax", "TP01", "TC01"}, {{107, 5, 0, 16},
  {{95.53571428571429`, 100.`}, {100.`, 76.19047619047619`}, 0.9609375`}}},
{{"Dbw", "Dsex", "Dage", "artery", "Kiddismax", "Kiddis外", "RCTaround",
  "AFAaround", "TP01", "Alb01", "TC01"}, {{107, 5, 0, 16},
  {{95.53571428571429`, 100.`}, {100.`, 76.19047619047619`}, 0.9609375`}}},
{{"Dbw", "Dsex", "Dage", "artery", "Kiddismax", "Kiddis外",
  "RCTaround", "AFAaround", "TP01", "TG01", "TC01"}, {{107, 5, 0, 16},
  {{95.53571428571429`, 100.`}, {100.`, 76.19047619047619`}, 0.9609375`}}},
{{"Dbw", "Dsex", "Dage", "artery", "Kiddismax", "Kiddis外", "RCTaround",
  "AFAaround", "Alb01", "TG01", "TC01"}, {{107, 5, 0, 16},
  {{95.53571428571429`, 100.`}, {100.`, 76.19047619047619`}, 0.9609375`}}},
{{"Dbw", "Dsex", "Dage", "artery", "Kiddismax", "RCTaround",
  "AFAaround", "TP01", "Alb01", "TG01", "TC01"}, {{107, 5, 0, 16},
  {{95.53571428571429`, 100.`}, {100.`, 76.19047619047619`}, 0.9609375`}}},
{{"Dbw", "Dsex", "Dage", "Kiddismax", "Kiddis内", "Kiddis外", "RCTaround",
  "AFAaround", "Subcutmax", "TP01", "TC01"}, {{107, 5, 0, 16},
  {{95.53571428571429`, 100.`}, {100.`, 76.19047619047619`}, 0.9609375`}}},
{{"Dbw", "Dsex", "Dage", "Kiddismax", "Kiddis内", "Kiddis外",
  "RCTaround", "AFAaround", "TP01", "Alb01", "TC01"}, {{107, 5, 0, 16},
  {{95.53571428571429`, 100.`}, {100.`, 76.19047619047619`}, 0.9609375`}}},
{{"Dbw", "Dsex", "Dage", "Kiddismax", "Kiddis内", "RCTaround",
  "AFAaround", "Subcutmax", "TP01", "Alb01", "TC01"}, {{107, 5, 0, 16},
  {{95.53571428571429`, 100.`}, {100.`, 76.19047619047619`}, 0.9609375`}}},
{{"Dbw", "Dsex", "artery", "Kiddismax", "Kiddis内", "Kiddis外",
  "RCTaround", "AFAaround", "TP01", "TG01", "TC01"}, {{107, 5, 0, 16},
  {{95.53571428571429`, 100.`}, {100.`, 76.19047619047619`}, 0.9609375`}}},
{{"Dbw", "Dage", "artery", "Kiddismax", "Kiddis内", "Kiddis外",
  "RCTaround", "AFAaround", "Subcutmax", "TG01", "TC01"}, {{107, 5, 0, 16},
  {{95.53571428571429`, 100.`}, {100.`, 76.19047619047619`}, 0.9609375`}}},
```

```
{{"Dbw", "Dage", "artery", "Kiddismax", "Kiddis内", "Kiddis外",
  "RCTaround", "AFAaround", "TP01", "TG01", "TC01"}, {{107, 5, 0, 16},
  {{95.53571428571429`, 100.`}, {100.`, 76.19047619047619`}, 0.9609375`}}},
{{"Dbw", "Dage", "artery", "Kiddismax", "Kiddis外", "RCTaround",
  "AFAaround", "Subcutmax", "TP01", "Alb01", "TC01"}, {{107, 5, 0, 16},
  {{95.53571428571429`, 100.`}, {100.`, 76.19047619047619`}, 0.9609375`}}},
{{"Dbw", "Dage", "artery", "Kiddismax", "Kiddis外", "RCTaround",
  "AFAaround", "Subcutmax", "TP01", "TG01", "TC01"}, {{107, 5, 0, 16},
  {{95.53571428571429`, 100.`}, {100.`, 76.19047619047619`}, 0.9609375`}}},
{{"Dbw", "Dage", "artery", "Kiddismax", "Kiddis外", "RCTaround",
  "AFAaround", "Subcutmax", "Alb01", "TG01", "TC01"}, {{107, 5, 0, 16},
  {{95.53571428571429`, 100.`}, {100.`, 76.19047619047619`}, 0.9609375`}}},
{{"Dbw", "Dage", "artery", "Kiddismax", "Kiddis外", "RCTaround",
  "AFAaround", "TP01", "Alb01", "TG01", "TC01"}, {{107, 5, 0, 16},
  {{95.53571428571429`, 100.`}, {100.`, 76.19047619047619`}, 0.9609375`}}},
{{"Dbw", "Dage", "artery", "Kiddismax", "RCTaround", "AFAaround",
  "Subcutmax", "TP01", "Alb01", "TG01", "TC01"}, {{107, 5, 0, 16},
  {{95.53571428571429`, 100.`}, {100.`, 76.19047619047619`}, 0.9609375`}}},
{{"Dbw", "Dage", "Kiddismax", "Kiddis内", "Kiddis外", "RCTaround",
  "AFAaround", "Subcutmax", "TP01", "Alb01", "TC01"}, {{107, 5, 0, 16},
  {{95.53571428571429`, 100.`}, {100.`, 76.19047619047619`}, 0.9609375`}}},
{{"Dbw", "Dage", "Kiddismax", "Kiddis内", "Kiddis外", "RCTaround",
  "AFAaround", "Subcutmax", "TP01", "TG01", "TC01"}, {{107, 5, 0, 16},
  {{95.53571428571429`, 100.`}, {100.`, 76.19047619047619`}, 0.9609375`}}},
{{"Dbw", "Dage", "Kiddismax", "Kiddis内", "Kiddis外", "RCTaround",
  "AFAaround", "Subcutmax", "Alb01", "TG01", "TC01"}, {{107, 5, 0, 16},
  {{95.53571428571429`, 100.`}, {100.`, 76.19047619047619`}, 0.9609375`}}},
{{"Dbw", "Dage", "Kiddismax", "Kiddis内", "Kiddis外", "RCTaround",
  "AFAaround", "TP01", "Alb01", "TG01", "TC01"}, {{107, 5, 0, 16},
  {{95.53571428571429`, 100.`}, {100.`, 76.19047619047619`}, 0.9609375`}}},
{{"Dbw", "Dage", "Kiddismax", "Kiddis内", "Kiddis外", "AFAaround",
  "Subcutmax", "TP01", "Alb01", "TG01", "TC01"}, {{107, 5, 0, 16},
  {{95.53571428571429`, 100.`}, {100.`, 76.19047619047619`}, 0.9609375`}}},
{{"Dbw", "Dage", "Kiddismax", "Kiddis内", "RCTaround", "AFAaround",
  "Subcutmax", "TP01", "Alb01", "TG01", "TC01"}, {{107, 5, 0, 16},
  {{95.53571428571429`, 100.`}, {100.`, 76.19047619047619`}, 0.9609375`}}},
{{"Dbw", "artery", "Kiddismax", "Kiddis内", "Kiddis外", "RCTaround",
  "AFAaround", "Subcutmax", "TP01", "TG01", "TC01"}, {{107, 5, 0, 16},
  {{95.53571428571429`, 100.`}, {100.`, 76.19047619047619`}, 0.9609375`}}},
{{"Dbw", "artery", "Kiddismax", "Kiddis内", "Kiddis外", "RCTaround",
  "AFAaround", "Subcutmax", "Alb01", "TG01", "TC01"}, {{107, 5, 0, 16},
  {{95.53571428571429`, 100.`}, {100.`, 76.19047619047619`}, 0.9609375`}}},
{{"Dbw", "artery", "Kiddismax", "Kiddis内", "Kiddis外", "RCTaround",
  "AFAaround", "TP01", "Alb01", "TG01", "TC01"}, {{107, 5, 0, 16},
  {{95.53571428571429`, 100.`}, {100.`, 76.19047619047619`}, 0.9609375`}}},
```

```
{{"Dbw", "artery", "Kiddismax", "Kiddis外", "RCTaround", "AFAaround",
  "Subcutmax", "TP01", "Alb01", "TG01", "TC01"}, {{107, 5, 0, 16},
  {{95.53571428571429`, 100.`}, {100.`, 76.19047619047619`}, 0.9609375`}}},
{{"Dbw", "Kiddismax", "Kiddis内", "Kiddis外", "RCTaround", "AFAaround",
  "Subcutmax", "TP01", "Alb01", "TG01", "TC01"}, {{107, 5, 0, 16},
  {{95.53571428571429`, 100.`}, {100.`, 76.19047619047619`}, 0.9609375`}}},
{{"Dsex", "Dage", "artery", "Kiddismax", "Kiddis外", "RCTaround",
  "AFAaround", "Subcutmax", "TP01", "Alb01", "TC01"}, {{107, 5, 0, 16},
  {{95.53571428571429`, 100.`}, {100.`, 76.19047619047619`}, 0.9609375`}}},
{{"Dsex", "Dage", "artery", "Kiddismax", "Kiddis外", "RCTaround",
  "AFAaround", "Subcutmax", "TP01", "TG01", "TC01"}, {{107, 5, 0, 16},
  {{95.53571428571429`, 100.`}, {100.`, 76.19047619047619`}, 0.9609375`}}},
{{"Dsex", "Dage", "artery", "Kiddismax", "Kiddis外", "RCTaround",
  "AFAaround", "Subcutmax", "Alb01", "TG01", "TC01"}, {{107, 5, 0, 16},
  {{95.53571428571429`, 100.`}, {100.`, 76.19047619047619`}, 0.9609375`}}},
{{"Dsex", "Dage", "artery", "Kiddismax", "Kiddis外", "RCTaround",
  "AFAaround", "TP01", "Alb01", "TG01", "TC01"}, {{107, 5, 0, 16},
  {{95.53571428571429`, 100.`}, {100.`, 76.19047619047619`}, 0.9609375`}}},
{{"Dsex", "Dage", "artery", "Kiddismax", "Kiddis外", "AFAaround",
  "Subcutmax", "TP01", "Alb01", "TG01", "TC01"}, {{107, 5, 0, 16},
  {{95.53571428571429`, 100.`}, {100.`, 76.19047619047619`}, 0.9609375`}}},
{{"Dsex", "Dage", "artery", "Kiddismax", "RCTaround", "AFAaround",
  "Subcutmax", "TP01", "Alb01", "TG01", "TC01"}, {{107, 5, 0, 16},
  {{95.53571428571429`, 100.`}, {100.`, 76.19047619047619`}, 0.9609375`}}},
{{"Dsex", "Dage", "Kiddismax", "Kiddis内", "Kiddis外", "RCTaround",
  "AFAaround", "Subcutmax", "TP01", "Alb01", "TC01"}, {{107, 5, 0, 16},
  {{95.53571428571429`, 100.`}, {100.`, 76.19047619047619`}, 0.9609375`}}},
{{"Dsex", "Dage", "Kiddismax", "Kiddis内", "RCTaround", "AFAaround",
  "Subcutmax", "TP01", "Alb01", "TG01", "TC01"}, {{107, 5, 0, 16},
  {{95.53571428571429`, 100.`}, {100.`, 76.19047619047619`}, 0.9609375`}}},
{{"Dsex", "artery", "Kiddismax", "Kiddis内", "Kiddis外", "RCTaround",
  "AFAaround", "Subcutmax", "TP01", "TG01", "TC01"}, {{107, 5, 0, 16},
  {{95.53571428571429`, 100.`}, {100.`, 76.19047619047619`}, 0.9609375`}}},
{{"Dsex", "artery", "Kiddismax", "Kiddis内", "Kiddis外", "RCTaround",
  "AFAaround", "TP01", "Alb01", "TG01", "TC01"}, {{107, 5, 0, 16},
  {{95.53571428571429`, 100.`}, {100.`, 76.19047619047619`}, 0.9609375`}}},
{{"Dsex", "Kiddismax", "Kiddis内", "Kiddis外", "RCTaround", "AFAaround",
  "Subcutmax", "TP01", "Alb01", "TG01", "TC01"}, {{107, 5, 0, 16},
  {{95.53571428571429`, 100.`}, {100.`, 76.19047619047619`}, 0.9609375`}}},
{{"Dage", "artery", "Kiddismax", "Kiddis内", "Kiddis外", "RFAaround",
  "RCTaround", "AFAaround", "Subcutmax", "TP01", "TC01"}, {{107, 5, 0, 16},
  {{95.53571428571429`, 100.`}, {100.`, 76.19047619047619`}, 0.9609375`}}},
{{"Dage", "artery", "Kiddismax", "Kiddis内", "Kiddis外", "RFAaround",
  "RCTaround", "AFAaround", "Subcutmax", "Alb01", "TC01"}, {{107, 5, 0, 16},
  {{95.53571428571429`, 100.`}, {100.`, 76.19047619047619`}, 0.9609375`}}},
```

```
{{"Dage", "artery", "Kiddismax", "Kiddis内", "Kiddis外", "RFAaround",
  "RCTaround", "AFAaround", "Subcutmax", "TG01", "TC01"}, {{107, 5, 0, 16},
  {{95.53571428571429`, 100.`}, {100.`, 76.19047619047619`}, 0.9609375`}}},
{{"Dage", "artery", "Kiddismax", "Kiddis内", "Kiddis外", "RFAaround",
  "RCTaround", "AFAaround", "TP01", "Alb01", "TC01"}, {{107, 5, 0, 16},
  {{95.53571428571429`, 100.`}, {100.`, 76.19047619047619`}, 0.9609375`}}},
{{"Dage", "artery", "Kiddismax", "Kiddis内", "Kiddis外", "RFAaround",
  "RCTaround", "AFAaround", "TP01", "TG01", "TC01"}, {{107, 5, 0, 16},
  {{95.53571428571429`, 100.`}, {100.`, 76.19047619047619`}, 0.9609375`}}},
{{"Dage", "artery", "Kiddismax", "Kiddis内", "Kiddis外", "RFAaround",
  "RCTaround", "Subcutmax", "TP01", "TG01", "TC01"}, {{107, 5, 0, 16},
  {{95.53571428571429`, 100.`}, {100.`, 76.19047619047619`}, 0.9609375`}}},
{{"Dage", "artery", "Kiddismax", "Kiddis内", "Kiddis外", "RCTaround",
  "AFAaround", "Subcutmax", "TP01", "Alb01", "TC01"}, {{107, 5, 0, 16},
  {{95.53571428571429`, 100.`}, {100.`, 76.19047619047619`}, 0.9609375`}}},
{{"Dage", "artery", "Kiddismax", "Kiddis内", "Kiddis外", "RCTaround",
  "AFAaround", "Subcutmax", "TP01", "TG01", "TC01"}, {{107, 5, 0, 16},
  {{95.53571428571429`, 100.`}, {100.`, 76.19047619047619`}, 0.9609375`}}},
{{"Dage", "artery", "Kiddismax", "Kiddis内", "Kiddis外", "RCTaround",
  "AFAaround", "Subcutmax", "Alb01", "TG01", "TC01"}, {{107, 5, 0, 16},
  {{95.53571428571429`, 100.`}, {100.`, 76.19047619047619`}, 0.9609375`}}},
{{"Dage", "artery", "Kiddismax", "Kiddis内", "RFAaround", "RCTaround",
  "AFAaround", "Subcutmax", "TP01", "TG01", "TC01"}, {{107, 5, 0, 16},
  {{95.53571428571429`, 100.`}, {100.`, 76.19047619047619`}, 0.9609375`}}},
{{"Dage", "artery", "Kiddismax", "Kiddis内", "RFAaround", "RCTaround",
  "AFAaround", "Subcutmax", "Alb01", "TG01", "TC01"}, {{107, 5, 0, 16},
  {{95.53571428571429`, 100.`}, {100.`, 76.19047619047619`}, 0.9609375`}}},
{{"Dage", "artery", "Kiddismax", "Kiddis内", "RFAaround", "RCTaround",
  "AFAaround", "TP01", "Alb01", "TG01", "TC01"}, {{107, 5, 0, 16},
  {{95.53571428571429`, 100.`}, {100.`, 76.19047619047619`}, 0.9609375`}}},
{{"Dage", "artery", "Kiddismax", "Kiddis外", "RCTaround", "AFAaround",
  "Subcutmax", "TP01", "Alb01", "TG01", "TC01"}, {{107, 5, 0, 16},
  {{95.53571428571429`, 100.`}, {100.`, 76.19047619047619`}, 0.9609375`}}},
{{"Dage", "Kiddismax", "Kiddis内", "Kiddis外", "RCTaround", "AFAaround",
  "Subcutmax", "TP01", "Alb01", "TG01", "TC01"}, {{107, 5, 0, 16},
  {{95.53571428571429`, 100.`}, {100.`, 76.19047619047619`}, 0.9609375`}}},
{{"artery", "Kiddismax", "Kiddis内", "Kiddis外", "RFAaround", "RCTaround",
  "AFAaround", "TP01", "Alb01", "TG01", "TC01"}, {{107, 5, 0, 16},
  {{95.53571428571429`, 100.`}, {100.`, 76.19047619047619`}, 0.9609375`}}},
{{"artery", "Kiddismax", "Kiddis内", "Kiddis外", "RCTaround", "AFAaround",
  "Subcutmax", "TP01", "Alb01", "TG01", "TC01"}, {{107, 5, 0, 16},
  {{95.53571428571429`, 100.`}, {100.`, 76.19047619047619`}, 0.9609375`}}},
{{"Dht", "Dbw", "Dsex", "Dage", "artery", "Kiddismax", "Kiddis内",
  "RCTaround", "AFAaround", "TP01", "TG01", "TC01"}, {{107, 5, 0, 16},
  {{95.53571428571429`, 100.`}, {100.`, 76.19047619047619`}, 0.9609375`}}},
```

```
{{"Dht", "Dbw", "Dsex", "Dage", "artery", "Kiddismax", "Kiddis外",
  "RCTaround", "AFAaround", "Subcutmax", "TP01", "TC01"}, {{107, 5, 0, 16},
  {{95.53571428571429`, 100.`}, {100.`, 76.19047619047619`}, 0.9609375`}}},
{{"Dht", "Dbw", "Dsex", "Dage", "artery", "Kiddismax", "Kiddis外",
  "RCTaround", "AFAaround", "TP01", "Alb01", "TC01"}, {{107, 5, 0, 16},
  {{95.53571428571429`, 100.`}, {100.`, 76.19047619047619`}, 0.9609375`}}},
{{"Dht", "Dbw", "Dsex", "Dage", "artery", "Kiddismax", "Kiddis外",
  "RCTaround", "AFAaround", "TP01", "TG01", "TC01"}, {{107, 5, 0, 16},
  {{95.53571428571429`, 100.`}, {100.`, 76.19047619047619`}, 0.9609375`}}},
{{"Dht", "Dbw", "Dsex", "Dage", "artery", "Kiddismax", "Kiddis外",
  "RCTaround", "AFAaround", "Alb01", "TG01", "TC01"}, {{107, 5, 0, 16},
  {{95.53571428571429`, 100.`}, {100.`, 76.19047619047619`}, 0.9609375`}}},
{{"Dht", "Dbw", "Dsex", "Dage", "artery", "Kiddismax", "Kiddis外",
  "AFAaround", "TP01", "Alb01", "TG01", "TC01"}, {{107, 5, 0, 16},
  {{95.53571428571429`, 100.`}, {100.`, 76.19047619047619`}, 0.9609375`}}},
{{"Dht", "Dbw", "Dsex", "Dage", "artery", "Kiddismax", "RCTaround",
  "AFAaround", "TP01", "Alb01", "TG01", "TC01"}, {{107, 5, 0, 16},
  {{95.53571428571429`, 100.`}, {100.`, 76.19047619047619`}, 0.9609375`}}},
{{"Dht", "Dbw", "Dsex", "Dage", "Kiddismax", "Kiddis内", "Kiddis外",
  "RCTaround", "AFAaround", "Subcutmax", "TP01", "TC01"}, {{107, 5, 0, 16},
  {{95.53571428571429`, 100.`}, {100.`, 76.19047619047619`}, 0.9609375`}}},
{{"Dht", "Dbw", "Dsex", "Dage", "Kiddismax", "Kiddis内", "Kiddis外",
  "RCTaround", "AFAaround", "Subcutmax", "Alb01", "TC01"}, {{107, 5, 0, 16},
  {{95.53571428571429`, 100.`}, {100.`, 76.19047619047619`}, 0.9609375`}}},
{{"Dht", "Dbw", "Dsex", "Dage", "Kiddismax", "Kiddis内", "Kiddis外",
  "RCTaround", "AFAaround", "TP01", "Alb01", "TC01"}, {{107, 5, 0, 16},
  {{95.53571428571429`, 100.`}, {100.`, 76.19047619047619`}, 0.9609375`}}},
{{"Dht", "Dbw", "Dsex", "Dage", "Kiddismax", "Kiddis内", "Kiddis外",
  "AFAaround", "Subcutmax", "TP01", "Alb01", "TC01"}, {{107, 5, 0, 16},
  {{95.53571428571429`, 100.`}, {100.`, 76.19047619047619`}, 0.9609375`}}},
{{"Dht", "Dbw", "Dsex", "Dage", "Kiddismax", "Kiddis内", "RCTaround",
  "AFAaround", "Subcutmax", "TP01", "Alb01", "TC01"}, {{107, 5, 0, 16},
  {{95.53571428571429`, 100.`}, {100.`, 76.19047619047619`}, 0.9609375`}}},
{{"Dht", "Dbw", "Dsex", "Kiddismax", "Kiddis内", "Kiddis外", "RCTaround",
  "AFAaround", "Subcutmax", "TP01", "Alb01", "TC01"}, {{107, 5, 0, 16},
  {{95.53571428571429`, 100.`}, {100.`, 76.19047619047619`}, 0.9609375`}}},
{{"Dht", "Dbw", "Dage", "artery", "Kiddismax", "Kiddis内", "Kiddis外",
  "RCTaround", "AFAaround", "Subcutmax", "TG01", "TC01"}, {{107, 5, 0, 16},
  {{95.53571428571429`, 100.`}, {100.`, 76.19047619047619`}, 0.9609375`}}},
{{"Dht", "Dbw", "Dage", "artery", "Kiddismax", "Kiddis内", "Kiddis外",
  "RCTaround", "AFAaround", "TP01", "TG01", "TC01"}, {{107, 5, 0, 16},
  {{95.53571428571429`, 100.`}, {100.`, 76.19047619047619`}, 0.9609375`}}},
{{"Dht", "Dbw", "Dage", "artery", "Kiddismax", "Kiddis外", "RCTaround",
  "AFAaround", "Subcutmax", "TP01", "Alb01", "TC01"}, {{107, 5, 0, 16},
  {{95.53571428571429`, 100.`}, {100.`, 76.19047619047619`}, 0.9609375`}}},
```

```
{{"Dht", "Dbw", "Dage", "artery", "Kiddismax", "Kiddis外", "RCTaround",
  "AFAaround", "Subcutmax", "TP01", "TG01", "TC01"}, {{107, 5, 0, 16},
  {{95.53571428571429`, 100.`}, {100.`, 76.19047619047619`}, 0.9609375`}}},
{{"Dht", "Dbw", "Dage", "artery", "Kiddismax", "Kiddis外", "RCTaround",
  "AFAaround", "Subcutmax", "Alb01", "TG01", "TC01"}, {{107, 5, 0, 16},
  {{95.53571428571429`, 100.`}, {100.`, 76.19047619047619`}, 0.9609375`}}},
{{"Dht", "Dbw", "Dage", "artery", "Kiddismax", "Kiddis外", "RCTaround",
  "AFAaround", "TP01", "Alb01", "TG01", "TC01"}, {{107, 5, 0, 16},
  {{95.53571428571429`, 100.`}, {100.`, 76.19047619047619`}, 0.9609375`}}},
{{"Dht", "Dbw", "Dage", "artery", "Kiddismax", "RCTaround", "AFAaround",
  "Subcutmax", "TP01", "Alb01", "TG01", "TC01"}, {{107, 5, 0, 16},
  {{95.53571428571429`, 100.`}, {100.`, 76.19047619047619`}, 0.9609375`}}},
{{"Dht", "Dbw", "Dage", "Kiddismax", "Kiddis内", "Kiddis外", "RCTaround",
  "AFAaround", "Subcutmax", "TP01", "Alb01", "TC01"}, {{107, 5, 0, 16},
  {{95.53571428571429`, 100.`}, {100.`, 76.19047619047619`}, 0.9609375`}}},
{{"Dht", "Dbw", "Dage", "Kiddismax", "Kiddis内", "Kiddis外", "RCTaround",
  "AFAaround", "Subcutmax", "TP01", "TG01", "TC01"}, {{107, 5, 0, 16},
  {{95.53571428571429`, 100.`}, {100.`, 76.19047619047619`}, 0.9609375`}}},
{{"Dht", "Dbw", "Dage", "Kiddismax", "Kiddis内", "Kiddis外", "RCTaround",
  "AFAaround", "Subcutmax", "Alb01", "TG01", "TC01"}, {{107, 5, 0, 16},
  {{95.53571428571429`, 100.`}, {100.`, 76.19047619047619`}, 0.9609375`}}},
{{"Dht", "Dbw", "Dage", "Kiddismax", "Kiddis内", "Kiddis外", "RCTaround",
  "AFAaround", "TP01", "Alb01", "TG01", "TC01"}, {{107, 5, 0, 16},
  {{95.53571428571429`, 100.`}, {100.`, 76.19047619047619`}, 0.9609375`}}},
{{"Dht", "Dbw", "Dage", "Kiddismax", "Kiddis内", "Kiddis外", "AFAaround",
  "Subcutmax", "TP01", "Alb01", "TG01", "TC01"}, {{107, 5, 0, 16},
  {{95.53571428571429`, 100.`}, {100.`, 76.19047619047619`}, 0.9609375`}}},
{{"Dht", "Dbw", "Dage", "Kiddismax", "Kiddis内", "RCTaround", "AFAaround",
  "Subcutmax", "TP01", "Alb01", "TG01", "TC01"}, {{107, 5, 0, 16},
  {{95.53571428571429`, 100.`}, {100.`, 76.19047619047619`}, 0.9609375`}}},
{{"Dht", "Dbw", "artery", "Kiddismax", "Kiddis内", "Kiddis外", "RCTaround",
  "AFAaround", "Subcutmax", "TP01", "TG01", "TC01"}, {{107, 5, 0, 16},
  {{95.53571428571429`, 100.`}, {100.`, 76.19047619047619`}, 0.9609375`}}},
{{"Dht", "Dbw", "artery", "Kiddismax", "Kiddis内", "Kiddis外", "RCTaround",
  "AFAaround", "Subcutmax", "Alb01", "TG01", "TC01"}, {{107, 5, 0, 16},
  {{95.53571428571429`, 100.`}, {100.`, 76.19047619047619`}, 0.9609375`}}},
{{"Dht", "Dbw", "artery", "Kiddismax", "Kiddis内", "Kiddis外", "RCTaround",
  "AFAaround", "TP01", "Alb01", "TG01", "TC01"}, {{107, 5, 0, 16},
  {{95.53571428571429`, 100.`}, {100.`, 76.19047619047619`}, 0.9609375`}}},
{{"Dht", "Dbw", "Kiddismax", "Kiddis内", "Kiddis外", "RCTaround",
  "AFAaround", "Subcutmax", "TP01", "Alb01", "TG01", "TC01"}, {{107, 5, 0, 16},
  {{95.53571428571429`, 100.`}, {100.`, 76.19047619047619`}, 0.9609375`}}},
{{"Dht", "Dsex", "Dage", "artery", "Kiddismax", "Kiddis外", "RCTaround",
  "AFAaround", "Subcutmax", "TP01", "Alb01", "TC01"}, {{107, 5, 0, 16},
  {{95.53571428571429`, 100.`}, {100.`, 76.19047619047619`}, 0.9609375`}}},
```

```
{{"Dht", "Dsex", "Dage", "artery", "Kiddismax", "Kiddis外", "RCTaround",
  "AFAaround", "Subcutmax", "TP01", "TG01", "TC01"}, {{107, 5, 0, 16},
  {{95.53571428571429`, 100.`}, {100.`, 76.19047619047619`}, 0.9609375`}}},
{{"Dht", "Dsex", "Dage", "artery", "Kiddismax", "Kiddis外", "RCTaround",
  "AFAaround", "Subcutmax", "Alb01", "TG01", "TC01"}, {{107, 5, 0, 16},
  {{95.53571428571429`, 100.`}, {100.`, 76.19047619047619`}, 0.9609375`}}},
{{"Dht", "Dsex", "Dage", "artery", "Kiddismax", "Kiddis外", "RCTaround",
  "AFAaround", "TP01", "Alb01", "TG01", "TC01"}, {{107, 5, 0, 16},
  {{95.53571428571429`, 100.`}, {100.`, 76.19047619047619`}, 0.9609375`}}},
{{"Dht", "Dsex", "Dage", "artery", "Kiddismax", "Kiddis外", "AFAaround",
  "Subcutmax", "TP01", "Alb01", "TG01", "TC01"}, {{107, 5, 0, 16},
  {{95.53571428571429`, 100.`}, {100.`, 76.19047619047619`}, 0.9609375`}}},
{{"Dht", "Dsex", "Dage", "artery", "Kiddismax", "RCTaround", "AFAaround",
  "Subcutmax", "TP01", "Alb01", "TG01", "TC01"}, {{107, 5, 0, 16},
  {{95.53571428571429`, 100.`}, {100.`, 76.19047619047619`}, 0.9609375`}}},
{{"Dht", "Dsex", "Dage", "Kiddismax", "Kiddis内", "Kiddis外", "RCTaround",
  "AFAaround", "Subcutmax", "TP01", "Alb01", "TC01"}, {{107, 5, 0, 16},
  {{95.53571428571429`, 100.`}, {100.`, 76.19047619047619`}, 0.9609375`}}},
{{"Dht", "Dsex", "Dage", "Kiddismax", "Kiddis内", "Kiddis外", "AFAaround",
  "Subcutmax", "TP01", "Alb01", "TG01", "TC01"}, {{107, 5, 0, 16},
  {{95.53571428571429`, 100.`}, {100.`, 76.19047619047619`}, 0.9609375`}}},
{{"Dht", "Dsex", "Dage", "Kiddismax", "Kiddis内", "RCTaround", "AFAaround",
  "Subcutmax", "TP01", "Alb01", "TG01", "TC01"}, {{107, 5, 0, 16},
  {{95.53571428571429`, 100.`}, {100.`, 76.19047619047619`}, 0.9609375`}}},
{{"Dht", "Dsex", "artery", "Kiddismax", "Kiddis内", "Kiddis外", "RCTaround",
  "AFAaround", "Subcutmax", "TP01", "TG01", "TC01"}, {{107, 5, 0, 16},
  {{95.53571428571429`, 100.`}, {100.`, 76.19047619047619`}, 0.9609375`}}},
{{"Dht", "Dsex", "artery", "Kiddismax", "Kiddis内", "Kiddis外",
  "RCTaround", "AFAaround", "TP01", "Alb01", "TG01", "TC01"}, {{107, 5, 0, 16},
  {{95.53571428571429`, 100.`}, {100.`, 76.19047619047619`}, 0.9609375`}}},
{{"Dht", "Dsex", "Kiddismax", "Kiddis内", "Kiddis外", "RCTaround",
  "AFAaround", "Subcutmax", "TP01", "Alb01", "TG01", "TC01"}, {{107, 5, 0, 16},
  {{95.53571428571429`, 100.`}, {100.`, 76.19047619047619`}, 0.9609375`}}},
{{"Dht", "Dage", "artery", "Kiddismax", "Kiddis内", "Kiddis外", "RCTaround",
  "AFAaround", "Subcutmax", "TP01", "TG01", "TC01"}, {{107, 5, 0, 16},
  {{95.53571428571429`, 100.`}, {100.`, 76.19047619047619`}, 0.9609375`}}},
{{"Dht", "Dage", "artery", "Kiddismax", "Kiddis内", "Kiddis外", "RCTaround",
  "AFAaround", "Subcutmax", "Alb01", "TG01", "TC01"}, {{107, 5, 0, 16},
  {{95.53571428571429`, 100.`}, {100.`, 76.19047619047619`}, 0.9609375`}}},
{{"Dht", "Dage", "artery", "Kiddismax", "Kiddis外", "RCTaround",
  "AFAaround", "Subcutmax", "TP01", "Alb01", "TG01", "TC01"}, {{107, 5, 0, 16},
  {{95.53571428571429`, 100.`}, {100.`, 76.19047619047619`}, 0.9609375`}}},
{{"Dht", "Dage", "Kiddismax", "Kiddis内", "Kiddis外", "RCTaround",
  "AFAaround", "Subcutmax", "TP01", "Alb01", "TG01", "TC01"}, {{107, 5, 0, 16},
  {{95.53571428571429`, 100.`}, {100.`, 76.19047619047619`}, 0.9609375`}}},
```

```
{{"Dht", "artery", "Kiddismax", "Kiddis内", "Kiddis外", "RCTaround",
  "AFAaround", "Subcutmax", "TP01", "Alb01", "TG01", "TC01"}, {{107, 5, 0, 16},
  {{95.53571428571429`, 100.`}, {100.`, 76.19047619047619`}, 0.9609375`}}},
{{"Dbw", "Dsex", "Dage", "artery", "Kiddismax", "Kiddis外", "RCTaround",
  "AFAaround", "Subcutmax", "TP01", "Alb01", "TC01"}, {{107, 5, 0, 16},
  {{95.53571428571429`, 100.`}, {100.`, 76.19047619047619`}, 0.9609375`}}},
{{"Dbw", "Dsex", "Dage", "artery", "Kiddismax", "Kiddis外", "RCTaround",
  "AFAaround", "Subcutmax", "TP01", "TG01", "TC01"}, {{107, 5, 0, 16},
  {{95.53571428571429`, 100.`}, {100.`, 76.19047619047619`}, 0.9609375`}}},
{{"Dbw", "Dsex", "Dage", "artery", "Kiddismax", "Kiddis外", "RCTaround",
  "AFAaround", "Subcutmax", "Alb01", "TG01", "TC01"}, {{107, 5, 0, 16},
  {{95.53571428571429`, 100.`}, {100.`, 76.19047619047619`}, 0.9609375`}}},
{{"Dbw", "Dsex", "Dage", "artery", "Kiddismax", "Kiddis外", "RCTaround",
  "AFAaround", "TP01", "Alb01", "TG01", "TC01"}, {{107, 5, 0, 16},
  {{95.53571428571429`, 100.`}, {100.`, 76.19047619047619`}, 0.9609375`}}},
{{"Dbw", "Dsex", "Dage", "artery", "Kiddismax", "RCTaround", "AFAaround",
  "Subcutmax", "TP01", "Alb01", "TG01", "TC01"}, {{107, 5, 0, 16},
  {{95.53571428571429`, 100.`}, {100.`, 76.19047619047619`}, 0.9609375`}}},
{{"Dbw", "Dsex", "Dage", "Kiddismax", "Kiddis内", "Kiddis外", "RCTaround",
  "AFAaround", "Subcutmax", "TP01", "Alb01", "TC01"}, {{107, 5, 0, 16},
  {{95.53571428571429`, 100.`}, {100.`, 76.19047619047619`}, 0.9609375`}}},
{{"Dbw", "Dsex", "Dage", "Kiddismax", "Kiddis内", "Kiddis外", "RCTaround",
  "AFAaround", "Subcutmax", "TP01", "TG01", "TC01"}, {{107, 5, 0, 16},
  {{95.53571428571429`, 100.`}, {100.`, 76.19047619047619`}, 0.9609375`}}},
{{"Dbw", "Dsex", "Dage", "Kiddismax", "Kiddis内", "Kiddis外", "RCTaround",
  "AFAaround", "TP01", "Alb01", "TG01", "TC01"}, {{107, 5, 0, 16},
  {{95.53571428571429`, 100.`}, {100.`, 76.19047619047619`}, 0.9609375`}}},
{{"Dbw", "Dsex", "Dage", "Kiddismax", "Kiddis内", "Kiddis外", "AFAaround",
  "Subcutmax", "TP01", "Alb01", "TG01", "TC01"}, {{107, 5, 0, 16},
  {{95.53571428571429`, 100.`}, {100.`, 76.19047619047619`}, 0.9609375`}}},
{{"Dbw", "Dsex", "Dage", "Kiddismax", "Kiddis内", "RCTaround", "AFAaround",
  "Subcutmax", "TP01", "Alb01", "TG01", "TC01"}, {{107, 5, 0, 16},
  {{95.53571428571429`, 100.`}, {100.`, 76.19047619047619`}, 0.9609375`}}},
{{"Dbw", "Dsex", "artery", "Kiddismax", "Kiddis内", "Kiddis外", "RCTaround",
  "AFAaround", "Subcutmax", "TP01", "TG01", "TC01"}, {{107, 5, 0, 16},
  {{95.53571428571429`, 100.`}, {100.`, 76.19047619047619`}, 0.9609375`}}},
{{"Dbw", "Dsex", "artery", "Kiddismax", "Kiddis内", "Kiddis外",
  "RCTaround", "AFAaround", "TP01", "Alb01", "TG01", "TC01"}, {{107, 5, 0, 16},
  {{95.53571428571429`, 100.`}, {100.`, 76.19047619047619`}, 0.9609375`}}},
{{"Dbw", "Dage", "artery", "Kiddismax", "Kiddis内", "Kiddis外", "RCTaround",
  "AFAaround", "Subcutmax", "TP01", "TG01", "TC01"}, {{107, 5, 0, 16},
  {{95.53571428571429`, 100.`}, {100.`, 76.19047619047619`}, 0.9609375`}}},
{{"Dbw", "Dage", "artery", "Kiddismax", "Kiddis内", "Kiddis外",
  "RCTaround", "AFAaround", "TP01", "Alb01", "TG01", "TC01"}, {{107, 5, 0, 16},
  {{95.53571428571429`, 100.`}, {100.`, 76.19047619047619`}, 0.9609375`}}},
```

```
{{"Dbw", "Dage", "artery", "Kiddismax", "Kiddis外", "RCTaround",
  "AFAaround", "Subcutmax", "TP01", "Alb01", "TG01", "TC01"}, {{107, 5, 0, 16},
  {{95.53571428571429`, 100.`}, {100.`, 76.19047619047619`}, 0.9609375`}}},
{{"Dbw", "Dage", "Kiddismax", "Kiddis内", "Kiddis外", "RCTaround",
  "AFAaround", "Subcutmax", "TP01", "Alb01", "TG01", "TC01"}, {{107, 5, 0, 16},
  {{95.53571428571429`, 100.`}, {100.`, 76.19047619047619`}, 0.9609375`}}},
{{"Dbw", "artery", "Kiddismax", "Kiddis内", "Kiddis外", "RCTaround",
  "AFAaround", "Subcutmax", "TP01", "Alb01", "TG01", "TC01"}, {{107, 5, 0, 16},
  {{95.53571428571429`, 100.`}, {100.`, 76.19047619047619`}, 0.9609375`}}},
{{"Dsex", "Dage", "artery", "Kiddismax", "Kiddis内", "Kiddis外", "RCTaround",
  "AFAaround", "Subcutmax", "TP01", "TG01", "TC01"}, {{107, 5, 0, 16},
  {{95.53571428571429`, 100.`}, {100.`, 76.19047619047619`}, 0.9609375`}}},
{{"Dsex", "Dage", "artery", "Kiddismax", "Kiddis内", "Kiddis外", "RCTaround",
  "AFAaround", "Subcutmax", "Alb01", "TG01", "TC01"}, {{107, 5, 0, 16},
  {{95.53571428571429`, 100.`}, {100.`, 76.19047619047619`}, 0.9609375`}}},
{{"Dsex", "Dage", "artery", "Kiddismax", "Kiddis外", "RCTaround",
  "AFAaround", "Subcutmax", "TP01", "Alb01", "TG01", "TC01"}, {{107, 5, 0, 16},
  {{95.53571428571429`, 100.`}, {100.`, 76.19047619047619`}, 0.9609375`}}},
{{"Dsex", "Dage", "Kiddismax", "Kiddis内", "Kiddis外", "RCTaround",
  "AFAaround", "Subcutmax", "TP01", "Alb01", "TG01", "TC01"}, {{107, 5, 0, 16},
  {{95.53571428571429`, 100.`}, {100.`, 76.19047619047619`}, 0.9609375`}}},
{{"Dsex", "artery", "Kiddismax", "Kiddis内", "Kiddis外", "RCTaround",
  "AFAaround", "Subcutmax", "TP01", "Alb01", "TG01", "TC01"}, {{107, 5, 0, 16},
  {{95.53571428571429`, 100.`}, {100.`, 76.19047619047619`}, 0.9609375`}}},
{{"Dage", "artery", "Kiddismax", "Kiddis内", "Kiddis外",
  "RFAaround", "RCTaround", "AFAaround", "Subcutmax",
  "Alb01", "TG01", "TC01"}, {{107, 5, 0, 16},
  {{95.53571428571429`, 100.`}, {100.`, 76.19047619047619`}, 0.9609375`}}},
{{"Dage", "artery", "Kiddismax", "Kiddis内", "Kiddis外", "RCTaround",
  "AFAaround", "Subcutmax", "TP01", "Alb01", "TG01", "TC01"}, {{107, 5, 0, 16},
  {{95.53571428571429`, 100.`}, {100.`, 76.19047619047619`}, 0.9609375`}}},
{{"Dht", "Dbw", "Dsex", "Dage", "artery", "Kiddismax", "Kiddis外", "RCTaround",
  "AFAaround", "Subcutmax", "TP01", "Alb01", "TC01"}, {{107, 5, 0, 16},
  {{95.53571428571429`, 100.`}, {100.`, 76.19047619047619`}, 0.9609375`}}},
{{"Dht", "Dbw", "Dsex", "Dage", "artery", "Kiddismax", "Kiddis外", "RCTaround",
  "AFAaround", "Subcutmax", "TP01", "TG01", "TC01"}, {{107, 5, 0, 16},
  {{95.53571428571429`, 100.`}, {100.`, 76.19047619047619`}, 0.9609375`}}},
{{"Dht", "Dbw", "Dsex", "Dage", "artery", "Kiddismax", "Kiddis外",
  "RCTaround", "AFAaround", "TP01", "Alb01", "TG01", "TC01"}, {{107, 5, 0, 16},
  {{95.53571428571429`, 100.`}, {100.`, 76.19047619047619`}, 0.9609375`}}},
{{"Dht", "Dbw", "Dsex", "Dage", "Kiddismax", "Kiddis内", "Kiddis外",
  "RCTaround", "AFAaround", "Subcutmax", "TP01", "Alb01", "TC01"},
 {{107, 5, 0, 16}, {{95.53571428571429`, 100.`},
   {100.`, 76.19047619047619`}, 0.9609375`}}},
{{"Dht", "Dbw", "Dsex", "Dage", "Kiddismax", "Kiddis内", "Kiddis外",
```

```
  "RCTaround", "AFAaround", "Subcutmax", "TP01", "TG01", "TC01"},
 {{107, 5, 0, 16}, {{95.53571428571429`, 100.`},
   {100.`, 76.19047619047619`}, 0.9609375`}}},
{{"Dht", "Dbw", "Dsex", "Dage", "Kiddismax", "Kiddis内", "Kiddis外",
  "RCTaround", "AFAaround", "Subcutmax", "Alb01", "TG01", "TC01"},
 {{107, 5, 0, 16}, {{95.53571428571429`, 100.`},
   {100.`, 76.19047619047619`}, 0.9609375`}}},
{{"Dht", "Dbw", "Dsex", "Dage", "Kiddismax", "Kiddis内", "Kiddis外",
  "RCTaround", "AFAaround", "TP01", "Alb01", "TG01", "TC01"}, {{107, 5, 0, 16},
  {{95.53571428571429`, 100.`}, {100.`, 76.19047619047619`}, 0.9609375`}}},
{{"Dht", "Dbw", "Dsex", "Dage", "Kiddismax", "Kiddis内", "Kiddis外",
  "AFAaround", "Subcutmax", "TP01", "Alb01", "TG01", "TC01"}, {{107, 5, 0, 16},
  {{95.53571428571429`, 100.`}, {100.`, 76.19047619047619`}, 0.9609375`}}},
{{"Dht", "Dbw", "Dsex", "Dage", "Kiddismax", "Kiddis内", "RCTaround",
  "AFAaround", "Subcutmax", "TP01", "Alb01", "TG01", "TC01"}, {{107, 5, 0, 16},
  {{95.53571428571429`, 100.`}, {100.`, 76.19047619047619`}, 0.9609375`}}},
{{"Dht", "Dbw", "Dsex", "artery", "Kiddismax", "Kiddis内",
  "Kiddis外", "RCTaround", "AFAaround", "Subcutmax",
  "TP01", "TG01", "TC01"}, {{107, 5, 0, 16},
  {{95.53571428571429`, 100.`}, {100.`, 76.19047619047619`}, 0.9609375`}}},
{{"Dht", "Dbw", "Dsex", "artery", "Kiddismax", "Kiddis内", "Kiddis外",
  "RCTaround", "AFAaround", "TP01", "Alb01", "TG01", "TC01"}, {{107, 5, 0, 16},
  {{95.53571428571429`, 100.`}, {100.`, 76.19047619047619`}, 0.9609375`}}},
{{"Dht", "Dbw", "Dsex", "Kiddismax", "Kiddis内", "Kiddis外", "RCTaround",
  "AFAaround", "Subcutmax", "TP01", "Alb01", "TG01", "TC01"}, {{107, 5, 0, 16},
  {{95.53571428571429`, 100.`}, {100.`, 76.19047619047619`}, 0.9609375`}}},
{{"Dht", "Dbw", "Dage", "artery", "Kiddismax", "Kiddis内",
  "Kiddis外", "RCTaround", "AFAaround", "Subcutmax",
  "TP01", "TG01", "TC01"}, {{107, 5, 0, 16},
  {{95.53571428571429`, 100.`}, {100.`, 76.19047619047619`}, 0.9609375`}}},
{{"Dht", "Dbw", "Dage", "artery", "Kiddismax", "Kiddis内", "Kiddis外",
  "RCTaround", "AFAaround", "TP01", "Alb01", "TG01", "TC01"}, {{107, 5, 0, 16},
  {{95.53571428571429`, 100.`}, {100.`, 76.19047619047619`}, 0.9609375`}}},
{{"Dht", "Dbw", "Dage", "artery", "Kiddismax", "Kiddis外", "RCTaround",
  "AFAaround", "Subcutmax", "TP01", "Alb01", "TG01", "TC01"}, {{107, 5, 0, 16},
  {{95.53571428571429`, 100.`}, {100.`, 76.19047619047619`}, 0.9609375`}}},
{{"Dht", "Dbw", "Dage", "Kiddismax", "Kiddis内", "Kiddis外", "RCTaround",
  "AFAaround", "Subcutmax", "TP01", "Alb01", "TG01", "TC01"}, {{107, 5, 0, 16},
  {{95.53571428571429`, 100.`}, {100.`, 76.19047619047619`}, 0.9609375`}}},
{{"Dht", "Dbw", "artery", "Kiddismax", "Kiddis内", "Kiddis外", "RCTaround",
  "AFAaround", "Subcutmax", "TP01", "Alb01", "TG01", "TC01"}, {{107, 5, 0, 16},
  {{95.53571428571429`, 100.`}, {100.`, 76.19047619047619`}, 0.9609375`}}},
{{"Dht", "Dsex", "Dage", "artery", "Kiddismax", "Kiddis内",
  "Kiddis外", "RCTaround", "AFAaround", "Subcutmax",
  "TP01", "TG01", "TC01"}, {{107, 5, 0, 16},
```

```
  {{95.53571428571429`, 100.`}, {100.`, 76.19047619047619`}, 0.9609375`}}},
{{"Dht", "Dsex", "Dage", "artery", "Kiddismax", "Kiddis内",
  "Kiddis外", "RCTaround", "AFAaround", "Subcutmax",
  "Alb01", "TG01", "TC01"}, {{107, 5, 0, 16},
  {{95.53571428571429`, 100.`}, {100.`, 76.19047619047619`}, 0.9609375`}}},
{{"Dht", "Dsex", "Dage", "artery", "Kiddismax", "Kiddis外", "RCTaround",
  "AFAaround", "Subcutmax", "TP01", "Alb01", "TG01", "TC01"}, {{107, 5, 0, 16},
  {{95.53571428571429`, 100.`}, {100.`, 76.19047619047619`}, 0.9609375`}}},
{{"Dht", "Dsex", "Dage", "Kiddismax", "Kiddis内", "Kiddis外", "RCTaround",
  "AFAaround", "Subcutmax", "TP01", "Alb01", "TG01", "TC01"}, {{107, 5, 0, 16},
  {{95.53571428571429`, 100.`}, {100.`, 76.19047619047619`}, 0.9609375`}}},
{{"Dht", "Dsex", "artery", "Kiddismax", "Kiddis内", "Kiddis外", "RCTaround",
  "AFAaround", "Subcutmax", "TP01", "Alb01", "TG01", "TC01"}, {{107, 5, 0, 16},
  {{95.53571428571429`, 100.`}, {100.`, 76.19047619047619`}, 0.9609375`}}},
{{"Dht", "Dage", "artery", "Kiddismax", "Kiddis内", "Kiddis外", "RCTaround",
  "AFAaround", "Subcutmax", "TP01", "Alb01", "TG01", "TC01"}, {{107, 5, 0, 16},
  {{95.53571428571429`, 100.`}, {100.`, 76.19047619047619`}, 0.9609375`}}},
{{"Dbw", "Dsex", "Dage", "artery", "Kiddismax", "Kiddis内",
  "Kiddis外", "RCTaround", "AFAaround", "Subcutmax",
  "TP01", "TG01", "TC01"}, {{107, 5, 0, 16},
  {{95.53571428571429`, 100.`}, {100.`, 76.19047619047619`}, 0.9609375`}}},
{{"Dbw", "Dsex", "Dage", "artery", "Kiddismax", "Kiddis内",
  "Kiddis外", "RCTaround", "AFAaround", "Subcutmax",
  "Alb01", "TG01", "TC01"}, {{107, 5, 0, 16},
  {{95.53571428571429`, 100.`}, {100.`, 76.19047619047619`}, 0.9609375`}}},
{{"Dbw", "Dsex", "Dage", "artery", "Kiddismax", "Kiddis外", "RCTaround",
  "AFAaround", "Subcutmax", "TP01", "Alb01", "TG01", "TC01"}, {{107, 5, 0, 16},
  {{95.53571428571429`, 100.`}, {100.`, 76.19047619047619`}, 0.9609375`}}},
{{"Dbw", "Dsex", "Dage", "Kiddismax", "Kiddis内", "Kiddis外", "RCTaround",
  "AFAaround", "Subcutmax", "TP01", "Alb01", "TG01", "TC01"}, {{107, 5, 0, 16},
  {{95.53571428571429`, 100.`}, {100.`, 76.19047619047619`}, 0.9609375`}}},
{{"Dbw", "Dsex", "artery", "Kiddismax", "Kiddis内", "Kiddis外", "RCTaround",
  "AFAaround", "Subcutmax", "TP01", "Alb01", "TG01", "TC01"}, {{107, 5, 0, 16},
  {{95.53571428571429`, 100.`}, {100.`, 76.19047619047619`}, 0.9609375`}}},
{{"Dbw", "Dage", "artery", "Kiddismax", "Kiddis内", "Kiddis外", "RCTaround",
  "AFAaround", "Subcutmax", "TP01", "Alb01", "TG01", "TC01"}, {{107, 5, 0, 16},
  {{95.53571428571429`, 100.`}, {100.`, 76.19047619047619`}, 0.9609375`}}},
{{"Dsex", "Dage", "artery", "Kiddismax", "Kiddis内", "Kiddis外", "RCTaround",
  "AFAaround", "Subcutmax", "TP01", "Alb01", "TG01", "TC01"}, {{107, 5, 0, 16},
  {{95.53571428571429`, 100.`}, {100.`, 76.19047619047619`}, 0.9609375`}}},
{{"Dht", "Dbw", "Dsex", "Dage", "artery", "Kiddismax",
  "Kiddis内", "Kiddis外", "RCTaround", "AFAaround",
  "Subcutmax", "TP01", "TG01", "TC01"}, {{107, 5, 0, 16},
  {{95.53571428571429`, 100.`}, {100.`, 76.19047619047619`}, 0.9609375`}}},
{{"Dht", "Dbw", "Dsex", "Dage", "artery", "Kiddismax", "Kiddis内",
```

```
  "Kiddis外", "RCTaround", "AFAaround", "Subcutmax",
  "Alb01", "TG01", "TC01"}, {{107, 5, 0, 16},
  {{95.53571428571429`, 100.`}, {100.`, 76.19047619047619`}, 0.9609375`}}},
{{"Dht", "Dbw", "Dsex", "Dage", "artery", "Kiddismax", "Kiddis内", "Kiddis外",
  "RCTaround", "AFAaround", "TP01", "Alb01", "TG01", "TC01"}, {{107, 5, 0, 16},
  {{95.53571428571429`, 100.`}, {100.`, 76.19047619047619`}, 0.9609375`}}},
{{"Dht", "Dbw", "Dsex", "Dage", "artery", "Kiddismax", "Kiddis外", "RCTaround",
  "AFAaround", "Subcutmax", "TP01", "Alb01", "TG01", "TC01"}, {{107, 5, 0, 16},
  {{95.53571428571429`, 100.`}, {100.`, 76.19047619047619`}, 0.9609375`}}},
{{"Dht", "Dbw", "Dsex", "Dage", "Kiddismax", "Kiddis内",
  "Kiddis外", "RCTaround", "AFAaround", "Subcutmax",
  "TP01", "Alb01", "TG01", "TC01"}, {{107, 5, 0, 16},
  {{95.53571428571429`, 100.`}, {100.`, 76.19047619047619`}, 0.9609375`}}},
{{"Dht", "Dbw", "Dsex", "artery", "Kiddismax", "Kiddis内",
  "Kiddis外", "RCTaround", "AFAaround", "Subcutmax",
  "TP01", "Alb01", "TG01", "TC01"}, {{107, 5, 0, 16},
  {{95.53571428571429`, 100.`}, {100.`, 76.19047619047619`}, 0.9609375`}}},
{{"Dht", "Dbw", "Dage", "artery", "Kiddismax", "Kiddis内",
  "Kiddis外", "RCTaround", "AFAaround", "Subcutmax",
  "TP01", "Alb01", "TG01", "TC01"}, {{107, 5, 0, 16},
  {{95.53571428571429`, 100.`}, {100.`, 76.19047619047619`}, 0.9609375`}}},
{{"Dht", "Dsex", "Dage", "artery", "Kiddismax", "Kiddis内",
  "Kiddis外", "RCTaround", "AFAaround", "Subcutmax",
  "TP01", "Alb01", "TG01", "TC01"}, {{107, 5, 0, 16},
  {{95.53571428571429`, 100.`}, {100.`, 76.19047619047619`}, 0.9609375`}}},
{{"Dbw", "Dsex", "Dage", "artery", "Kiddismax", "Kiddis内",
  "Kiddis外", "RCTaround", "AFAaround", "Subcutmax",
  "TP01", "Alb01", "TG01", "TC01"}, {{107, 5, 0, 16},
  {{95.53571428571429`, 100.`}, {100.`, 76.19047619047619`}, 0.9609375`}}},
{{"Dht", "Dbw", "Dsex", "Dage", "artery", "Kiddismax", "Kiddis内",
  "Kiddis外", "RCTaround", "AFAaround", "Subcutmax",
  "TP01", "Alb01", "TG01", "TC01"}, {{107, 5, 0, 16},
  {{95.53571428571429`, 100.`}, {100.`, 76.19047619047619`}, 0.9609375`}}}};
```
