# Supplementary material for: Preoperative assessment system for hand-assisted laparoscopic donor nephrectomy by discriminant analysis
Source: PLoS One. 2020 Apr 28;15(4):e0227546. doi: 10.1371/journal.pone.0227546 (PMC7188199; doi:10.1371/journal.pone.0227546)
Supplement: S2 Text — (PDF) [file pone.0227546.s004.pdf]

28 kinds of variable combinations with > 94% of total hit rate revealed by cross-validation

■ output form = { { variable1, variable2, variable3, ... }, {estimated total hit rate, estimated HREC, estimated HRDC, estimated sensitivity, estimated specificity}}

□ Dage = donor age, artery = number of renal artery, Kiddismax = maximum thickness of medial perinephric fat,
Kiddis内 = medium thickness of medial perinephric fat, RFAaround = area of perinephric fat, RCTaround = mean CT value of perinephric fat,
AFAaround = area of subcutaneous fat on midline, TP01 = total protein, TC01 = total cholesterol

In[•]:=
```
top28subsets = {{{"Dage", "artery", "Kiddismax", "Kiddis内",
    "RFAaround", "RCTaround", "AFAaround", "TP01", "TC01"},
   {96.09375`, 95.53571428571429`, 100.`, 100.`, 76.19047619047619`}},
  {{"Dage", "artery", "Kiddismax", "Kiddis内", "RFAaround",
    "RCTaround", "AFAaround", "TP01", "Alb01", "TC01"},
   {95.3125`, 94.69026548672566`, 100.`, 100.`, 71.42857142857143`}},
  {{"Dage", "artery", "Kiddismax", "Kiddis内", "RFAaround",
    "RCTaround", "AFAaround", "TP01", "TG01", "TC01"},
   {95.3125`, 94.69026548672566`, 100.`, 100.`, 71.42857142857143`}},
  {{"Dage", "artery", "Kiddismax", "Kiddis内", "RFAaround",
    "RCTaround", "AFAaround", "TP01", "Alb01", "TG01", "TC01"},
   {95.3125`, 94.69026548672566`, 100.`, 100.`, 71.42857142857143`}},
  {{"artery", "Kiddismax", "Kiddis内", "RFAaround", "RCTaround",
    "AFAaround", "TP01", "TC01"}, {94.53125`, 94.64285714285714`,
    93.75`, 99.06542056074767`, 71.42857142857143`}},
  {{"Dbw", "Dage", "artery", "Kiddismax", "Kiddis外", "RCTaround",
    "AFAaround", "TP01", "TC01"}, {94.53125`, 94.64285714285714`,
    93.75`, 99.06542056074767`, 71.42857142857143`}},
  {{"Dage", "Kiddismax", "Kiddis内", "Kiddis外", "RCTaround", "AFAaround",
    "TP01", "Alb01", "TC01"}, {94.53125`, 94.64285714285714`,
    93.75`, 99.06542056074767`, 71.42857142857143`}},
  {{"artery", "Kiddismax", "Kiddis内", "Kiddis外", "RCTaround",
    "AFAaround", "Subcutmax", "TP01", "TC01"}, {94.53125`,
    94.64285714285714`, 93.75`, 99.06542056074767`, 71.42857142857143`}},
  {{"artery", "Kiddismax", "Kiddis内", "RFAaround", "RCTaround",
    "AFAaround", "TP01", "TG01", "TC01"},
   {94.53125`, 93.85964912280701`, 100.`, 100.`, 66.66666666666667`}},
  {{"Dht", "Dbw", "Dage", "artery", "Kiddismax", "Kiddis外", "RCTaround",
    "AFAaround", "TP01", "TC01"}, {94.53125`, 94.64285714285714`,
    93.75`, 99.06542056074767`, 71.42857142857143`}},
  {{"Dht", "Dbw", "artery", "Kiddismax", "Kiddis内", "Kiddis外",
    "RCTaround", "AFAaround", "TP01", "TC01"}, {94.53125`,
```

```
94.64285714285714`, 93.75`, 99.06542056074767`, 71.42857142857143`}},
{{"Dht", "Dage", "Kiddismax", "Kiddis内", "Kiddis外", "RCTaround",
  "AFAaround", "TP01", "Alb01", "TC01"}, {94.53125`,
  94.64285714285714`, 93.75`, 99.06542056074767`, 71.42857142857143`}},
{{"Dbw", "Dage", "Kiddismax", "Kiddis内", "Kiddis外", "RCTaround",
  "AFAaround", "TP01", "Alb01", "TC01"}, {94.53125`,
  94.64285714285714`, 93.75`, 99.06542056074767`, 71.42857142857143`}},
{{"Dage", "artery", "Kiddismax", "Kiddis内", "Kiddis外", "RCTaround",
  "AFAaround", "Subcutmax", "TP01", "TC01"}, {94.53125`,
  94.64285714285714`, 93.75`, 99.06542056074767`, 71.42857142857143`}},
{{"Dage", "artery", "Kiddismax", "Kiddis内", "Kiddis外", "RCTaround",
  "AFAaround", "TP01", "TG01", "TC01"}, {94.53125`, 94.64285714285714`,
  93.75`, 99.06542056074767`, 71.42857142857143`}},
{{"Dage", "artery", "Kiddismax", "Kiddis内", "RFAaround",
  "RCTaround", "AFAaround", "Subcutmax", "TP01", "TC01"},
 {94.53125`, 93.85964912280701`, 100.`, 100.`, 66.66666666666667`}},
{{"Dage", "artery", "Kiddismax", "Kiddis内", "RFAaround",
  "RCTaround", "AFAaround", "Subcutmax", "Alb01", "TC01"},
 {94.53125`, 93.85964912280701`, 100.`, 100.`, 66.66666666666667`}},
{{"Dage", "Kiddismax", "Kiddis内", "Kiddis外", "RCTaround",
  "AFAaround", "TP01", "Alb01", "TG01", "TC01"}, {94.53125`,
  94.64285714285714`, 93.75`, 99.06542056074767`, 71.42857142857143`}},
{{"artery", "Kiddismax", "Kiddis内", "Kiddis外", "RCTaround",
  "AFAaround", "Subcutmax", "TP01", "TG01", "TC01"}, {94.53125`,
  94.64285714285714`, 93.75`, 99.06542056074767`, 71.42857142857143`}},
{{"artery", "Kiddismax", "Kiddis内", "Kiddis外", "RCTaround",
  "AFAaround", "TP01", "Alb01", "TG01", "TC01"}, {94.53125`,
  94.64285714285714`, 93.75`, 99.06542056074767`, 71.42857142857143`}},
{{"Dht", "Dbw", "Dage", "Kiddismax", "Kiddis内", "Kiddis外",
  "RCTaround", "AFAaround", "TP01", "Alb01", "TC01"}, {94.53125`,
  94.64285714285714`, 93.75`, 99.06542056074767`, 71.42857142857143`}},
{{"Dht", "Dage", "artery", "Kiddismax", "Kiddis内", "Kiddis外",
  "RCTaround", "AFAaround", "TP01", "TG01", "TC01"},
 {94.53125`, 93.85964912280701`, 100.`, 100.`, 66.66666666666667`}},
{{"Dht", "artery", "Kiddismax", "Kiddis内", "Kiddis外", "RCTaround",
  "AFAaround", "TP01", "Alb01", "TG01", "TC01"}, {94.53125`,
  94.64285714285714`, 93.75`, 99.06542056074767`, 71.42857142857143`}},
{{"Dbw", "Dage", "artery", "Kiddismax", "Kiddis内", "Kiddis外",
  "RCTaround", "AFAaround", "TP01", "TG01", "TC01"},
 {94.53125`, 93.85964912280701`, 100.`, 100.`, 66.66666666666667`}},
{{"Dbw", "Dage", "Kiddismax", "Kiddis内", "Kiddis外", "RCTaround",
  "AFAaround", "Subcutmax", "TP01", "Alb01", "TC01"}, {94.53125`,
  94.64285714285714`, 93.75`, 99.06542056074767`, 71.42857142857143`}},
{{"Dage", "artery", "Kiddismax", "Kiddis内", "RFAaround",
  "RCTaround", "AFAaround", "Subcutmax", "TP01", "TG01", "TC01"},
```

```
 {94.53125`, 93.85964912280701`, 100.`, 100.`, 66.66666666666667`}},
{{"Dht", "Dbw", "Dage", "artery", "Kiddismax", "Kiddis内",
  "Kiddis外", "RCTaround", "AFAaround", "TP01", "TG01", "TC01"},
 {94.53125`, 93.85964912280701`, 100.`, 100.`, 66.66666666666667`}},
{{"Dbw", "Dage", "artery", "Kiddismax", "Kiddis内", "Kiddis外",
  "RCTaround", "AFAaround", "TP01", "Alb01", "TG01", "TC01"},
 {94.53125`, 93.85964912280701`, 100.`, 100.`, 66.66666666666667`}}};
```
