# Supplementary material for: Preoperative assessment system for hand-assisted laparoscopic donor nephrectomy by discriminant analysis
Source: PLoS One. 2020 Apr 28;15(4):e0227546. doi: 10.1371/journal.pone.0227546 (PMC7188199; doi:10.1371/journal.pone.0227546)
Supplement: S3 Text — (PDF) [file pone.0227546.s005.pdf]

True discrimination rates estimated by .632 estimator with 28 kinds of variable combinations

■ output form = { { variable1, variable2, variable3, ... }, {estimated total hit rate, estimated HREC, estimated HRDC, estimated sensitivity, estimated specificity}}

□ Dage = donor age, artery = number of renal artery, Kiddismax = maximum thickness of medial perinephric fat,
Kiddis内 = medium thickness of medial perinephric fat, RFAaround = area of perinephric fat, RCTaround = mean CT value of perinephric fat,
AFAaround = area of subcutaneous fat on midline, TP01 = total protein, TC01 = total cholesterol

In[•]:=
```
finalresult =
  {{{"Dage", "artery", "Kiddismax", "Kiddis内", "RFAaround", "RCTaround",
     "AFAaround", "TP01", "TC01"}, {94.59315786672344`, 94.46229451819245`,
     95.46077348066298`, 99.3739395478791`, 69.94967418546366`}},
   {{"artery", "Kiddismax", "Kiddis内", "RFAaround", "RCTaround",
     "AFAaround", "TP01", "TC01"}, {94.47036540706931`, 94.31550323699621`,
     95.5266787658802`, 99.37322314049587`, 69.62986901701555`}},
   {{"Dage", "artery", "Kiddismax", "Kiddis内", "RFAaround",
     "RCTaround", "AFAaround", "TP01", "Alb01", "TG01", "TC01"},
    {94.2880715495343`, 94.16503538443085`, 95.10783078307831`,
     99.30937738246506`, 69.09005975013581`}},
   {{"Dage", "artery", "Kiddismax", "Kiddis内", "RFAaround",
     "RCTaround", "AFAaround", "TP01", "Alb01", "TC01"},
    {94.15569727891156`, 93.9228516694034`, 95.81060606060606`,
     99.43542623787647`, 67.67880770628862`}},
   {{"Dbw", "Dage", "artery", "Kiddismax", "Kiddis外", "RCTaround",
     "AFAaround", "TP01", "TC01"}, {94.15382284134411`, 94.15594295313518`,
     93.97532710280375`, 99.18213651357523`, 68.23362743257618`}},
   {{"Dage", "artery", "Kiddismax", "Kiddis内", "RFAaround",
     "RCTaround", "AFAaround", "TP01", "TG01", "TC01"},
    {93.98171434671221`, 93.95102606003269`, 94.05456255879587`,
     99.19243547150523`, 67.50735593848435`}},
   {{"Dage", "Kiddismax", "Kiddis内", "Kiddis外", "RCTaround", "AFAaround",
     "TP01", "Alb01", "TC01"}, {93.97573497504882`, 94.04115909929864`,
     93.3961503208066`, 99.06260733801717`, 68.34253388039289`}},
   {{"artery", "Kiddismax", "Kiddis内", "RFAaround", "RCTaround", "AFAaround",
     "TP01", "TG01", "TC01"}, {93.95586778639104`, 93.90805923048914`,
     94.15595805529075`, 99.20959257349149`, 67.3112022877036`}},
   {{"Dage", "artery", "Kiddismax", "Kiddis内", "Kiddis外",
     "RCTaround", "AFAaround", "Subcutmax", "TP01", "TC01"},
    {93.94188825535544`, 94.09234663027141`, 92.73824057450628`,
     98.96803163668835`, 68.39768073032322`}},
   {{"Dht", "Dage", "Kiddismax", "Kiddis内", "Kiddis外", "RCTaround", "AFAaround",
```

```
"TP01", "Alb01", "TC01"}, {93.9181099117303`, 94.22355397357794`,
  91.31374407582939`, 98.84686045048447`, 67.53809523809524`}},
{{"Dht", "Dbw", "artery", "Kiddismax", "Kiddis内", "Kiddis外", "RCTaround",
  "AFAaround", "TP01", "TC01"}, {93.83163686014136`, 94.04997479093863`,
  92.16342551293488`, 98.87316572601334`, 68.28608486196035`}},
{{"artery", "Kiddismax", "Kiddis内", "Kiddis外", "RCTaround", "AFAaround",
  "Subcutmax", "TP01", "TC01"}, {93.75175373134329`, 93.81523523866655`,
  93.04497607655502`, 99.07425805629856`, 66.47890622566881`}},
{{"Dht", "Dbw", "Dage", "artery", "Kiddismax", "Kiddis外", "RCTaround",
  "AFAaround", "TP01", "TC01"}, {93.70352861970642`, 93.81162758996729`,
  92.71889400921658`, 99.00075891727802`, 66.85575358742346`}},
{{"Dbw", "Dage", "Kiddismax", "Kiddis内", "Kiddis外", "RCTaround", "AFAaround",
  "TP01", "Alb01", "TC01"}, {93.63870789841816`, 93.80232606510208`,
  92.18268315889628`, 98.94368732321934`, 66.45868929750118`}},
{{"Dage", "artery", "Kiddismax", "Kiddis内", "RFAaround",
  "RCTaround", "AFAaround", "Subcutmax", "TP01", "TC01"},
 {94.27786896219342`, 94.23987901349464`, 94.39160305343512`,
  99.25049732211171`, 68.41000159261029`}},
{{"Dage", "artery", "Kiddismax", "Kiddis内", "RFAaround",
  "RCTaround", "AFAaround", "Subcutmax", "Alb01", "TC01"},
 {94.27193195140147`, 94.32635500769351`, 93.79285714285714`,
  99.11394341065511`, 69.56834897914923`}},
{{"Dage", "artery", "Kiddismax", "Kiddis内", "Kiddis外",
  "RCTaround", "AFAaround", "TP01", "TG01", "TC01"},
 {94.0931975249116`, 94.34749843434695`, 92.16342551293488`,
  98.87805874840357`, 69.33044387415646`}},
{{"Dht", "Dbw", "Dage", "Kiddismax", "Kiddis内", "Kiddis外",
  "RCTaround", "AFAaround", "TP01", "Alb01", "TC01"},
 {93.91007115343461`, 94.10673909674753`, 92.16777883698761`,
  98.95590291015377`, 67.36158880613857`}},
{{"artery", "Kiddismax", "Kiddis内", "Kiddis外", "RCTaround", "AFAaround",
  "TP01", "Alb01", "TG01", "TC01"}, {93.90763763418005`, 94.14380952380952`,
  92.15524475524475`, 98.85763747454175`, 68.81491429797933`}},
{{"Dage", "Kiddismax", "Kiddis内", "Kiddis外", "RCTaround", "AFAaround",
  "TP01", "Alb01", "TG01", "TC01"}, {93.8334780865847`, 94.03936776645362`,
  92.13640552995392`, 98.91173469387755`, 67.66845827439887`}},
{{"artery", "Kiddismax", "Kiddis内", "Kiddis外", "RCTaround",
  "AFAaround", "Subcutmax", "TP01", "TG01", "TC01"},
 {93.62012711864406`, 93.86811342391493`, 91.66909090909091`,
  98.82256199408968`, 67.25576905226028`}},
{{"Dht", "Dage", "artery", "Kiddismax", "Kiddis内", "Kiddis外",
  "RCTaround", "AFAaround", "TP01", "TG01", "TC01"},
 {93.97409914712154`, 94.11588984509466`, 92.7437037037037`,
  99.00383882038896`, 67.97147263399742`}},
{{"Dbw", "Dage", "artery", "Kiddismax", "Kiddis内", "Kiddis外",
```

```
  "RCTaround", "AFAaround", "TP01", "TG01", "TC01"},
 {93.97383198681833`, 93.99432866592217`, 93.69157509157509`,
  99.12244585987261`, 67.93893017708047`}},
{{"Dage", "artery", "Kiddismax", "Kiddis内", "RFAaround",
  "RCTaround", "AFAaround", "Subcutmax", "TP01", "TG01", "TC01"},
 {93.76531840503897`, 93.74516882492358`, 93.73422306959009`,
  99.15895073576455`, 66.52005400098184`}},
{{"Dht", "artery", "Kiddismax", "Kiddis内", "Kiddis外", "RCTaround",
  "AFAaround", "TP01", "Alb01", "TG01", "TC01"},
 {93.7061355896126`, 93.9833353358164`, 91.40055917986952`,
  98.83096414544534`, 67.01773595665811`}},
{{"Dbw", "Dage", "Kiddismax", "Kiddis内", "Kiddis外", "RCTaround",
  "AFAaround", "Subcutmax", "TP01", "Alb01", "TC01"},
 {93.64241397849462`, 93.87351443123939`, 91.665275142315`,
  98.87374358974358`, 66.59009523809523`}},
{{"Dht", "Dbw", "Dage", "artery", "Kiddismax", "Kiddis内",
  "Kiddis外", "RCTaround", "AFAaround", "TP01", "TG01", "TC01"},
 {93.5593077129084`, 93.69990023221811`, 92.2073786407767`,
  98.97360613810741`, 65.70779820839228`}},
{{"Dbw", "Dage", "artery", "Kiddismax", "Kiddis内", "Kiddis外",
  "RCTaround", "AFAaround", "TP01", "Alb01", "TG01", "TC01"},
 {93.52940831556504`, 93.71602384110524`, 91.97644894204232`,
  98.88499105088214`, 66.534115777248`}}};
```
